# Nipah virus survey in *Pteropus medius* of eastern and northeastern region of India, 2022–2023

**DOI:** 10.3389/fmicb.2024.1493428

**Published:** 2024-12-24

**Authors:** Sreelekshmy Mohandas, Dilip Patil, Basavaraj Mathapati, Vishal Rai, Anita Shete, Sujeet Belani, Abhinendra Kumar, Rima Sahay, Deepak Patil, Pragya D. Yadav

**Affiliations:** ^1^Maximum Containment Facility, ICMR-National Institute of Virology, Pune, India; ^2^Animal House Division, ICMR-National Institute of Virology, Pune, India; ^3^Field Unit, ICMR-National Institute of Virology, Dibrugarh, India; ^4^National Institute of One Health, Nagpur, India

**Keywords:** Nipah virus, eastern India, *Pteropus medius*, Bihar, West Bengal, Assam, Meghalaya

## Abstract

**Introduction:**

India has experienced seven outbreaks of the Nipah virus (NiV) since 2001, primarily occurring in the southern and eastern regions of the country. The southern region has been the main site for these outbreaks. In contrast, the eastern region, which borders Bangladesh, has not reported any outbreaks since 2007. However, Bangladesh continues to experience nearly annual outbreaks, indicating a significant lack of surveillance in that area. To improve the country’s preparedness and to gather support for enhancing public health surveillance in eastern and northeastern states near the area affected by the NiV, a cross-sectional survey was conducted to determine the prevalence of NiV in the bat species *Pteropus medius* in Bihar, West Bengal, Assam, and Meghalaya states in India, which are adjacent to Bangladesh.

**Methods:**

Throat and rectal swabs, blood samples, and organ samples were collected. Real-time quantitative reverse transcription PCR (qRT-PCR) was utilized for the detection of Nipah viral RNA, and sequencing was conducted for further confirmation. Bat IgG enzyme-linked immunosorbent assay (ELISA) was employed for antibody detection.

**Results:**

Throat and rectal swab samples of 212 *P. medius* tested for NiV using qRT- PCR were found negative, whereas organ samples of two (one each from West Bengal and Bihar) out of the 10 bats collected tested positive. The retrieved NiV genome (~91%) showed close homology to the NiV-Bangladesh genotype indicating the circulation of two geographically distinct NiV strains in India. The seroprevalence estimated by ELISA ranged from 23 to 65% in the studied states.

**Discussion:**

The serological and virological evidence obtained from the study indicates that a broader geographical area is under threat of spillover in India. It’s crucial to implement a One Health approach connecting bat surveillance studies with human surveillance and risk factor studies in the region.

## 1 Introduction

Nipah virus (NiV) is a paramyxovirus that causes a highly fatal viral infection characterized by severe respiratory and neurological signs in humans. The virus is considered a priority pathogen by the World Health Organization (WHO, n.d.). The NiV is a negative sense single-stranded RNA virus with the genome coding for six structural and 3 non-structural proteins. The nucleoprotein (N), phosphoprotein (P), glycoprotein (G), matrix (M), fusion (F), and RNA polymerase (L) constitute the structural proteins. The P gene encodes for three non-structural proteins, i.e., V, W, and C. The NiV strains fall into separate clades like Malaysia, Bangladesh, and India. The Bangladesh and Indian clades differ by about 9% in nucleotide substitutions from Malaysian strains (Whitmer et al., 2021).

Outbreaks of the NiV disease have been reported in several countries, including Malaysia, Singapore, Bangladesh, India, and the Philippines (Chua et al., 1999; Paton et al., 1999; Chadha et al., 2006; Rahman and Chakraborty, 2012; Ching et al., 2015). The disease was first identified in Malaysia in the late 1990s, where it spread from fruit bats to pig populations and then to humans (Chua et al., 1999). In Singapore, the disease was reported among abattoir workers handling pigs imported from Malaysia during the same period (Paton et al., 1999). In the Philippines, henipavirus infection was reported among people who came in contact with sick horses (Ching et al., 2015). Unlike Malaysia, Singapore and Philippines, multiple outbreaks of NiV disease have been reported in Bangladesh and India. Since 2001, Bangladesh has been facing annual outbreaks in the northwestern districts, known as the “Nipah belt, “and Kerala state in India has been experiencing frequent outbreaks since 2018 (Arunkumar et al., 2019; Sudeep et al., 2021; Yadav et al., 2022; As et al., 2024; PIB, 2024; Nipah virus infection, n.d.).

**Figure 1:**
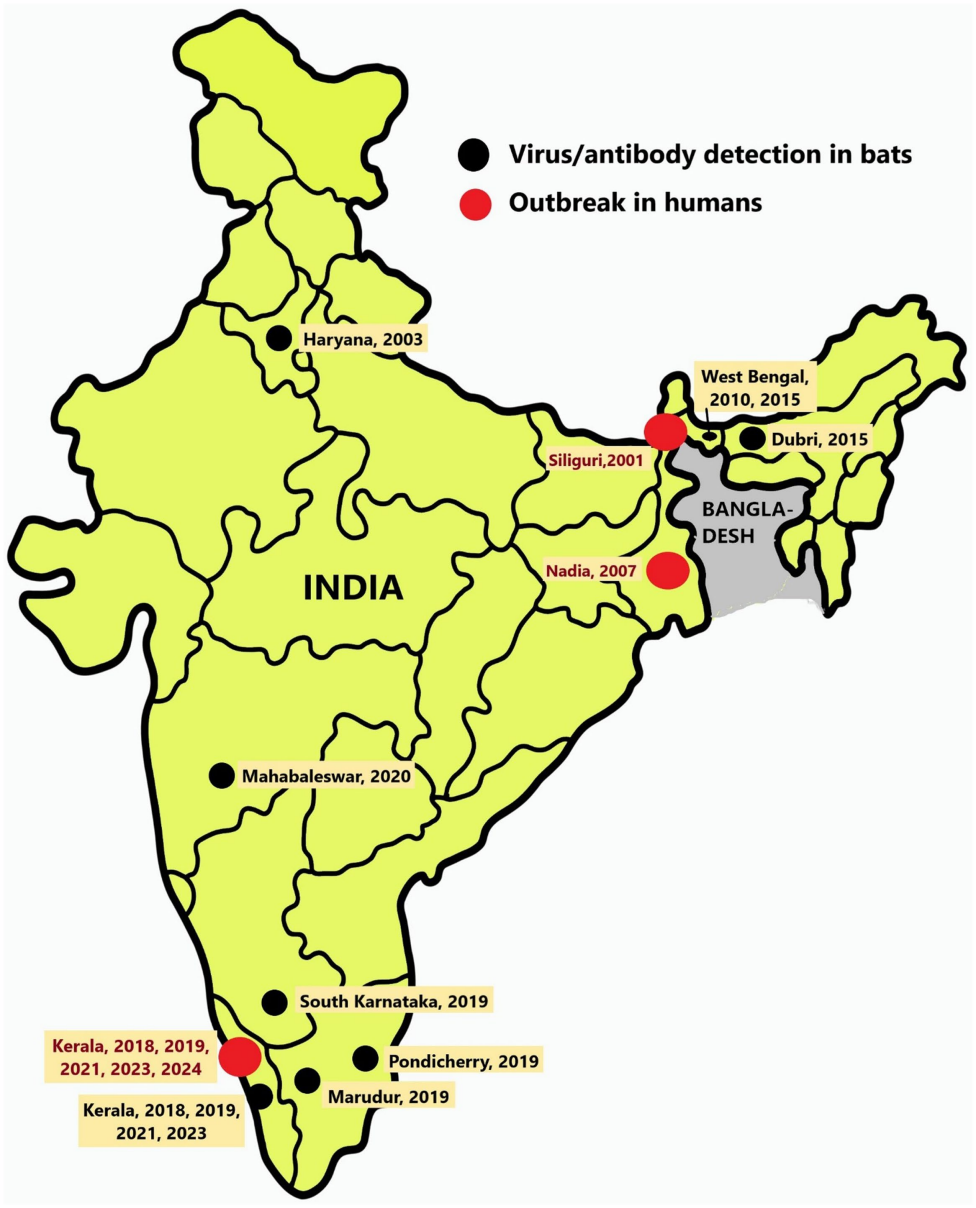
Location of Nipah virus outbreaks and detection in bats: Map of India showing the locations of Nipah virus outbreaks in humans (red dots) and the regions from where bats were found harboring Nipah virus (black dots).

There have been seven known outbreaks of the NiV disease in India. Two of them occurred in the Siliguri and Nadia districts of West Bengal, which are located near the border with Bangladesh, in the years 2001 and 2007, respectively (Chadha et al., 2006; Arankalle et al., 2011). The other five outbreaks in India occurred in the Kozhikode, Ernakulam, and Malappuram districts of the southern Indian state of Kerala after 2018 (Figure 1, Table 1) (Arunkumar et al., 2019; Sudeep et al., 2021; Yadav et al., 2022; As et al., 2024). Surveys conducted in various species of bats in India have detected the presence of NiV RNA in *Pteropus medius*, Rousettus sp., and Pipistrellus sp. (Table 1 and Figure 1).

**Table 1:** The timeline of Nipah virus outbreaks and virus detection in bats of India.

Year	Report	Reference
Human outbreaks		
2001	66 cases reported from Siliguri, West Bengal	Chadha et al. (2006)
2007	5 cases reported from Nadia, West Bengal	Arankalle et al. (2011)
2018	23 cases reported from Kozhikode and Malappuram, Kerala	Arunkumar et al. (2019)
2019	1 case reported from Ernakulam, Kerala	Sudeep et al. (2021)
2021	1 case reported from Kozhikode, Kerala	Yadav et al. (2022)
2023	6 cases reported from Kozhikode, Kerala	As et al. (2024)
2024	2 cases reported from Malappuram, Kerala	PIB (2024)
Nipah virus detection in bats		
2003	Seropositivity in *Pteropus medius* bats from Haryana	Epstein et al. (2008)
2009–10	Viral RNA and seropositivity in *P. medius* from Myanaguri, West Bengal	Yadav et al. (2012)
2015	Viral RNA detection in *P. medius* from Cooch Behar, West Bengal and Dhubri, Assam	Yadav et al. (2018)
2018	Viral RNA detection in *P. medius* from Kozhikode Kerala	Yadav et al. (2019)
2019	Viral RNA detection in *P. medius* from Ernakulam and Idukki, Kerala	Sudeep et al. (2021)
2019–20	Seropositivity in *P. medius* in the southern part of Karnataka, Tamil Nadu, and Pondicherry	Gokhale et al. (2022)
2020	Viral RNA and seropositivity Rousettus sp. and Pipistrellus sp. from Maharashtra	Gokhale et al. (2022)
2023	Viral RNA detection in *P. medius* from Wayanad, Kerala	Balasubramanian et al. (2024)

Fruit bats belonging to the *Pteropus* species act as reservoir hosts for the NiV and play a crucial role in disease maintenance and transmission (Epstein et al., 2020). The ecological and economic importance of fruit bats has been widely documented. They contribute to pollination, seed dispersal, pest control, predation, and nutrient distribution across ecosystems, serving as an important ecological indicator (Kasso and Balakrishnan, 2013). The serological or molecular evidence of henipavirus infection has been detected in Pteropid bats in countries that have experienced outbreaks and in other countries, including Cambodia, China, East Timor, Indonesia, Madagascar, Papua New Guinea, Thailand, and Vietnam (Reynes et al., 2005; Wacharapluesadee et al., 2005; Iehlé et al., 2007; Li et al., 2008; Breed et al., 2010; Sendow et al., 2010; Hasebe et al., 2012).

Bangladesh shares 75% of its international border with the eastern and northeastern states of India, and the region known as the Nipah belt is near India. The surveys conducted on *P. medius* bats in eight districts of Bangladesh between 2006 and 2012 revealed virus detection throughout the country (Epstein et al., 2016, 2020). This included bat colonies from Thakurgaon [~30 km from the India (West Bengal) border], Kushtia [~30 km from the India (West Bengal) border], Cumilla [~2 km from India (Tripura) border], Sylhet [~30 km from India (Meghalaya) border], Chattogram [~70 km from India (Tripura/Mizoram) border], and Khulna [~70 km from India (West Bengal) border] (Epstein et al., 2020). Even though Bangladesh reports outbreaks annually, there are no reports of NiV human outbreaks after 2007 from the state of West Bengal or other neighboring states of India bordering Bangladesh (Nipah virus infection, n.d.).

The Nipah sentinel surveillance system established in 2006 in Bangladesh has helped detect Nipah virus cases and contain outbreaks in the country (Satter et al., 2023). However, India lacks a similar coordinated surveillance system. Syndromic surveillance has been strengthened in the state of Kerala following the highly fatal outbreak in 2018, leading to an effective containment response in the subsequent years (Yadav et al., 2022; As et al., 2024). Here we present our findings of the cross-sectional survey in *P. medius* taken up in the states (West Bengal, Meghalaya, and Assam) sharing the borders with Bangladesh and an adjoining state, Bihar, between 2022 and 2023. These crucial efforts were focused on advocating for the enhancement of public health surveillance activities in the states neighboring the Nipah belt.

## 2 Materials and methods

### 2.1 Sample collection

**Figure 2:**
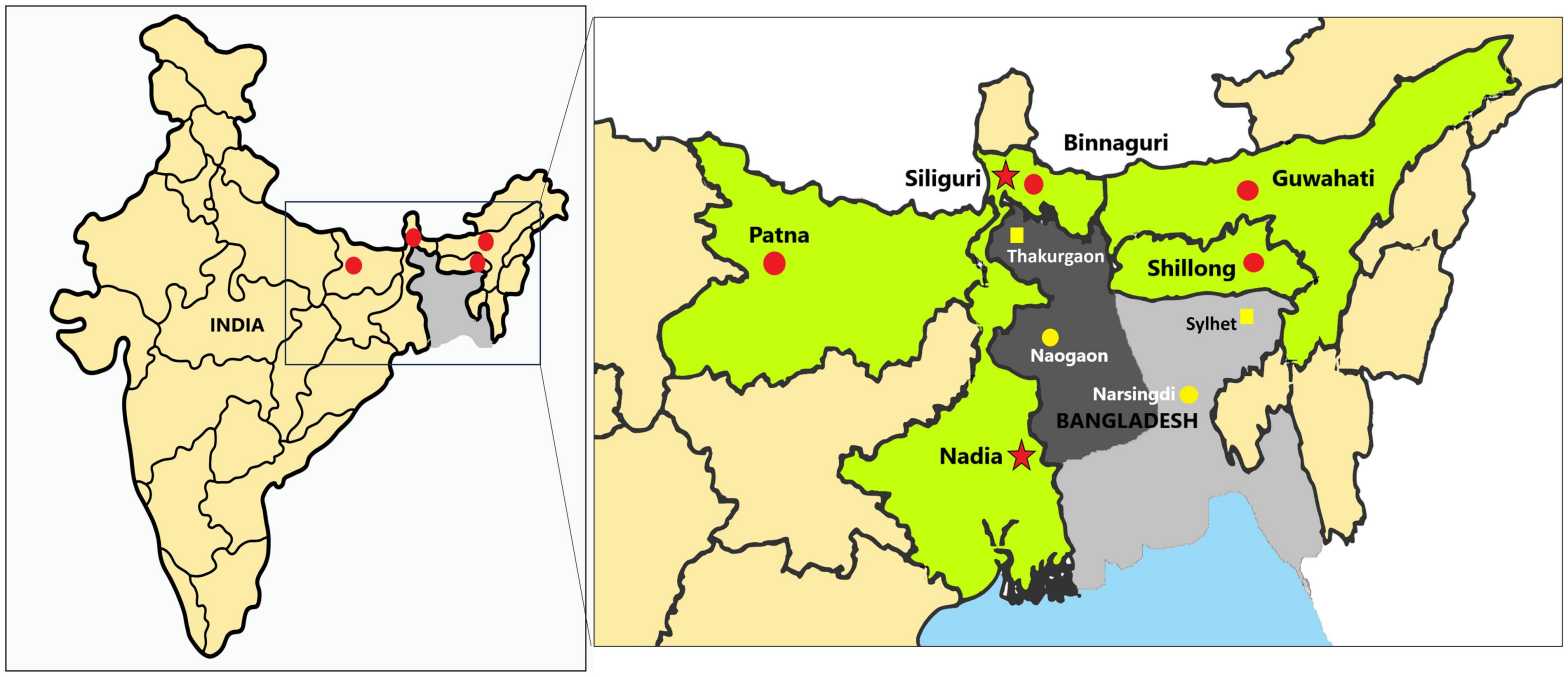
Sample collection sites of the study: The left map of India shows the sampled sites marked as red dots, while the right magnified image displays the sampled sites (red dots) in each state (marked as green). The red dots on the map indicate the locations of the sampling sites in the states of Bihar (Patna), West Bengal (Binnaguri), Assam (Guwahati), and Meghalaya (Shillong) in India. The red stars denote the previous Nipah virus outbreak locations in India, i.e., Siliguri and Nadia. The dark grey region in the central and northwest areas of Bangladesh is known as the Nipah belt, while the rest of the country is colored light grey. The yellow dots mark the cities of Naogaon and Narsingdi (used to measure the approximate distance to each sampling site). Thakurgaon and Sylhet in Bangladesh are marked as yellow squares places in Bangladesh that have reported NiV positivity in bats near sampling locations in India.

The Institute Animal Ethics Committee of ICMR-National Institute of Virology, Pune, and the Principal Chief Conservator of Forests of Meghalaya, Assam, West Bengal, and Bihar approved the study. The national guidelines of the Committee for Control and Supervision of Experiments on Animals, Government of India followed for the care and use of animals in the study. The bat samples were collected from Patna, Bihar (~400 km from Naogaon, the nearest location in Bangladesh from where NiV cases were reported in 2023 and ~ 350 km to Siliguri, West Bengal from where NiV outbreaks reported in India), Binnaguri, West Bengal (~200 km from Naogaon and ~ 60 km from Siliguri, West Bengal); from Guwahati, Assam (~260 km from Narsingdi, nearest location in Bangladesh from where NiV cases are reported in 2023) and Shillong, Meghalaya (~200 km from Narsingdi) (Figure 2).

Mist nets were set up on poles near the bat roosts to trap the bats. The bats were identified by their morphological features and their body weight, sex, and reproductive status were recorded. The bats were classified into juveniles and adults based on their body weight and secondary sexual characteristics (Epstein et al., 2020). A total of 212 *P. medius* were trapped which included 43 adult bats from Shillong, Meghalaya, 53 adult bats from Guwahati, Assam, 60 bats (Adults =49, Juveniles =11) from Binnaguri, West Bengal, and 56 bats (Adults = 39; Juveniles = 10) from Patna, Bihar (Table 2).

**Table 2:** The details of the *P. medius* samples collected in the study from different sites.

Sr. no.	Details of bats sampled						No. of bats from which throat/rectal swabs collected	No. of bats collected	No. of bats from which blood collected
	Month, Year	Location	Total no.	No. of juveniles	Sex	Body weight in grams (Average ± SD)			
1	November 2022	Shillong, Meghalaya	43	0	Male: 25 Female: 18	581 **±** 135,619 ± 114	43	2	34
2	November, 2022	Guwahati, Assam	53	0	Male: 29 Female: 24	676 ± 171,596 ± 124	53	2	30
3	May, 2023	Binnaguri, West Bengal	60	11*	Male: 33 Female: 26	555 ± 228,457 ± 152	60	3	54
4	May, 2023	Patna, Bihar	56	10†	Male: 29 Female: 27	573 ± 254,547 ± 193	56	3	35

^*^Serum collected from one juvenile out of the eleven sampled. ^†^ Serum collected from 4 juveniles out of the 10 sampled.

The captured bats were anesthetized using isoflurane and swabbed (throat and rectal) for testing, and blood samples were also collected. The swabs were collected using a sterile swab applicator in a virus transport medium (Himedia, India). Blood samples (2 mL) were collected from the wing vein using a sterile 25G needle in a serum separation gel tube and serum was separated by centrifugation after 45 min. The bats were released to their natural habitat after recovery from anesthesia. Few bats (2 bats from Assam and Bihar each, 3 bats each from Meghalaya, and West Bengal) which were injured during trapping were euthanized and transported to the containment laboratory in liquid nitrogen for organ collection. The kidney, spleen, liver, intestine, and reproductive organs were collected for NiV RNA testing.

### 2.2 Real-time quantitative reverse transcription PCR (qRT-PCR)

The RNA was extracted from the samples using the magnetic bead-based method in the Kingfisher Flex machine (Thermo Fischer Scientific, USA) using the Magmax Viral RNA isolation kit (Thermo Scientific, USA) as per the manufacturer’s instructions. The real-time qRT-PCR was carried out with published NiV-specific primers and probes targeting the N gene (Guillaume et al., 2004).

### 2.3 Partial and whole genome sequencing

The qRT-PCR positive samples were further confirmed by Sanger sequencing. For sequencing, a nested RT-PCR was performed with the primers (1st PCR: forward: CGTGGTTATCTTGAACCTATGTACTTCAG and reverse: CGCAACTTTAATGTAATTGGTCCCTTAGTG, 2nd PCR: forward: CAGAGAAGCTAAATTTGCTGCAGGAGG and Reverse: TCACACATCAGCTCTGACAAAGTCAAG) specific for the NiV N-gene. The PCR products were electrophoresed on 1.5% agarose gel and bands (342 bp) were purified using the QIAquick Gel Extraction Kit (Qiagen, Germany). The Big Dye™ Terminator Cycle Sequencing Kit (Applied Biosystems, USA) was used for sequencing PCR and the DyeEx 2.0 kit (Qiagen, Germany) was used for the PCR product purification. The ABI 3130 XL genetic analyzer (Applied Biosystems, USA) was used for sequencing and the Sequencher 5.1 software (Accelrys Inc., USA) was used for analysis. The retrieved partial NiV N gene sequences were submitted to GenBank and the accession number was obtained.

The whole genome sequencing for the Nipah virus qRT-PCR positive samples was performed using the Virus Surveillance Panel (VSP) Illumina RNA Prep with Enrich A kit (Illumina, USA). A total of 8.5 microliters of extracted RNA was denatured and combined with random hexamers in a thermal cycler at 65°C for 5 min. Following this, the hexamer-primed RNA fragments underwent reverse transcription by adding the reverse transcriptase and first strand synthesis master mix in the thermal cycler. The conditions for this step were as follows: 25°C for 10 min, 42°C for 15 min, and 70°C for 15 min. In the subsequent step, the RNA template was removed, and a replacement strand was synthesized to generate blunt-ended double-stranded cDNA fragments. The cDNA was tagmented using enrichment bead-linked transposomes after purification. The tagmented cDNA were purified and amplified to add adapter sequences for dual indexing, and P7 and P5 sequences for clustering. Four index sets (A, B, C, and D), each containing 96 unique, single-use Illumina® DNA/RNA UD indexes, were used. Libraries were quantified using the Invitrogen Qubit dsDNA HS Assay Kit (Thermo Fischer Scientific, USA). Subsequently, libraries (200 ng library or 600 ng pool) were hybridized using oligos from the VSP panel, followed by bead-based capture of hybridized probes, amplification, clean-up, and quantification. Normalized libraries, diluted to an equimolar concentration of 4 pM, were then pooled. Using a 150 bp paired-end sequencing approach and 300 cycles, the libraries were sequenced on the Miniseq sequencer (Illumina, USA) utilizing a high output kit (Illumina, USA). The fastq (raw read files) were mapped with the Nipah virus (NC_002728.1) reference strain using CLC genomics 24.02 software. The coverage above 10X and mutation frequency above 50% were considered for generating variant calling files. Clustal Omega was used to visualize and represent the nucleotide changes.

### 2.4 Phylogenetic analysis

The phylogenetic analysis of the sequence retrieved in the study was performed with the reference sequences of Nipah virus (NC_002728.1) along with a subset of NiV whole genome sequences from the NCBI database. The analysis included 7 full genome sequences (6 human and 1 bat) from India, 17 (16 human and 1 bat) from Bangladesh, 1 (bat) from Cambodia, and 7 (3 human and 4 swine) from Malaysia. For alignment and visualization, we utilized the MAFFT tool and MEGAX (Katoh et al., 2019). We used IQTREE with the Tamura-Nei + F model and 1,000 ultrafast bootstrap replicates to generate the maximum likelihood tree (Minh et al., 2020). The phylogenetic tree was visualized using the Interactive Tree of Life (iTOL) (Letunic and Bork, 2021).

### 2.5 Virus isolation

Vero cells (ATCC^®^ CCL-81™, ATCC, USA) and infant CD1 (ICR) laboratory mice (3–4 days old) were used for virus isolation. The CD1 mouse is an outbred mouse strain procured from the CCSEA-licensed laboratory animal house of ICMR-National Institute of Virology, Pune. The samples were inoculated to 24-well plates with Vero cells and were incubated for 1 h. After washing, tissue culture media with 2% fetal bovine serum were added to each well, and plates were incubated in a CO_2_ incubator for 5 days. The samples were blindly passaged three times. Simultaneously, samples were also intracranially inoculated in the mouse model and the animals were observed for sickness. Brain samples were collected 7 days after inoculation from mice and were homogenized in sterile media. The cell culture supernatants and the mice brain suspension were tested for NiV by real-time qRT-PCR after each passage.

### 2.6 Anti-Nipah bat IgG ELISA

*P. medius* bat sera samples (*n* = 153) were tested by an in-house enzyme-linked immunosorbent assay as described earlier (Gokhale et al., 2022). Heat-inactivated bat sera samples were added to gamma-irradiated whole virion NiV antigen-coated plates and were incubated at 37°C for 1 h. After washing, anti-bat IgG HRP conjugate was added to the plate and incubated for 1 h. After washing, 3,3′,5,5′-Tetramethylbenzidine substrate was added and plates were kept for incubation at 37°C for half an hour. Using 1 N H_2_SO_4_, the color reaction was stopped. The plate was read at 450 nm. The serum samples with an optical density (OD) value of ≥0.3 were considered positive for anti-Nipah IgG antibodies.

### 2.7 Serum neutralization test

A micro-neutralization test (MNT) was conducted on a subset of bat serum samples found positive (*n* = 19) and negative (*n* = 6) by IgG ELISA. The assay was performed in Vero (ATCC^®^ CCL − 81™) cell monolayers in 96-well cell culture plates in the containment facility. The bat serum samples were treated by heat inactivation at 56°C for 1 h. Dilutions (two-fold) of the bat sera were prepared by using a sterile tissue culture media. The virus-serum mixture prepared in 1:1 dilution with 100 median tissue culture infectious dose of Nipah virus (GenBank accession ID: MH523642) was then incubated at 37°C for 1 h in a CO_2_ incubator and was then added to the cell monolayer in 96-well plates in duplicate. The assay included positive (anti-Nipah IgG positive mice serum), negative (anti-Nipah IgG negative mice serum), and cell controls. After an incubation period of 5 days, the cells were observed for cytopathic changes, and the neutralization titer was calculated.

## 3 Results

### 3.1 Viral RNA detection in *P. medius* by qRT-PCR

The throat and rectal swab samples of all the bats collected during the study (*n* = 212) were negative for viral RNA whereas viral RNA could be detected in the organ samples of two bats out of the 10 bats from which organs were collected. Kidney (Viral RNA copy number = 1.26 × 10^4^/ml) and spleen (Viral RNA copy number = 9.5 × 10^4^/ml) samples of one bat collected from Binnaguri, West Bengal, and spleen (Viral RNA copy number = 2.7 × 10^5^/ml) sample of one bat collected from Patna, Bihar were found positive by qRT-PCR.

### 3.2 Partial and whole genome sequencing

By Sanger’s sequencing, a partial N gene (GenBank Accession no. OR765959) sequence was retrieved from one spleen sample (from Patna, Bihar) out of the three found positive by real-time qRT-PCR. By whole genome sequencing, 91.9% of the NiV genome (GenBank Accession no. PP554504) could be retrieved from the above sample (Supplementary Figure S1, Supplementary Table S1).

### 3.3 Phylogenetic analysis

**Figure 3:**
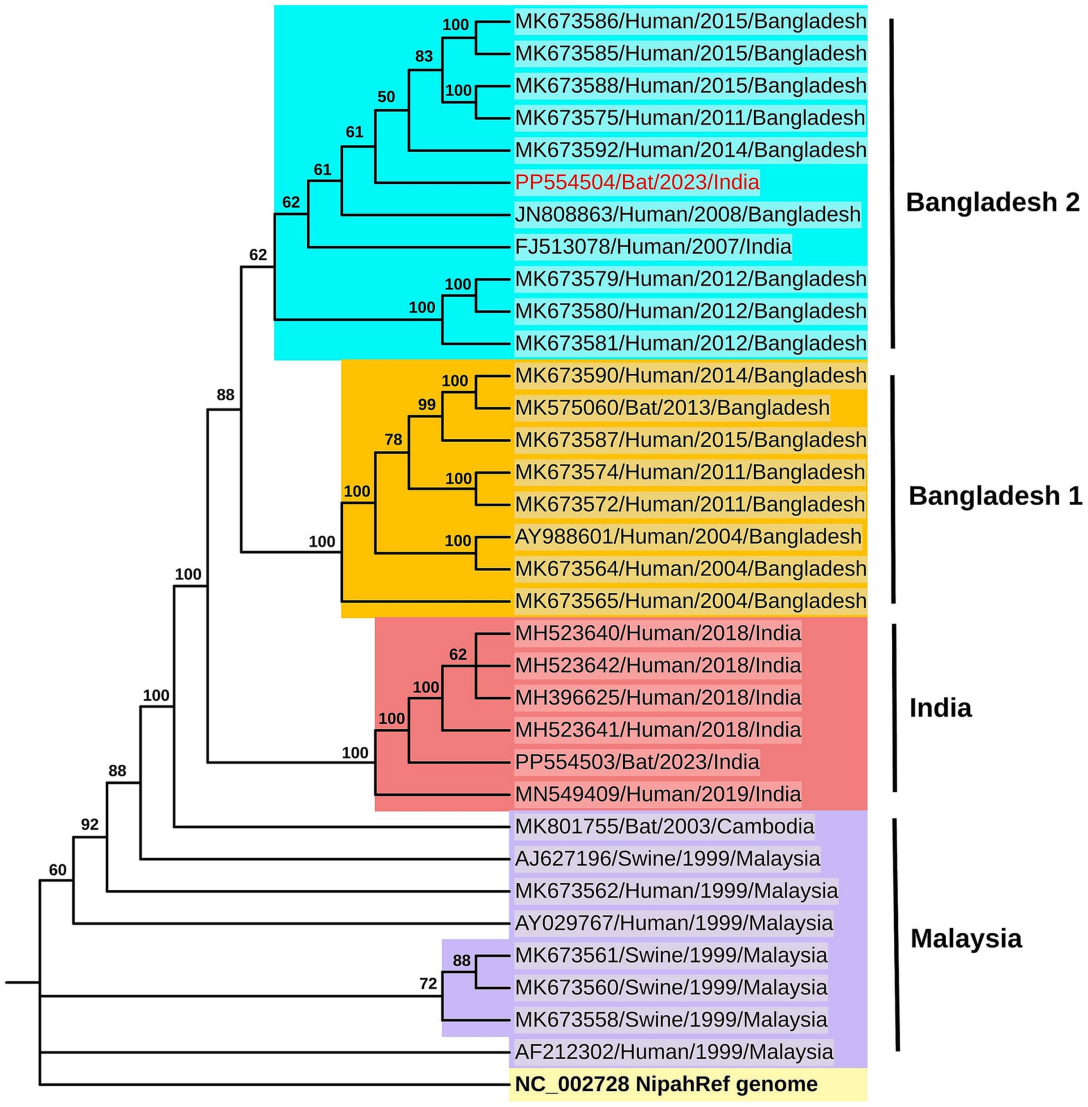
Phylogenetic analysis: The maximum likelihood phylogenetic tree generated using the TN + F model in IQTREE for the genome sequence of the Nipah virus (Accession ID: PP554504) retrieved from the *P. medius* (colored in red) along with the NiV reference sequence (light yellow background) as well as sequences from the NiV-Bangladesh clade-2 (cyan blue background) and clade-1 (dark yellow background), India (red background) and Malaysia (lavender background) clades. The sequences are labeled with GenBank accession number, host, year, and country, and the bootstrap values are marked along the branches.

The phylogenetic analysis showed close homology of the sequences retrieved to the NiV Bangladesh genotype (Figure 3, Supplementary Figure S2). Percent identity ranging from 98.8 to 99.4% was observed with the NiV-Bangladesh-2 clade which comprises of sequences from Bangladesh as well as one sequence obtained from an outbreak in West Bengal, India (Table 3, Figure 3). A percent identity of about ~98% with NiV-Bangladesh clade 1, ~97% with NiV-Indian clade, and ~ 92% with NiV-Malaysian clade was observed (Table 3, Figure 3). The analysis of amino acid differences showed the reported substitutions present in the Bangladesh genotype sequences along with a few additional substitutions in P/V/W (Q247H, S335F, A383V), F (P298H, R300S, F301Y, N302K), and L (Q2087R) protein (Supplementary Table S2).

**Table 3:** The percent identity of the NiV genome retrieved in the study (Accession ID: PP554504) with the Nipah virus reference strain (NC_002728) and a subset of full genome sequences available in the database.

NiV clades	Sequence ID	Percent identity
Bangladesh-2	MK673586_Hu_2015_Bangladesh	99.26
	MK673585_Hu_2015_Bangladesh	99.27
	MK673588_Hu_2015_Bangladesh	99.28
	MK673575_Hu_2011_Bangladesh	99.23
	MK673592_Hu_2014_Bangladesh	99.41
	JN808863_Hu_2008_Bangladesh	99.10
	FJ513078_Hu_2007_India	99.00
	MK673579_Hu_2012_Bangladesh	98.95
	MK673580_Hu_2012_Bangladesh	98.88
	MK673581_Hu_2012_Bangladesh	98.88
Bangladesh-1	MK673590_Hu_2014_Bangladesh	98.69
	MK575060_Bat_2013_Bangladesh	98.49
	MK673587_Hu_2015_Bangladesh	98.79
	MK673574_Hu_2011_Bangladesh	98.82
	MK673572_Hu_2011_Bangladesh	98.51
	AY988601_Hu_2004_Bangladesh	98.60
	MK673564_Hu_2004_Bangladesh	98.69
	MK673565_Hu_2004_Bangladesh	98.79
India	MH523640_Hu_2018_India	97.38
	MH523642_Hu_2018_India	97.48
	MH396625_Hu_2018_India	97.71
	MH523641_Hu_2018_India	97.15
	PP554504_Bat_ 2023_India	97.48
	MN549409_Bat_2019_India	97.72
Malaysia	MK801755_Bat_2003_Cambodia	92.55
	AJ627196_Sw_1999_Malaysia	92.60
	MK673562-Hu 1999_Malaysia	92.75
	AY029767_Hu_1999_Malaysia	92.65
	MK673561_Sw_1999_Malaysia	92.72
	MK673560_Sw_1999_Malaysia	92.72
	MK673558_Sw_1999_Malaysia	92.71
	AF212302_Hu_1999_Malaysia	92.66
	NC_002728_Nipah Reference	92.66

### 3.4 Virus isolation

The virus isolation attempts were unsuccessful in both cell culture and animal models from Nipah virus PCR-positive samples.

### 3.5 Anti-Nipah IgG antibody detection by ELISA and neutralization test

**Figure 4:**
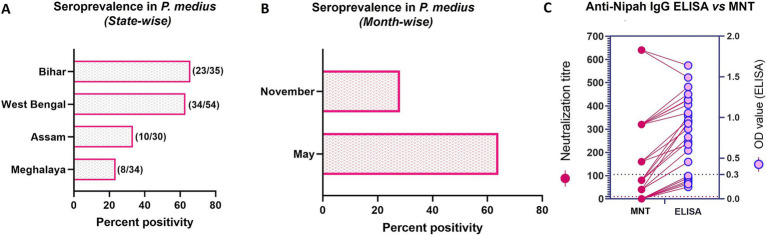
Nipah virus seroprevalence in *P. medius*. **(A)** The horizontal bar graph shows the percent positivity of bats with anti-Nipah IgG antibodies estimated by ELISA in Bihar, West Bengal, Assam, and Meghalaya states (the total number of IgG-positive samples out of the total serum samples tested is marked on the right of each bar). **(B)** The horizontal bar graph shows the percent positivity of bats with anti-Nipah IgG antibodies estimated by ELISA during the November and May months. **(C)** The graph shows the comparison of the neutralization titer (on the left y-axis) with the OD value of ELISA (on the right y-axis) of a subset of bat serum (19 ELISA positive and 6 ELISA negative samples). The cut-off of the microneutralization test (MNT) is marked as dotted lines (dark pink) at 10 on the left y-axis and ELISA as blue dotted lines at 0.3 on the right y-axis. The lines connect the values of the same sample by MNT and ELISA.

The NiV antibody positivity estimated by ELISA in each state was 23.5% (8/34) in Shillong Meghalaya, 33.3% (10/30) in Guwahati, Assam, 62.9% (34/54) in Binnaguri, West Bengal and 65.7% (23/35) in Patna, Bihar (Figure 4A). The seroprevalence was higher in May, at 64% (57/89), compared to November, which had a rate of 28.13% (18/64) (Figure 4B). Out of the 34 seropositive bats from Binnaguri, four were juvenile and out of the 23 positives from Bihar, one was from a juvenile bat. A subset of positive samples (*n* = 19) and negative samples (*n* = 6) was confirmed for neutralizing antibodies by neutralization assay. All the IgG-positive samples tested for neutralizing antibodies were found to have neutralization antibody titer ≥40 (Figure 4C, Supplementary Table S3).

## 4 Discussion

The states of Bihar, West Bengal, Meghalaya, and Assam in India, which are adjacent to Bangladesh, showed a high seroprevalence in *P. medius* during the study. Among these states, West Bengal, Assam, and Meghalaya share a border with Bangladesh. During the sampling period of the study, fourteen cases of NiV infection were reported in several districts of Bangladesh, including Naogaon, Natore, Pabna, Rajshahi, Narsingdi, Rajbari, and Shariatpur. The case fatality rate was 71.4% (Nipah virus infection, n.d.; Gavi, n.d.). While Naogaon and Rajshahi districts share borders with the Indian state of West Bengal, the other districts where cases were reported are also situated closer to India.

The prevalence of NiV in bats in Bihar and Meghalaya states of India has not been studied previously. This study reports the first instance of viral RNA positivity in bats from Bihar state. Additionally, the Meghalaya state, which shares a border with the non-Nipah belt of Bangladesh, showed seroprevalence. A previous study demonstrated the presence of viral RNA and seroprevalence in *P. medius* in districts outside the Nipah belt of Bangladesh (Epstein et al., 2020). The sampling site in Meghalaya is only about 75 km far from Sylhet in Bangladesh which has reported NiV bat positivity (Anderson et al., 2019). Previous studies has also reported NiV detection in bats from the North Bengal region of India in Myanaguri in 2010 and Cooch Behar in 2015 (Yadav et al., 2012, 2018). These areas are located close to the site that was sampled for the present study in West Bengal, where NiV RNA was also detected. In 2015, the Dhubri district, located in the western part of Assam near the Nipah belt of Bangladesh, was surveyed, and viral RNA was detected in *P. medius* (Yadav et al., 2018). For the current study, a new urban location in central Assam, far from the previous surveyed site was chosen. This site is about 140 km from Sylhet, Bangladesh, and approximately 170 km from Dhubri, India where NiV positivity in bats was reported earlier. It was found that bats in this region have seropositivity.

Higher seroprevalence was observed in the West Bengal and Bihar sites, which were sampled during May, compared to the sites in Assam and Meghalaya, which were sampled in November. The higher prevalence of antibodies against NiV in bats, including juveniles, and the virus detection indicate that the virus is actively circulating in this area. Fluctuating seroprevalence (30 to 80%) and low NiV prevalence in *P. medius* have been reported in a longitudinal study performed at Faridpur, Bangladesh. There was also no evidence of seasonal fluctuations in seroprevalence or virus detection in this longitudinal study (Epstein et al., 2020). A three-year longitudinal study conducted on *P. hypomelanus* bats in Malaysia also showed a non-seasonal fluctuating seroprevalence ranging from 1 to 20% (Rahman et al., 2013). Our earlier studies in the northeast region of India in 2009–10 (May to October in Myanaguri) and 2015 (March–May in Jalpaiguri, Cooch Behar, and Dhubri; November in Dhubri) have shown low seropositivity (Yadav et al., 2012, 2018). Studies in Kerala state during the outbreak and non-outbreak periods have also shown seropositivity ranging between 9 to 21% in bats (Sudeep et al., 2021; Gokhale et al., 2022; Yadav et al., 2022; Balasubramanian et al., 2024).

The current study confirms active Nipah virus circulation among bats in eastern and northeastern India, with all surveyed sites showing seropositivity and viral RNA detected in Bihar and West Bengal. The absence of active human surveillance in India might have been one of the reasons for the absence of Nipah virus case detection from these studied regions. The hospital-based national surveillance system which includes active and passive surveillance strategies established since 2006 has played a key role in the detection and response of the outbreaks in Bangladesh (Satter et al., 2023). With the repeated outbreaks in Kerala state of India, syndromic surveillance was initiated in the state and this has resulted in early detection of cases and containment (Yadav et al., 2022). All these points towards the necessity of strengthening public health surveillance in the region. Recently, ICMR has included the Nipah virus in the list of pathogens for respiratory and acute encephalitis surveillance through its virus disease research network laboratories and associated tertiary care hospitals in West Bengal and Kerala (Unpublished data). This approach should be expanded to other regions of the country.

The throat and rectal swab samples collected during the present study tested negative for NiV, suggesting the absence of viral shedding through the gastrointestinal tract. The absence of swab positivity in organ-positive bats suggests that viral excretion in bats is episodic. Organ viral positivity in *Pteropus* bats has been reported in field and experimental studies (Middleton et al., 2007; Yadav et al., 2018). During experimental infection in *P. poliocephalus* bats, a transient presence of NiV was found in the kidney and uterus (Middleton et al., 2007). Multiple field studies have reported NiV RNA detection in spleen and kidney samples of *P. medius* (Epstein et al., 2016; Yadav et al., 2018; Balasubramanian et al., 2024). Urine and urogenital swabs, which are important samples to understand virus shedding, were not collected in the study. Efforts to isolate the virus were unsuccessful. Based on previous reports and our observations, virus isolation is challenging, and successful virus isolation does not appear to correlate with virus copy numbers (Anderson et al., 2019; Yadav et al., 2019; Balasubramanian et al., 2024).

The NiV strains found in Bangladesh have a nucleotide difference of around 9% from the Malaysian genotype and 3% from the virus sequences found in South India. The genome obtained from Bihar in the present study is over 99% similar to the NiV sequence of the 2007 outbreak from West Bengal in India, suggesting that a persistent virus strain is circulating in the region. The evolutionary rate estimated for NiV is 2.18 × 10^−4^ substitutions/site/year, which is relatively slow compared to similar viruses (Whitmer et al., 2021). It has been reported that there is a high level of sequence identity among NiV isolates found in bats in Bangladesh (Epstein et al., 2016; Anderson et al., 2019). The amino acid differences in the sequence retrieved in the present study were almost similar to those reported earlier for NiV-B sequences from Bangladesh and India (Lo et al., 2012; Mohandas et al., 2023). However, the significance of many of these changes is yet to be explored.

Fruit bats play a critical role in the ecosystem. The foraging range of *Pteropus* bats ranges from a few kilometers to 20–30 km and occasional reports of long-distance movement have also been reported. Migrant bats typically have a metapopulation structure with distinct roosts connected by migration, potentially exposing resident and migrant populations to viruses (Fleming, 2010). Migrations associated with breeding season, food scarcity, different seasons, and environmental disturbances have been reported (Epstein et al., 2020). Many studies have correlated spillover events with changing bat ecology associated with land use change, habitat loss, and periodic food resource shortages (McKee et al., 2021; Eby et al., 2023). Deforestation and urbanization have decreased forest cover and natural foraging resources for bats (McKee et al., 2021; Roy et al., 2024). As a result, bats are being forced to move and search for food in cultivated areas such as farms and home gardens. This could potentially increase the risk of disease spillover and such opportunistic feeding behavior has been documented in Malaysia and Bangladesh during previous outbreaks (Chua et al., 1999; McKee et al., 2021). Hence, it is crucial to prioritize the conservation of bat roosting trees, tree cover, and foraging sites to reduce human-bat interaction events. *P. medius* are seen throughout the Indian subcontinent (Saikia, 2018). The studies on bat ecology, behavior, foraging resources, migration, and land-use changes in the region could enhance the understanding of spillover threats. Increasing community awareness of potential risk factors and strategies to minimize exposure is essential.

The findings of this study indicate that a larger geographical area in India is at risk for Nipah virus spillover. These findings highlight the necessity of enhancing human surveillance for Nipah virus infection and investigating the disease risk factors in these regions of India. The implementation of a One Health approach is critical in preventing virus spillovers.

## Data Availability

The datasets presented in this study can be found in online repositories. The names of the repository/repositories and accession number(s) can be found in the article/Supplementary material.
