# Supplementary material for: Nipah virus survey in Pteropus medius of eastern and northeastern region of India, 2022–2023
Source: Front Microbiol. 2024 Dec 24;15:1493428. doi: 10.3389/fmicb.2024.1493428 (PMC11703920; doi:10.3389/fmicb.2024.1493428)
Supplement: Supplementary file 1 [file Data_Sheet_1.PDF]

```
NC_002728_Nipah_Reference        ACCAAACAAGGGAGAATATGGATACGTTAAAATATATAACGTATTTTTAAAACTTAGGAA     60
PP554504_Bat_2023_India          ----------------------------------------------------------AA     2
JN808863_Hu_2008_Bangladesh      ACCAAACAAGGGAAAATATGGATACGTTAAAATATATAACGTATTTCTAAAACTTAGGAA     60
                                                                                           **

NC_002728_Nipah_Reference        CCAAGACAAACACTTTTGGTCTTGGTATTGGATCCTCAAGAAATATATCATCATGAGTGA     120
PP554504_Bat_2023_India          CCAAGACAAACACTTTTGGTCTTGGTATTGGATCCTCAAGAAATATATCATCATGAGTGA     62
JN808863_Hu_2008_Bangladesh      CCAAGACAAACACTTTTGGTCTTGGTATTGGATCCTCAAGAAATATATCATCATGAGTGA     120
                                 ************************************************************

NC_002728_Nipah_Reference        TATCTTTGAAGAGGCGGCTAGTTTTAGGAGTTATCAATCTAAGTTAGGGAGAGATGGGAG     180
PP554504_Bat_2023_India          TATTTTTGAAGAGGCGGCTAGTTTTAGGAGNNNNNNNNNTAAGTTAGGAAGAGATGGGAG     122
JN808863_Hu_2008_Bangladesh      TATTTTTGAAGAGGCGGCTAGTTTTAGGAGTTATCAATCTAAGTTAGGAAGAGATGGGAG     180
                                 *** **************************         ********* ***********

NC_002728_Nipah_Reference        GGCTAGTGCAGCAACTGCTACTTTGACAACCAAGATAAGGATATTTGTACCAGCTACTAA     240
PP554504_Bat_2023_India          GGCTAGTGCGGCAACTGCTACTTTGACAACCAAGATAAGGATATTTGTACCGGCTACCAA     182
JN808863_Hu_2008_Bangladesh      GGCTAGTGCGGCAACTGCTACTTTGACAACCAAGATAAGGATATTTGTACCGGCTACTAA     240
                                 ********* ***************************************** ***** **

NC_002728_Nipah_Reference        TAGTCCAGAGCTCAGATGGGAACTAACATTGTTTGCACTTGATGTGATTAGATCTCCGAG     300
PP554504_Bat_2023_India          TAGTCCAGAGCTCAGATGGGAACTAACATTATTTGCACTTGATGTGATCAGATCTCCGAG     242
JN808863_Hu_2008_Bangladesh      TAGTCCAGAGCTCAGATGGGAACTAACATTATTTGCACTTGATGTGATCAGATCTCCGAG     300
                                 ****************************** ***************** ***********

NC_002728_Nipah_Reference        TGCTGCCGAGTCAATGAAAGTTGGAGCTGCTTTCACACTCATCTCTATGTATTCAGAGAG     360
PP554504_Bat_2023_India          TGCAGCCGAGTCAATGAAAGTTGGAGCTGCTTTTACACTCATCTCTATGTATTCAGAGAG     302
JN808863_Hu_2008_Bangladesh      TGCAGCCGAGTCAATGAAAGTTGGAGCTGCTTTTACACTCATCTCTATGTATTCAGAGAG     360
                                 *** ***************************** **************************

NC_002728_Nipah_Reference        ACCCGGGGCTCTCATTAGAAGTCTCCTCAATGACCCAGACATTGAAGCTGTAATAATAGA     420
PP554504_Bat_2023_India          ACCCGGGGCCCTCATTAGAAGCCTCCTCAATGACCCAGACATTGAAGCTGTAATAATAGA     362
JN808863_Hu_2008_Bangladesh      ACCCGGGGCCCTCATTAGAAGCCTCCTCAATGACCCAGACATTGAAGCTGTAATAATAGA     420
                                 ********* *********** **************************************

NC_002728_Nipah_Reference        TGTTGGATCAATGGTCAACGGAATACCAGTAATGGAGAGGAGAGGAGACAAGGCTCAGGA     480
PP554504_Bat_2023_India          TGTCGGATCAATGGTCAACGGGATACCAGTGATGGAAAGGAGGGGAGACAAAGCTCAAGA     422
JN808863_Hu_2008_Bangladesh      TGTTGGATCAATGGTCAACGGGATACCAGTGATGGAAAGGAGGGGAGACAAAGCTCAAGA     480
                                 *** ***************** ******** ***** ***** ******** ***** **

NC_002728_Nipah_Reference        GGAGATGGAAGGCTTGATGAGAATCCTCAAAACTGCTCGAGACAGCAGCAAGGGAAAAAC     540
PP554504_Bat_2023_India          GGAGATGGAAGGCTTGATGAGAATCCTCAAGACTGCTCGGGACAGCAGCAAGGGAAAGAC     482
JN808863_Hu_2008_Bangladesh      GGAGATGGAAGGCTTGATGAGAATCCTCAAGACTGCTCGGGACAGCAGCAAGGGAAAGAC     540
                                 ****************************** ******** ***************** **

NC_002728_Nipah_Reference        ACCTTTTGTTGACAGCCGAGCTTACGGCCTACGGATAACAGACATGAGCACCCTGGTCTC     600
PP554504_Bat_2023_India          ACCTTTTGTTGATAGCCGAGCTTATGGCCTACGGATAACAGACATGAGCACTCTGGTCTC     542
JN808863_Hu_2008_Bangladesh      ACCTTTTGTTGATAGCCGAGCTTATGGCCTACGGATAACAGACATGAGCACTCTGGTCTC     600
                                 ************ *********** ************************** ********

NC_002728_Nipah_Reference        TGCAGTTATCACCATCGAGGCCCAGATCTGGATACTGATCGCTAAAGCAGTTACAGCTCC     660
PP554504_Bat_2023_India          TGCAGTTATCACCATCGAGGCCCAAATCTGGATACTGATTGCCAAAGCAGTTACAGCTCC     602
JN808863_Hu_2008_Bangladesh      TGCAGTTATCACCATCGAGGCCCAAATCTGGATACTGATTGCCAAAGCAGTTACAGCTCC     660
                                 ************************ ************** ** *****************

NC_002728_Nipah_Reference        CGACACTGCCGAGGAAAGTGAAACTAGAAGATGGGCTAAATACGTCCAACAAAAGAGAGT     720
PP554504_Bat_2023_India          AGACACTGCCGAGGAGAGTGAAACTAGAAGATGGGCTAAGTACGTTCAACAAAAGAGAGT     662
JN808863_Hu_2008_Bangladesh      AGACACTGCCGAGGAGAGTGAAACTAGAAGATGGGCTAAGTACGTTCAACAAAAGAGAGT     720
                                  ************** *********************** ***** **************

NC_002728_Nipah_Reference        CAATCCGTTCTTTGCTCTAACTCAGCAATGGCTAACAGAAATGAGGAATCTGCTCTCCCA     780
PP554504_Bat_2023_India          CAATCCGTTCTTTGCTCTAACTCAGCAATGGCTAACAGAGATGAGAAATCTGCTTTCTCA     722
JN808863_Hu_2008_Bangladesh      CAATCCGTTCTTTGCTCTAACTCAGCAATGGCTAACAGAGATGAGGAATCTGCTTTCTCA     780
                                 *************************************** ***** ******** ** **

NC_002728_Nipah_Reference        GAGTCTATCAGTAAGGAAGTTCATGGTTGAGATCCTCATAGAAGTCAAGAAAGGAGGATC     840
PP554504_Bat_2023_India          GAGTCTGTCAGTAAGAAAGTTTATGGTTGAGATCCTCATCGAAGTCAAGAAAGGAGGATC     782
JN808863_Hu_2008_Bangladesh      GAGTCTGTCAGTAAGAAAGTTTATGGTTGAGATCCTCATCGAAGTCAAGAAAGGAGGATC     840
                                 ****** ******** ***** ***************** ********************

NC_002728_Nipah_Reference        TGCTAAAGGCAGAGCAGTAGAAATAATCTCAGACATCGGAAACTATGTCGAGGAAACTGG     900
PP554504_Bat_2023_India          TGCTAAAGGCAGAGCAGTGGAAATAATCTCAGACATTGGAAACTATGTCGAGGAAACTGG     842
JN808863_Hu_2008_Bangladesh      TGCTAAAGGCAGAGCAGTGGAAATAATCTCAGACATTGGAAACTATGTCGAGGAAACTGG     900
                                 ****************** ***************** ***********************

NC_002728_Nipah_Reference        TATGGCAGGATTCTTCGCAACCATCAGATTCGGGTTGGAGACAAGGTATCCAGCACTTGC     960
PP554504_Bat_2023_India          CATGGCAGGATTCTTCGCAACCATCAGATTTGGGTTGGAGACAAGGTATCCAGCACTTGC     902
JN808863_Hu_2008_Bangladesh      CATGGCAGGATTCTTCGCAACCATCAGATTTGGGTTGGAGACAAGGTATCCAGCACTTGC     960
                                  ***************************** *****************************

NC_002728_Nipah_Reference        ACTCAACGAATTCCAGAGTGACCTCAACACCATCAAAAGCTTGATGCTACTCTACAGAGA     1020
PP554504_Bat_2023_India          ACTCAATGAATTCCAGAGTGACCTCAACACCATCAAAAGCTTGATGCTACTCTACAGAGA     962
JN808863_Hu_2008_Bangladesh      ACTCAATGAATTCCAGAGTGACCTCAACACCATCAAAAGCTTGATGCTACTCTACAGAGA     1020
                                 ****** *****************************************************

NC_002728_Nipah_Reference        AATTGGCCCAAGAGCCCCTTATATGGTGCTTCTTGAAGAATCAATTCAGACTAAATTTGC     1080
PP554504_Bat_2023_India          AATTGGCCCAAGAGCCCCTTATATGGTGCTTCTTGAAGAATCAATTCAGACTAAGTTTGC     1022
JN808863_Hu_2008_Bangladesh      AATTGGCCCAAGAGCCCCTTATATGGTGCTTCTTGAAGAATCAATTCAGACTAAGTTTGC     1080
```

```
                                  ****************************************************** *****

NC_002728_Nipah_Reference         CCCTGGAGGTTACCCATTATTGTGGAGCTTTGCCATGGGTGTGGCTACTACTATTGACAG      1140
PP554504_Bat_2023_India           CCCTGGAGGTTACCCGCTATTGTGGAGCTTTGCTATGGGTGTGGCTACTACTATTGACAG      1082
JN808863_Hu_2008_Bangladesh       CCCTGGAGGTTACCCGCTATTGTGGAGCTTTGCTATGGGTGTGGCTACTACTATTGACAG      1140
                                  ***************  **************** **************************

NC_002728_Nipah_Reference         GTCTATGGGGGCATTGAATATCAATCGTGGTTATCTTGAGCCTATGTATTTCAGACTAGG      1200
PP554504_Bat_2023_India           GTCTATGGGGGCATTAAACATCAATCGTGGTTATCTTGAACCTATGTATTTCAGACTAGG      1142
JN808863_Hu_2008_Bangladesh       GTCTATGGGGGCATTAAACATCAATCGTGGTTATCTTGAACCTATGTATTTCAGACTAGG      1200
                                  *************** ** ******************** ********************

NC_002728_Nipah_Reference         CCAAAAATCAGCACGTCACCATGCTGGAGGAATTGATCAGAACATGGCAAATAGACTGGG      1260
PP554504_Bat_2023_India           CCAAAAATCAGCACGTCACCATGCTGGAGGAATTGATCAGAACATGGCGAATAGACTAGG      1202
JN808863_Hu_2008_Bangladesh       CCAAAAATCAGCACGTCACCATGCTGGAGGAATTGATCAGAACATGGCGAATAGACTAGG      1260
                                  ************************************************ ******** **

NC_002728_Nipah_Reference         ACTAAGTTCAGATCAAGTTGCAGAACTCGCTGCTGCAGTTCAGGAAACATCAGCAGGAAG      1320
PP554504_Bat_2023_India           ATTAAGTTCAAATCAAGTTGCAGAACTCGCTGCCGCAGTTCAAGAAACATCAGCAGGAAG      1262
JN808863_Hu_2008_Bangladesh       ATTAAGTTCAAATCAAGTTGCAGAACTCGCTGCCGCAGTTCAAGAAACATCAGCAGGAAG      1320
                                  * ******** ********************** ******** *****************

NC_002728_Nipah_Reference         GCAAGAGAGTAATGTTCAGGCTAGAGAGGCAAAATTTGCTGCAGGAGGTGTGCTCATTGG      1380
PP554504_Bat_2023_India           GCAAGAGAGTAACGTTCAGGCCAGAGAAGCTAAATTTGCTGCAGGAGGTGTGCTCATCGG      1322
JN808863_Hu_2008_Bangladesh       GCAAGAGAGTAACGTTCAGGCCAGAGAAGCTAAATTTGCTGCAGGAGGTGTGCTCATCGG      1380
                                  ************ ******** ***** ** ************************** **

NC_002728_Nipah_Reference         AGGCAGTGATCAAGATATCGATGAAGGGGAAGAACCTATAGAACAGAGTGGCAGACAGTC      1440
PP554504_Bat_2023_India           GGGCAGTGATCAAGATGTCGACGAAGAGGAAGAACCTATAGAACAGAGTGGCCGACAGTC      1382
JN808863_Hu_2008_Bangladesh       GGGCAGTGATCAAGATGTCGACGAAGAGGAAGAACCTATAGAGCAGAGTGGCCGACAGTC      1440
                                   *************** **** **** *************** ********* *******

NC_002728_Nipah_Reference         AGTTACCTTCAAAAGGGAGATGAGTATTTCATCCCTTGCTAACAGTGTGCCGAGCAGTTC      1500
PP554504_Bat_2023_India           AGTTACTTTCAAAAGGGAAATGAGTATTTCATCTCTTGCTGACAGTGTGCCGAGCAGTTC      1442
JN808863_Hu_2008_Bangladesh       AGTTACTTTCAAAAGGGAAATGAGTATTTCATCTCTTGCTGACAGTGTGCCGAGCAGTTC      1500
                                  ****** *********** ************** ****** *******************

NC_002728_Nipah_Reference         TGTGAGCACATCCGGTGGGACCAGATTGACTAATTCATTACTAAACCTCAGATCAAGACT      1560
PP554504_Bat_2023_India           TGTGAGCACCTCAGGTGGGACCAGATTGACTAATTCTTTGCTAAACCTCAGATCAAGGCT      1502
JN808863_Hu_2008_Bangladesh       TGTGAGCACCTCAGGTGGGACCAGATTGACTAATTCTTTGCTAAACCTCAGATCAAGGCT      1560
                                  ********* ** *********************** ** ***************** **

NC_002728_Nipah_Reference         GGCTGCAAAAGCAGCAAAAGAAGCCGCCTCATCCAATGCAACAGATGATCCAGCAATCAG      1620
PP554504_Bat_2023_India           GGCTGCTAAAGCAGCAAAGGAAGCCGCTTCGTCTAATGCAACAGATGATCCAGCAATCAG      1562
JN808863_Hu_2008_Bangladesh       GGCTGCTAAAGCAGCAAAGGAAGCCGCTTCGTCTAATGCAACAGATGATCCAGCAATCAG      1620
                                  ****** *********** ******** ** ** **************************

NC_002728_Nipah_Reference         CAACAGAACTCAAGGGGAATCAGAGAAGAAGAATAATCAAGACCTCAAACCTGCTCAAAA      1680
PP554504_Bat_2023_India           CAACAAAGATCAAAGGGAATCAGAAAAGAAGAATAACCAAGATCTCAAACCCACTCAAAA      1622
JN808863_Hu_2008_Bangladesh       CAACAAAGATCAAAGGGAATCAGAGAAGAAGAATAACCAAGATCTCAAACCCACTCAAAA      1680
                                  ***** *  **** ********** *********** ***** ********  *******

NC_002728_Nipah_Reference         TGACCTTGATTTCGTCAGAGCTGATGTGTGACGTCTATTTCCAATATTCTACAGTATCCA      1740
PP554504_Bat_2023_India           TGATCTTGACTTTGTCAGAGCTGATGTGTGACGTTTATTTTCAATATTCTACAATATCCA      1682
JN808863_Hu_2008_Bangladesh       TGATCTTGACTTTGTCAGAGCTGATGTGTGACGTTTATTTTCAATATTCTACAATATCCA      1740
                                  *** ***** ** ********************* ***** ************ ******

NC_002728_Nipah_Reference         AAAATCTTTCTATAGTACACTATCATAATACGACACTAAGGGATCAACCATATCAAAGTT      1800
PP554504_Bat_2023_India           AAAATCTTTTTATAATACGCTATCATAATACAACACTAAGGGACCAATTACATTAAAGTT      1742
JN808863_Hu_2008_Bangladesh       AAAATCTTTTTATAATACACTATCATAATACAACACTAAGGGACCAATTACATTAAAGTT      1800
                                  ********* **** *** ************ *********** ***  * ** ******

NC_002728_Nipah_Reference         ACGAATCGTTTTAATTATATTAATCAAATGATACTCTTTTATGGGCAAACCGAAGAACCA      1860
PP554504_Bat_2023_India           GCGAATCGTTTTAACTGTATTAATCAAGTGATACTCTTTTATGGGCAAAGCGAACAACCA      1802
JN808863_Hu_2008_Bangladesh       GCGAATCGTTTTAACTGTATTAATCAAGTGATACTCTTTTATGGGCAAAGCGAACAACCA      1860
                                   ************* * ********** ********************* **** *****

NC_002728_Nipah_Reference         ATGTCTACATGTAAATTGAGCTTTGGTATTGCAATCTAATACTTGCTCAAAATCTTGAAC      1920
PP554504_Bat_2023_India           ATGTCTACATGTAAATTAAGCTCTGGTATTGCAATCTAATATTTACTCAAAATCTTGAGC      1862
JN808863_Hu_2008_Bangladesh       ATGTCTACATGTAAATTAAGCTCTGGTATTGCAATCTAATATTTACTCAAAATCTTGAGC      1920
                                  ***************** **** ****************** ** ************* *

NC_002728_Nipah_Reference         TATTAGTGTAATTTCTATCATCATAGAGTTATCAAGATTTTATTATATAAGTTGGTGCAG      1980
PP554504_Bat_2023_India           TATTAGTGCAATTCATACCACCATAGATTTATCAAGATTTTATTATATAAGTTGGTGCAG      1922
JN808863_Hu_2008_Bangladesh       TATTAGTGCAATTCATACCACCATAGATTTATCAAGATTTTATTATATAAGTTGGTGCAG      1980
                                  ******** ****  ** ** ****** ********************************

NC_002728_Nipah_Reference         ATCTTTGGACATGAATTACACACTACACTCTAATGAAGACAAAATTTACATTACATATTT      2040
PP554504_Bat_2023_India           GTCTTTGGACATGAATTACACACTACACCCTAATAAAGACAAAATCTATATTACATATTT      1982
JN808863_Hu_2008_Bangladesh       GTCTTTGGACATGAATTACACACTACACCCTAATAAAGATAAAATCTATATTACATATTT      2040
                                   *************************** ***** **** ***** ** ***********

NC_002728_Nipah_Reference         AAGGACTATTTCCTATCCTTTCAATGGTACTTGGTTATGAAGGTTTCTTAATTTAACTAA      2100
PP554504_Bat_2023_India           AAAGACTATATCCTATCCTTTCAATGGTGCTTGGTTACGAAGGTTTCTCAATTTAAATAA      2042
JN808863_Hu_2008_Bangladesh       AAAGACTATATCCTATCCTTTCAATGGTGCTTGGTTACGAAGGTTTCTCAATTTAAATAA      2100
                                  ** ****** ****************** ******** ********** ******* ***

NC_002728_Nipah_Reference         GCTACTGTCTTTGCACTGGAATATACAATACCTCTTACCTCATTTCTTACTTTAATATCA      2160
```

```
PP554504_Bat_2023_India         GCTATTGTCTTTGCACTGGAGTATACAATACCTCTTACCTCATTTCTTACTTCAATATCA    2102
JN808863_Hu_2008_Bangladesh     GCTATTGTCTTTGCACTGGAGTATACAATACCTCTTACCTCATTTCTTACTTCAATATCA    2160
                                **** *************** ******************************* *******

NC_002728_Nipah_Reference       TGTTATTTTTTTGATAAGTCACTTAACTTGACCAAGGTCTACCAGGTAATGCTCGCACAA    2220
PP554504_Bat_2023_India         TGTTATTTTTTTGATAAGTCACTTAATTTGACTAAGATCTACTAGGTAATGCTCACATAA    2162
JN808863_Hu_2008_Bangladesh     TGTTATTTTTTTGATAAGTCACTTAATTTGACTAAGATCTACTAGGTAATGCTCACATAA    2220
                                ************************** ***** *** ***** *********** ** **

NC_002728_Nipah_Reference       GTGAACTGCAATCTCAACTTAGATTAAACATAATCATGCAAAATCACTATTTTGTACTAC    2280
PP554504_Bat_2023_India         ATGAATTGCAATCTTAACTTAGATTAAACATAATCATGCAAAGTCATTATTTTACACTAC    2222
JN808863_Hu_2008_Bangladesh     ATGAATTGCAATCTTAACTTAGATTAAACATAATCATGCAAAGTCATTATTTTACACTAC    2280
                                 **** ******** *************************** *** ******  *****

NC_002728_Nipah_Reference       TAACTCATTAAGAAAAACTTAGGATCCAAGAGATTTACTCTAGGATCTCCTATTAAGCTT    2340
PP554504_Bat_2023_India         TAACTCATTAAGAAAAACTTAGGATCCAAGAGATTTACTCTAGGATCTCCTATCAAATTT    2282
JN808863_Hu_2008_Bangladesh     TAACTCATTAAGAAAAATTTAGGATCCAAGAGATTTACTCTAGGATCTCCTATCAAATTT    2340
                                ***************** *********************************** **  **

NC_002728_Nipah_Reference       AGCAGTCATTAGTTGAGAGTTCAACTTGCAAAACTCTAACCTTCACTCTAATAACAATTC    2400
PP554504_Bat_2023_India         AGCAGTTATCAGCTGGGAGTTCAACTTACAAAACTCTAACCTTCACTTTAATAACAATTC    2342
JN808863_Hu_2008_Bangladesh     AGCAGTTATCAGCTGGGAGTTCAACTTACAAAACTCTAACCTTCACTTTAATAACAATTC    2400
                                ****** ** ** ** *********** ******************* ************

NC_002728_Nipah_Reference       ATCCAATGGATAAATTGGAACTAGTCAATGATGGCCTCAATATTATTGACTTTATTCAGA    2460
PP554504_Bat_2023_India         ATTCAATGGATAAATTGGAACTAGTTAATGATGGCCTCAATATTATTGACTTTATTCAGA    2402
JN808863_Hu_2008_Bangladesh     ATTCAATGGATAAATTGGAACTAGTTAATGATGGCCTCAATATTATTGACTTTATTCAGA    2460
                                ** ********************** **********************************

NC_002728_Nipah_Reference       AGAACCAAAAAGAAATACAGAAGACATACGGACGATCAAGTATTCAACAACCCAGCATCA    2520
PP554504_Bat_2023_India         AGAACCAAAAAGAAATACAGAAGACATACGGACGATCAAGCATTCAACAACCCAGCATCA    2462
JN808863_Hu_2008_Bangladesh     AGAACCAAAAAGAAATACAGAAGACATACGGACGATCAAGCATTCAACAACCCAGCATCA    2520
                                **************************************** *******************

NC_002728_Nipah_Reference       AAGATCAAACAAAAGCCTGGGAAGATTTTCTGCAGTGCACCAGTGGAGAATCTGAACAAG    2580
PP554504_Bat_2023_India         AAGACCGAACAAAAGCATGGGAGGATTTTCTGCAGTGCACCAGTGGAGAATCTGAACAAG    2522
JN808863_Hu_2008_Bangladesh     AAGACCGAACAAAAGCATGGGAGGATTTTCTGCAGTGCACCAGTGGAGAATCTGAACAAG    2580
                                **** * ********* ***** *************************************

NC_002728_Nipah_Reference       TTGAGGGGGAATGTCTAAGGATGATGGAGATGTTGAAAGAAGAAACTTGGAGGATCTAT    2640
PP554504_Bat_2023_India         TTGAGGGGGAATGCCTAAGGATGATGGAGGTGTTGAAAGAAGAAGCTTGGAGGATCTAT    2582
JN808863_Hu_2008_Bangladesh     TTGAGGGGGAATGCCTAAGGATGATGGAGGTGTTGAAAGAAGAAGCTTGGAGGATCTAT    2640
                                ************* *************** ************** **************

NC_002728_Nipah_Reference       CCAGTACTTCTCCCACAGATGGAACTATTGGAAAGAGAGTGTCGAACACCCGTGACTGGG    2700
PP554504_Bat_2023_India         CCAGTACTTCTCCCACAGATGGAACTATTGGAAAAAGAGTGTCGAACACCCGTGACTGGG    2642
JN808863_Hu_2008_Bangladesh     CCAGTACTTCTCCCACAGATGGAACTATTGGAAAAAGAGTGTCGAACACCCGTGACTGGG    2700
                                ********************************** *************************

NC_002728_Nipah_Reference       CAGAAGGTTCAGATGACATACAACTGGACCCAGTGGTTACAGACGTTGTATACCATGATC    2760
PP554504_Bat_2023_India         CAGAAGGTTCAGATGACATACAACTGGACCCAGTGGTTACAGACGTTGTATACCATGATC    2702
JN808863_Hu_2008_Bangladesh     CAGAAGGTTCAGATGACATACAACTGGACCCAGTGGTTACAGACGTTGTATACCATGATC    2760
                                ************************************************************

NC_002728_Nipah_Reference       ATGGAGGAGAATGTACCGGATATGGATTTACTTCAAGCCCTGAGAGAGGGTGGAGTGATT    2820
PP554504_Bat_2023_India         ATGGAGGAGAATGTACCGGATATGGATTTACTTCAAGCCCTGAGAGAGGGTGGAGTGATC    2762
JN808863_Hu_2008_Bangladesh     ATGGAGGAGAATGTACCGGATATGGATTTACTTCAAGCCCTGAGAGAGGGTGGAGTGATC    2820
                                ***********************************************************

NC_002728_Nipah_Reference       ACACATCAGGAGCAAACAATGGGAATGTATGTCTTGTATCTGATGCAAAGATGCTGTCCT    2880
PP554504_Bat_2023_India         ACTCATCAGGAGCAAACAATGGGGATGTATGTCTTGTATCTGATGCAAAGGTGCTGTCCT    2822
JN808863_Hu_2008_Bangladesh     ACTCATCAGGAGCAAACAATGGGGATGTATGTCTTGTATCTGATGCAAAGGTGCTGTCCT    2880
                                ** ******************** ************************** *********

NC_002728_Nipah_Reference       ATGCTCCCGAAATTGCAGTTTCTAAAGAAGATCGGGAAACTGATCTAGTTCATCTTGAGA    2940
PP554504_Bat_2023_India         ATGCTCCCGAAATTGCAGTTTCTAAAGAAGATCGGGAAACTGATCTAGTTCACCTTGAGG    2882
JN808863_Hu_2008_Bangladesh     ATGCTCCCGAAATTGCAGTTTCTAAAGAAGATCGGGAAACTGATCTAGTTCACCTTGAGG    2940
                                **************************************************** ******

NC_002728_Nipah_Reference       ATAAACTATCTACTACAGGACTGAATCCCACAGCAGTACCGTTCACTCTGAGAAACCTGT    3000
PP554504_Bat_2023_India         ACAAACTATCTGCTACAGGACTGAACCCTACAGCAATACCATTCACTCCAAAAAATCTGT    2942
JN808863_Hu_2008_Bangladesh     ACAAACTATCTGCTACAGGACTGAACCCTACAGCAATACCATTCACTCCAAAAAATCTGT    3000
                                * ********* ************* ** ****** **** *******  * *** ****

NC_002728_Nipah_Reference       CTGATCCTGCAAAAGACTCTCCTGTGATTGCTGAACACTACTACGGACTAGGAGTTAAAG    3060
PP554504_Bat_2023_India         CTGTTCCTGCAAAAGATTCTCCTGTGATTGCTGAACACTACTACGGACTAGGAGTTAGAG    3002
JN808863_Hu_2008_Bangladesh     CTGTTCCTGCAAAAGATTCTCCTGTGATTGCTGAACACTACTATGGACTAGGAGTTAGAG    3060
                                *** ************ ************************** ************* **

NC_002728_Nipah_Reference       AGCAAAACGTTGGCCCTCAGACTAGCAGAAATGTCAATTTGGACAGCATCAAATTGTACA    3120
PP554504_Bat_2023_India         AGCAAAACGTTGATCCCCAGACTAACAAAAATGTCAATTTGGACAGCATCAAATTGTACA    3062
JN808863_Hu_2008_Bangladesh     AGCAAAACGTTGATCCCCAGACTAACAAAAATGTCAATTTGGACAGCATCAAATTGTACA    3120
                                ************  ** ******* ** ********************************

NC_002728_Nipah_Reference       CATCAGATGACGAAGAGGCAGATCAGCTTGAATTCGAAGATGAGTTTGCAGGAAGCTCAA    3180
PP554504_Bat_2023_India         CATCAGATGACGAAGAGGCAGATCATCTTGAATTTGAGGATGAGTTTGCAGGGAGTTCAA    3122
JN808863_Hu_2008_Bangladesh     CATCAGATGACGAAGAGGCAGATCAGCTTGAATTTGAGGATGAGTTTGCAGGGAGTTCAA    3180
                                ************************* ******** ** ************** ** ****
```

```
NC_002728_Nipah_Reference         GTGAAGTGATAGTCGGCATTTCTCCTGAAGATGAAGAGCCTTCAAGTGTTGGCGGAAAAC    3240
PP554504_Bat_2023_India           GTGAAGTGATAGTCGGCATTTCTCCTGAAGAGGAAGAGCCTTCAAGTGNNNNNNNNNNNN    3182
JN808863_Hu_2008_Bangladesh       GTGAAGTGATAGTCGGCATTTCTCCTGAAGAGGAAGAGCCTTCAAGTGCTGGCAGGAAAC    3240
                                  ******************************* ****************

NC_002728_Nipah_Reference         CCAATGAATCCATTGGACGTACAATCGAAGGCCAATCAATCCGAGACAACCTTCAAGCCA    3300
PP554504_Bat_2023_India           NNNNNNNNNNNNNNNNNNNNNNNNNNNNNNNNNNNNNNNNNCGAGACAGCCTCCAAATTA    3242
JN808863_Hu_2008_Bangladesh       CCATTGAATCTGCTGGACGTATAATTGAGGGCCAATCAACTCGAGACAGCCTCCAAATTA    3300
                                                                           ******* *** ***   *

NC_002728_Nipah_Reference         AGGACAACAAATCAACAGATGTACCAGGAGCAGGACCGAAAGATTCAGCAGTGAAGGAAG    3360
PP554504_Bat_2023_India           AGGGCAACAAGCCAGCAGATGCACCAGGAGCAGGACCGAAAGATTCAGCAGTGAAGGAAA    3302
JN808863_Hu_2008_Bangladesh       AGGGCAACAAGCCAGCAGATGCACCAGGAGCAGGACCGAAAGATTCAGCAGTGAAGGAAA    3360
                                  *** ******  ** ****** *************************************

NC_002728_Nipah_Reference         AACCACCCCAGAAGAGGCTACCTATGTTAGCTGAAGAATTTGAGTGCTCTGGATCGGAAG    3420
PP554504_Bat_2023_India           AATCACCCCAGAAGAGGCTGCCTATGTTAGCAGAAGAGTTTGAATGCTTTGGATCTGAAG    3362
JN808863_Hu_2008_Bangladesh       AATCACCCCAGAAGAGGCTGCCTATGTTAGCAGAAGAGTTTGAATGCTCTGGATCTGAAG    3420
                                  ** **************** *********** ***** ***** **** ****** ****

NC_002728_Nipah_Reference         ACCCAATCATTCGGGAGCTGCTGAAGGAGAACTCACTCATAAATTGTCAGCAAGGGAAAG    3480
PP554504_Bat_2023_India           ACCCAATTATCCGAGAGTTGCTGAAAGAGAATTCATTCATAAATAGTCAACAAGGGAAAG    3422
JN808863_Hu_2008_Bangladesh       ACCCAATTATCCAAGAGTTGCTGAAAGAGAATTCATTCATAAATAGTCAACAAGGGAAAG    3480
                                  ******* ** *  *** ******* ***** *** ******** **** **********

NC_002728_Nipah_Reference         ATGCTCAGCCTCCATATCATTGGAGCATCGAGAGGTCAATAAGCCCGGATAAAACTGAGA    3540
PP554504_Bat_2023_India           ATGCCCAGCCTCTATATTACAGGGGCATTGAGGGATCGAGAAGCCCAGACAAGACTGAGA    3482
JN808863_Hu_2008_Bangladesh       ATGCCCAGCCTCTATATTACAGGGGCATTGAGGGATCGAGAAGCCCAGACAAGGCTGAGA    3540
                                  **** ******* **** *  ** **** *** * ** * ****** ** **  ******

NC_002728_Nipah_Reference         TCGTCAACGGTGCTGTGCAAACTGCTGACAGGCAAAGACCAGGAACTCCGATGCCAAAGT    3600
PP554504_Bat_2023_India           TCACCAGCGATGTTGTACAAACTGCTAACAAGCAAAGACCAGGAACTCCGATGCCAAAAT    3542
JN808863_Hu_2008_Bangladesh       TCACCAGCGATGCTGTACAAACTGCTAACAAGCAAAGGCCAGGAACTCCGATGCCAAAAT    3600
                                  **  ** ** ** *** ********* *** ****** ******************** *

NC_002728_Nipah_Reference         CCCGAGGTATTCCCATTAAAAAGGGCACAGACGCGAAATATCCATCTGCTGGGACGGAAA    3660
PP554504_Bat_2023_India           CCCGAGGCATCCCTATTAAAAAGGGCACAGACGAGAAATATCCATCTGCTGGGACGGAAA    3602
JN808863_Hu_2008_Bangladesh       CCCGAGGCATCCCTATTAAAAAGGGCACAGACGAGAAATATCCATCTGCTGGGACGGAAA    3660
                                  ******* ** ** ******************* **************************

NC_002728_Nipah_Reference         ACGTGCCTGGGTCGAAGAGTGGTGCAACCCGGCATGTTCGAGGATCACCCCCCTACCAAG    3720
PP554504_Bat_2023_India           ACGTGCCTGGGTCGAAGAGTGGTGCAACCCGGCATGTTCGAGGATCACCCCCCTACCAAG    3662
JN808863_Hu_2008_Bangladesh       ACGTGCCTGGGTCGAAGAGTGGTGCAACCCGGCATGTTCGAGGATCACCCCCCTACCAAG    3720
                                  ************************************************************

NC_002728_Nipah_Reference         AAGGCAAGAGTGTCAATGCGGAGAATGTCCAACTGAATGCTTCCACTGCGGTTAAGGAAA    3780
PP554504_Bat_2023_India           AAGGCAAGAGTGTCAATGCGGAGAATGTCCAATTGAATGTTCCCACTGTTGTGAAGGAAA    3722
JN808863_Hu_2008_Bangladesh       AAGGCAAGAGTGTCAATGCGGAGAATGTCCAATTGAATGTTCCCACTGTTGTGAAGGAAA    3780
                                  ******************************** ****** * ******  ** *******

NC_002728_Nipah_Reference         CTGATAAGTCAGAAGTAAACCCCGTAGACGACAACGACTCACTTGATGATAAATACATCA    3840
PP554504_Bat_2023_India           CTGATAAATCAGAAGCAAACCCTGCAGATGACAACGACTCACTTGATGATAAATATATCA    3782
JN808863_Hu_2008_Bangladesh       CTGATAAATCAGAAGCAAACCCTGCAGATGACAACGACTCACTTGATGATAAATATATCA    3840
                                  ******* ******* ****** * *** ************************** ****

NC_002728_Nipah_Reference         TGCCTTCAGATGATTTCTCAAACACTTTCTTCCCGCACGACACTGATCGCTTGAATTATC    3900
PP554504_Bat_2023_India           TGCCTTCAGATGATTTCTCAAACACTTTCTTCCCGCATGACACTGATCGCTTAAATTATC    3842
JN808863_Hu_2008_Bangladesh       TGCCTTCAGATGATTTCTCAAACACTTTCTTCCCGCATGACACTGATCGCTTAAATTATC    3900
                                  ************************************* ************** *******

NC_002728_Nipah_Reference         ACGCAGATCATTTAGGTGATTATGACCTTGAAACCCTGTGTGAAGAGTCGGTTCTAATGG    3960
PP554504_Bat_2023_India           ACGCAGACCACTTAGGTGATTATGACCTCGAAACCCTGTGCGAAGAATCAGTCTTGATGG    3902
JN808863_Hu_2008_Bangladesh       ACGCAGACCACTTAGGTGATTATGACCTCGAAACCCTGTGCGAAGAATCAGTTTTGATGG    3960
                                  ******* ** ***************** *********** ***** ** **  * ****

NC_002728_Nipah_Reference         GAGTGATCAACTCTATAAAATTAATTAATCTGGATATGCGCTTAAATCACATTGAAGAAC    4020
PP554504_Bat_2023_India           GAGTAATCAACTCTATCAAATTAATTAATCTGGACATGCGCCTGAATCACATTGAAGAAC    3962
JN808863_Hu_2008_Bangladesh       GAGTAATCAACTCTATCAAATTAATTAATCTGGACATGCGCCTGAATCACATTGAAGAAC    4020
                                  **** *********** ***************** ****** * ****************

NC_002728_Nipah_Reference         AAGTTAAAGAGATCCCAAAGATCATCAATAAGCTTGAGTCCATTGACAGAGTTCTGGCCA    4080
PP554504_Bat_2023_India           AAGTTAAAGAGATCCCAAAGATCATCAATAAACTTGAGTCTATTGACAGGGTTCTGGCCA    4022
JN808863_Hu_2008_Bangladesh       AAGTTAAAGAGATCCCGAAGATCATCAATAAACTTGAGTCTATTGACAGGGTTCTGGCCA    4080
                                  **************** ************** ******** ******** **********

NC_002728_Nipah_Reference         AGACTAACACCGCACTCTCAACCATTGAAGGACACCTGGTTTCCATGATGATAATGATAC    4140
PP554504_Bat_2023_India           AAACCAATACTGCACTCTCAACTATTGAAGGACACTTGGTTTCCATGATGATAATGATAC    4082
JN808863_Hu_2008_Bangladesh       AAACCAATACTGCACTCTCAACTATTGAAGGACACTTGGTTTCCATGATGATAATGATAC    4140
                                  * ** ** ** *********** ************ ************************

NC_002728_Nipah_Reference         CAGGGAAAGGGAAAGGAGAAAGAAAGGGGAAAAATAATCCTGAGCTTAAACCAGTGATAG    4200
PP554504_Bat_2023_India           CGGGGAAGGGGAAAGGAGAAAGGAAAGGCAAAAGTAATCCTGAGCTTAAACCAGTGATAG    4142
JN808863_Hu_2008_Bangladesh       CGGGGAAGGGGAAAGGAGAAAGAAAAGGCAAAAGTAATCCTGAGCTTAAACCAGTGATAG    4200
                                  * ***** ************** ** ** **** **************************

NC_002728_Nipah_Reference         GAAGAGACATTCTAGAGCAGCAATCTCTTTTTCTTTTGACAATGTCAAGAATTTCAGAG    4260
PP554504_Bat_2023_India           GAAGAGATGTTCTGGAGCAGCAATCTCTTTTCTCATTTGACAATGTCAAGAATTTCAGAG    4202
```

```
JN808863_Hu_2008_Bangladesh      GAAGAGATGTTCTGGAGCAGCAATCTCTTTTCTCATTTGACAATGTCAAGAATTTCAGAG      4260
                                 *******  **** ***************** ** *************************

NC_002728_Nipah_Reference        ATGGATCGTTGACAAACGAACCGTATGGGGCAGCTGTACAGTTGAGAGAAGATCTTATTC      4320
PP554504_Bat_2023_India          ATGGGTCGTTGACAAATGAACCATATGGGGCAGCNNNNNNNNNNNNNNNNNATCTGATTC      4262
JN808863_Hu_2008_Bangladesh      ATGGGTCGTTGACAAATGAACCGTATGGGGCAGCTGTCCAATTGAGAGGAGATCTGATTC      4320
                                 **** *********** ***** ***********                 **** ****

NC_002728_Nipah_Reference        TTCCTGAACTTAATTTTGAGGAGACAAATGCATCTCAATTTGTTCCTATGGCAGATGATT      4380
PP554504_Bat_2023_India          TTCCTGAACTCAATTTTGAGGAAACGAATGCGTCTCAGTTTGTTCCCATGGCAGATGATT      4322
JN808863_Hu_2008_Bangladesh      TTCCTGAACTCAATTTTGAGGAAACGAATGCGTCTCAGTTTGTTCCCATGGCAGATGATT      4380
                                 ********** *********** ** ***** ***** ******** *************

NC_002728_Nipah_Reference        CATCCAGAGATGTTATCAAGACATTGATAAGGACTCACATTAAAGATAGAGAGTTGAGAT      4440
PP554504_Bat_2023_India          CGTCCAGAGACGTTGTCAAGACATTGATAAGGACTCATATTAAGGATAGAGAATTGAGAT      4382
JN808863_Hu_2008_Bangladesh      CGTCCAGAGACGTTGTCAAGACATTGATAAGGACTCATATTAAGGATAGAGAATTGAGAT      4440
                                 * ******** *** ********************** ***** ******** *******

NC_002728_Nipah_Reference        CAGAACTGATTGGTTACCTGAATAAAGCGGAAAATGATGAGGAAATTCAGGAGATAGCGA      4500
PP554504_Bat_2023_India          CAGAACTGATTGGTTATCTGAATAGAGCAGAAAATGATGAGGAAATTCAGGAGATAGCAA      4442
JN808863_Hu_2008_Bangladesh      CAGAACTGATTGGTTATCTGAATAGAGCAGAAAATGATGAGGAAATTCAGGAGATAGCAA      4500
                                 **************** ******** *** **************************** *

NC_002728_Nipah_Reference        ACACTGTCAATGACATCATTGACGGTAATATTTGATCACTGAATTGTCAGCAGAAATACA      4560
PP554504_Bat_2023_India          ATACTGTCAATGACATTATTGACGGTAACATTTGATCACTGAATTGTCAGCAGAAATGAG      4502
JN808863_Hu_2008_Bangladesh      ATACTGTCAATGACATTATTGACGGTAACATTTGATCACTGAATTGTCAGCAGAAATGAG      4560
                                 * ************** *********** ****************************

NC_002728_Nipah_Reference        ATGATCTAACAACAATCTCCCACAAGTAGACAATGGTTTCAGGTCAATAATAACAACCTC      4620
PP554504_Bat_2023_India          ATGATCCAACAACAATCCTCTACATGTAGACAGTGGTTTCAGGTCAGTGACAACAACCTC      4562
JN808863_Hu_2008_Bangladesh      ATGATCCAACAACAATCCTCTACATGTAGACAGTGGTTTCAGGTCAGTGACAACAACCTC      4620
                                 ****** **********  * *** ******* ************* * * *********

NC_002728_Nipah_Reference        AATACTAATCTTTCACATAAGCATTACTCATTCCAGCCCTCAGACGATAACACAATACTT      4680
PP554504_Bat_2023_India          ACTACTAATCTTTCACATAAGCATTACCCATTCTAGCTCTCGGGCGATAACGCAGTACTT      4622
JN808863_Hu_2008_Bangladesh      ACTACTAATCTTTCACATAAGCATTACCCATTCTAGCTCTCGGGCGATAACACAGTACTT      4680
                                 * ************************* ***** *** *** * ******* ** *****

NC_002728_Nipah_Reference        GATACATGTTTATTGAAGTGTATGTAGCATGATTGAACTATTCAATAACTGTATTTCTCA      4740
PP554504_Bat_2023_India          GATACATGTTTATTGAAGTGTATGTAGCATGATCAAATCACTTAATAACTGTACCTCTTA      4682
JN808863_Hu_2008_Bangladesh      GATACATGTTTATTGAAGTGTATGTAGCATGATCAAATCACTTAATAACTGTACCTCTTA      4740
                                 *********************************  **  * * **********  *** *

NC_002728_Nipah_Reference        CTCTTGCTCTTAGTTAGTCATTGTGTCTAATAATTATTATTACAGTACAAGGTATTATGA      4800
PP554504_Bat_2023_India          ATCTTGCTCTTAGTTAGTCACTGCATCTAATAATTATTATTACAGTACAAGGTATCATGA      4742
JN808863_Hu_2008_Bangladesh      ATCTTGCTCTTAGTTAGTCACTGCATCTAATAATTATTATTACAGTACAAGGTATTATGA      4800
                                  ******************* **  ****************************** ****

NC_002728_Nipah_Reference        ATTCAAAGATACGCAATAAATCTGATATCAGCATAGAGTAGAAAATTGTTGTTTTTGTCA      4860
PP554504_Bat_2023_India          ACACAAAGATACGCAATAAATCTGATATCAGCATGGAGCAGAGAATTATTGTTTTTATCA      4802
JN808863_Hu_2008_Bangladesh      ACACAAAGATACGCAATAAATCTGATATCAGCATGGAGCAGAGAATTATTGTTTTTATCA      4860
                                 *  ******************************* *** *** **** ******** ***

NC_002728_Nipah_Reference        TGATCATTCGAAGATTTAACAATGATGTCAACTTTCATACCTAAACATAATAACATAAAA      4920
PP554504_Bat_2023_India          TGACAATTTGAAAGTCCAACAGTGATGTCAACTTTCATACATAAGTATAATACTATAAAA      4862
JN808863_Hu_2008_Bangladesh      TGATAATTTGAAAGTCCAACAGTGATGTCAACTTTCATACATAAGTATAATACTATAAAA      4920
                                 ***  *** ***  *  **** ****************** ***  ******  ******

NC_002728_Nipah_Reference        TGGTCGATTTGTATTGTAGATCTCTCACGCATTTTAGTGTCATGAATTAGTGTTTCAAAT      4980
PP554504_Bat_2023_India          TGGTCGATTCGTATTGTAGTTCATTCACGCATTTTAGTGTTATAAATTAGTGTTTCAAAT      4922
JN808863_Hu_2008_Bangladesh      TGGTCGATTCGTATTGTAGTTTATTCACGCATTTTAGTGTTATAAATTAGTGTTTCAAAT      4980
                                 ********* ********* *   ***************** ** ***************

NC_002728_Nipah_Reference        CAGTTGCATATCAATTAAGAAAAACTTAGGAGACAGGTATAGAACCTCTCTTT-CAGATA      5039
PP554504_Bat_2023_India          CAGTTGCATATCAATTAAGAAAAACTTAGGAGACAGGTATAGGACTTCTCTCCACAAATA      4982
JN808863_Hu_2008_Bangladesh      CAGTTGCATATCAATTAAGAAAAACTTAGGAGACAGGTATAGGACTTCTCTCT-CCAATA      5039
                                 ****************************************** ** *****   *  ***

NC_002728_Nipah_Reference        ACTGGTCAATTAAGGACAGAAATTCTGTTTCTCAAATCCGCTAGCCTTTGTCAAAGAGGA      5099
PP554504_Bat_2023_India          ACTGGTCAATTGAGGACAGAAATCCTGTTTCTCAAATTCACCAGCCCTTGCCAAGAAGGA      5042
JN808863_Hu_2008_Bangladesh      ACTGGTCAATTGAGGACAGAAATCCTGTTTCTCAAATTCACCAGCCCTTGCCAAGAAGGA      5099
                                 *********** *********** ************* * * **** *** ***  ****

NC_002728_Nipah_Reference        CACAAGCAATGGAGCCGGACATCAAGAGTATTTCAAGTGAGTCAATGGAAGGAGTATCTG      5159
PP554504_Bat_2023_India          AGCAAGCAATGGAGCCAGACATCAAGAGCGTTTCAAGTGAGTCTATGGAAGGAGTATCTG      5102
JN808863_Hu_2008_Bangladesh      CGCAAGCAATGGAGCCAGACATCAAGAGCATTTCAAGTGAGTCTATGGAAGGAGTATCTG      5159
                                   ************** ***********  ************* ****************

NC_002728_Nipah_Reference        ATTTCAGCCCTAGTTCTTGGGAGCATGGTGGGTATCTTGATAAGGTTGAACCAGAAATTG      5219
PP554504_Bat_2023_India          ATTTTAGCCCTAGTTCTTGGGAGAATGGTGGGTATCTTGATAAGGTTGAGCCAGAAATCG      5162
JN808863_Hu_2008_Bangladesh      ATTTTAGCCCTAGTTCTTGGGAGAATGGTGGGTATCTTGATAAGGTTGAGCCAGAAATCG      5219
                                 **** ****************** ************************** ******* *

NC_002728_Nipah_Reference        ATGAAAATGGCAGTATGATTCCAAAATACAAGATCTATACCCCAGGAGCTAACGAGAGGA      5279
PP554504_Bat_2023_India          ATGAAAATGGCAGTATGATTCCCAAATACAAGATCTACACCCCAGGAGCTAACGAGAGGA      5222
JN808863_Hu_2008_Bangladesh      ATGAAAATGGCAGTATGATTCCCAAATACAAGATCTACACCCCAGGAGCTAACGAGAGGA      5279
                                 ********************** ************** **********************
```

```
NC_002728_Nipah_Reference       AATACAACAACTACATGTACCTTATATGTTACGGCTTTGTTGAAGATGTTGAGAGAACCC     5339
PP554504_Bat_2023_India         AATACAATAACTACATGTACCTTATATGTTATGGCTTTGTTGAAGATGTTGAGCGAACCC     5282
JN808863_Hu_2008_Bangladesh     AATACAATAACTACATGTACCTTATATGTTATGGCTTTGTTGAAGATGTTGAGCGAACCC     5339
                                ******* ********************** ********************** ******

NC_002728_Nipah_Reference       CAGAGACAGGGAAACGCAAGAAGATCAGGACAATTGCTGCCTACCCTCTGGGTGTTGGTA     5399
PP554504_Bat_2023_India         CAGAGACAGGGAAGCGTAAGAAGATCAGGACAATTGCTGCCTACCCTTTGGGTGTTGGCA     5342
JN808863_Hu_2008_Bangladesh     CAGAGACAGGGAAGCGTAAGAAGATCAGGACAATTGCTGCCTACCCTTTGGGTGTTGGCA     5399
                                ************* ** ****************************** ********** *

NC_002728_Nipah_Reference       AGAGTGCCTCTCATCCCCAAGATCTTCTGGAGGAACTCTGTTCCCTCAAAGTTACTGTGA     5459
PP554504_Bat_2023_India         AGAGTGCCTCTCATCCTCAAGATCTTCTGGAGGAACTCTGTTCCCTCAAAGTCACTGTGA     5402
JN808863_Hu_2008_Bangladesh     AGAGTGCCTCTCATCCTCAAGATCTTCTGGAGGAACTCTGTTCCCTCAAAGTCACTGTGA     5459
                                **************** *********************************** *******

NC_002728_Nipah_Reference       GAAGAACAGCTGGATCAACTGAGAAAATTGTGTTTGGATCATCTGGCCCTCTAAATCACC     5519
PP554504_Bat_2023_India         GGAGAACAGCTGGATCAACTGAGAAAGTTGTATTCGGATCATCTGGACCTCTAAATCACC     5462
JN808863_Hu_2008_Bangladesh     GGAGAACAGCTGGATCAACTGAGAAAGTTGTATTCGGATCATCTGGACCTCTAAATCACC     5519
                                * ************************ **** ** *********** *************

NC_002728_Nipah_Reference       TCGTTCCGTGGAAGAAAGTACTGACTAGTGGTTCAATTTTTAATGCAGTCAAGGTTTGTC     5579
PP554504_Bat_2023_India         TTGTCCCGTGGAAGAAAGTATTGACCGGTGGTTCAATTTTCAATGCAGTTAAGGTCTGCC     5522
JN808863_Hu_2008_Bangladesh     TTGTCCCGTGGAAGAAAGTATTGACCGGTGGTTCAATTTTCAATGCAGTTAAGGTCTGCC     5579
                                * ** *************** ****  ************* ******** ***** ** *

NC_002728_Nipah_Reference       GGAACGTTGATCAGATACAGCTTGACAAGCATCAAGCTCTGAGAATATTTTTTCTCAGTA     5639
PP554504_Bat_2023_India         GAAACGTTGATCAGATACAGCTCGACAAGCATCAAGCCCTGAGAATATTTTTTCTCAGTA     5582
JN808863_Hu_2008_Bangladesh     GAAACGTTGATCAGATACAGCTCGACAAGCATCAAGCCCTGAGAATATTTTTTCTCAGTA     5639
                                * ******************** ************** **********************

NC_002728_Nipah_Reference       TCACAAAGCTCAATGATTCTGGAATCTACATGATTCCACGAACCATGCTTGAGTTCAGGA     5699
PP554504_Bat_2023_India         TTACAAAACTCAACGATTCCGGAATCTACATGATTCCACGAACTATGCTTGAGTTCAGGA     5642
JN808863_Hu_2008_Bangladesh     TTACAAAACTCAACGATTCTGGAATCTACATGATTCCACGAACTATGCTTGAGTTCAGGA     5699
                                * ***** ***** ***** *********************** ****************

NC_002728_Nipah_Reference       GAAACAATGCCATTGCCTTCAATCTTCTAGTGTACTTGAAGATTGATGCTGATTTATCCA     5759
PP554504_Bat_2023_India         GAAACAATGCCATTGCGTTCAATCTTCTGGTGTACCTGAAGATTGATGCTGATCTATCCA     5702
JN808863_Hu_2008_Bangladesh     GAAACAATGCCATTGCGTTCAATCTTCTGGTGTACCTGAAGATTGATGCTGATCTATCCA     5759
                                **************** *********** ****** ***************** ******

NC_002728_Nipah_Reference       AAATGGGGATCCAGGGAAGCCTCGATAAAGATGGCTTCAAGGTTGCCTCCTTCATGCTAC     5819
PP554504_Bat_2023_India         AAATGGGAATCCAGGGAAGCCTCGACAAAGATGGCTTCAAAGTTGCTTCCTTCATGCTGC     5762
JN808863_Hu_2008_Bangladesh     AAATGGGAATCCAGGGAAGCCTCGACAAAGATGGCTTCAAAGTTGCTTCCTTCATGCTGC     5819
                                ******* ***************** ************** ***** *********** *

NC_002728_Nipah_Reference       ACTTGGGGAACTTTGTCCGTCGTGCAGGGAAGTATTACTCTGTTGATTATTGTAGGAGGA     5879
PP554504_Bat_2023_India         ACTTGGGAAACTTTGTCCGTCGCGCAGGAAAGTACTATTCTGTTGATTATTGTAGGAGAA     5822
JN808863_Hu_2008_Bangladesh     ACTTGGGAAACTTTGTCCGTCGCGCAGGAAAGTACTATTCTGTTGATTATTGTAGGAGAA     5879
                                ******* ************** ***** ***** ** ******************** *

NC_002728_Nipah_Reference       AGATTGATAGGATGAAATTGCAGTTTTCACTGGGTTCCATAGGCGGACTAAGTCTCCACA     5939
PP554504_Bat_2023_India         AGATTGATAGGATGAAATTGCAGTTTTCATTGGGTTCCATAGGTGGGTTAAGTCTCCACA     5882
JN808863_Hu_2008_Bangladesh     AGATTGATAGGATGAAATTGCAGTTTTCATTGGGTTCCATAGGTGGGTTAAGTCTCCACA     5939
                                ***************************** ************* **  ************

NC_002728_Nipah_Reference       TTAAGATCAATGGTGTAATCAGCAAACGGCTGTTTGCTCAAATGGGATTCCAAAAAAACC     5999
PP554504_Bat_2023_India         TTAAGATCAATGGTGTAATCAGCAAACGTCTGTTTGCTCAAATGGGTTTCCAAAAAAACC     5942
JN808863_Hu_2008_Bangladesh     TTAAGATCAATGGTGTAATCAGCAAACGTCTGTTTGCTCAAATGGGTTTCCAAAAAAACC     5999
                                **************************** ***************** *************

NC_002728_Nipah_Reference       TTTGTTTCTCTTTGATGGACATCAATCCTTGGCTCAACAGATTGACCTGGAACAACAGTT     6059
PP554504_Bat_2023_India         TTTGTTTTTCCTTGATGGATATTAATCCTTGGCTCAACAGACTAACCTGGAACAACAGCT     6002
JN808863_Hu_2008_Bangladesh     TTTGTTTTTCCTTGATGGATATTAATCCTTGGCTCAACAGACTAACCTGGAACAACAGCT     6059
                                ******* ** ******** ** ****************** * ************** *

NC_002728_Nipah_Reference       GTGAGATCAGCCGAGTAGCAGCTGTGTTGCAGCCTTCTATTCCAAGAGAGTTCATGATCT     6119
PP554504_Bat_2023_India         GTGAGATCAGCCGAGTGGCAGCTGTGTTGCAGCCTTCTGTTCCAAGAGAGTTCATGATCT     6062
JN808863_Hu_2008_Bangladesh     GTGAGATCAGCCGAGTGGCAGCTGTGTTGCAGCCTTCTGTTCCGAGAGAGTTCATGATCT     6119
                                **************** ********************* **** ****************

NC_002728_Nipah_Reference       ATGATGATGTCTTCATTGACAATACAGGGAGAATTCTAAAGGGCTAAACAGAATTCTTCT     6179
PP554504_Bat_2023_India         ATGATGATGTCTTCATTGACAATACNNNNNNNNNNNNNNNNNNNNNNNNNNNNNNNNNNN     6122
JN808863_Hu_2008_Bangladesh     ATGATGATGTCTTCATTGACAATACAGGGAGAATTCTAAAGGGCTAAGTAGAATCCTTTT     6179
                                *************************

NC_002728_Nipah_Reference       AAAATTTAATCAGTCATGAGTTTAGTAATCATACCTAGTCATAATACATCACACAGGACT     6239
PP554504_Bat_2023_India         NNNNNNNNNNNNNNNNNNNNNNNNNNNNNNNNNNNNNNNNNTAATACATCACGCAGGACT     6182
JN808863_Hu_2008_Bangladesh     CAAATTTAATCAATTATGGATTTAGTAATCTTATCTGGTCTTAATACATCACGCAGGACT     6239
                                                                         *********** *******

NC_002728_Nipah_Reference       ATTTACAAAAGACAGTTAAAAAATGGAATAATCATGTAGTAGTAATTGAGAACATTATTA     6299
PP554504_Bat_2023_India         ATTTACAAAAACCAGCAAAAAAATAGAATAATCATGTAGTAGTAATTGAAGACATCATTA     6242
JN808863_Hu_2008_Bangladesh     ATTTACAAAAACCAGCAAAAAAATAGAATAATCATGTAGTAGTAATTGAAGACATCATTA     6299
                                **********  ***  ******* ************************  **** ****

NC_002728_Nipah_Reference       GAATAGTATAACTAAAATGTAGTTTTTTTGAGTATTTGATTTAAAATTAGATAACTATTA     6359
PP554504_Bat_2023_India         AAATAGTGTAGTTAAAATATAGTTTTTGTGAATATTCGGTTAATAATTAGACAACTATTA     6302
JN808863_Hu_2008_Bangladesh     AAATAGTGTAGTTAAAATGTAGTTTTTGTGAATATTCGGTTAATAATTAGACAACTATTA     6359
```

```
                                 ****** **  ****** ******** *** **** * ** * ******* ********

NC_002728_Nipah_Reference       CAAAAAACTTAGGAGCCAAGCTCTTGCCTCGTTCAGAAGGTTAAACAAGCATTCTTACCA     6419
PP554504_Bat_2023_India         TGAAAAACTTAGGAGCCAAGCTCTCGCCCTTTTCAAAAGATTAAACAAGCATTATTACTA     6362
JN808863_Hu_2008_Bangladesh     TGAAAAACTTAGGAGCCAAGCTCTCGCCTTTTTCAAAAGATTAAACAAGCATTATTACTA     6419
                                  ********************** ***   **** *** ************* **** *

NC_002728_Nipah_Reference       TTGGATCAACAAAAGGATTGGTTTTATCGTCTAAGAAATTTATTGAAAGGCAAAGAAATT     6479
PP554504_Bat_2023_India         TCGGATCAACAAAAGGATTGGTTTAATCNNNNNNNNNNNNNNNNNNNNNNNNNNNNNNNN     6422
JN808863_Hu_2008_Bangladesh     TCGGATCAACAAAAGGATTGGTTTAATCATCTAAGAAATCTCTCGAAAGACAAAGAGATT     6479
                                * ********************** ***

NC_002728_Nipah_Reference       CCTGGTTTTATGTTGAATGAGGTGTATCAAACTAAGGAGACCTTCTAACAGCCAGGTCAT     6539
PP554504_Bat_2023_India         NNNNNNNNNNNNNNNNNNNNNNNNNNNNNNNNNNNNNNNNNNNNNNNNNNNNNNNNNNNN     6482
JN808863_Hu_2008_Bangladesh     CTAGGTTTTATGTTGAAGGAAGTGTGTTAAACCTAGGAGACCCTTCAACAGTCAGGTCGC     6539

NC_002728_Nipah_Reference       AGGAATATAA------ATAAAAATAAGAATAAAATTGATTCCATCGGAAGATTCATTTCA     6593
PP554504_Bat_2023_India         NNNNNNNNAA------ACAAAAATAAAAATAAAATTGATTCTGTCAGAAGATTCATTCCA     6536
JN808863_Hu_2008_Bangladesh     GGGAATACAAACAAAGACAAAAACAAAAATAAAATTGATTCTGTCAGAAGATTCATTCCG     6599
                                        **       * ***** ** *************  ** *********** *

NC_002728_Nipah_Reference       AGAAGTGATCAAATCAAAGCGGTTGGCAGACCTACCAATCATATACCACAAGACTCGACA     6653
PP554504_Bat_2023_India         AAAAGTGATCAAATCAAAGCAGTTGGTAGACCTATCAATCATATCCCGTAAGACTCGACA     6596
JN808863_Hu_2008_Bangladesh     AAAAGTGATCAAATCAAAGCAGTTGGTAGACCTATCAATCATATCCCGTAAGACTCGACA     6659
                                * ****************** ***** ******* ********* **  **********

NC_002728_Nipah_Reference       ATGGTAGTTATACTTGACAAGAGATGTTATTGTAATCTTTTAATATTGATTTTGATGATC     6713
PP554504_Bat_2023_India         ATGGCAGTCATACTTAATAAGAGATATTATTCTAATCTCTTAATACTGATTTTGATTATC     6656
JN808863_Hu_2008_Bangladesh     ATGGCAGTCATACTTAACAAGAGATATTATTCTAATCTCTTAATACTGATTTTGATAATC     6719
                                **** *** ****** * ******* ***** ****** ****** ********** ***

NC_002728_Nipah_Reference       TCGGAGTGTAGTGTTGGGATTCTACATTATGAGAAATTGAGTAAAATTGGACTTGTCAAA     6773
PP554504_Bat_2023_India         TCGGAGTGCAGTGTCGGGATTTTGCATTATGAGAAATTGAGTAAGATTGGGCTTGTCAAA     6716
JN808863_Hu_2008_Bangladesh     TCGGAGTGCAGTGTCGGGATTTTGCATTATGAGAAATTGAGTAAGATTGGGCTTGTCAAA     6779
                                ******** ***** ****** * ******************** ***** *********

NC_002728_Nipah_Reference       GGAGTAACAAGAAAATACAAGATTAAAAGCAATCCTCTCACAAAAGACATTGTTATAAAA     6833
PP554504_Bat_2023_India         GGAATAACAAGAAAATACAAGATCAAAAGCAATCCTCTCACAAAAGACATTGTTATTAAA     6776
JN808863_Hu_2008_Bangladesh     GGAATAACAAGAAAATACAAGATCAAAAGCAATCCTCTCACAAAAGACATTGTTATTAAA     6839
                                *** ******************* ******************************** ***

NC_002728_Nipah_Reference       ATGATTCCGAATGTGTCGAACATGTCTCAGTGCACAGGGAGTGTCATGGAAAATTATAAA     6893
PP554504_Bat_2023_India         ATGATTCCGAATGTGTCAAACATGTCTCAATGCACGGGGAGTGTCATGGAAAATTATAAA     6836
JN808863_Hu_2008_Bangladesh     ATGATTCCGAATGTGTCAAACATGTCTCAATGCACGGGGAGTGTCATGGAAAATTATAAA     6899
                                ***************** *********** ***** ************************

NC_002728_Nipah_Reference       ACACGATTAAACGGTATCTTAACACCTATAAAGGGAGCGTTAGAGATCTACAAAAACAAC     6953
PP554504_Bat_2023_India         ACACGATTAAACGGTATCCTAACGCCTATAAAGGGGGCATTAGAGATTTACAAGAACAAC     6896
JN808863_Hu_2008_Bangladesh     ACACGATTAAACGGTATCCTAACGCCTATAAAGGGGGCATTAGAGATTTACAAGAACAAC     6959
                                ****************** **** *********** ** ******** ***** ******

NC_002728_Nipah_Reference       ACTCATGACCTTGTCGGTGATGTGAGATTAGCCGGAGTTATAATGGCAGGAGTTGCTATT     7013
PP554504_Bat_2023_India         ACTCATGACCTTGTCGGTGATGTAAGACTGGCCGGAGTTATAATGGCAGGAGTTGCTATT     6956
JN808863_Hu_2008_Bangladesh     ACTCATGACCTTGTCGGTGATGTAAGACTGGCCGGAGTTATAATGGCAGGAGTTGCTATT     7019
                                *********************** *** * ******************************

NC_002728_Nipah_Reference       GGGATTGCAACCGCAGCTCAAATCACTGCAGGTGTAGCACTATATGAGGCAATGAAGAAT     7073
PP554504_Bat_2023_India         GGAATTGCAACCGCAGCTCAAATTACTGCAGGTGTAGCATTATATGAGGCAATGAAAAAT     7016
JN808863_Hu_2008_Bangladesh     GGAATTGCAACCGCAGCTCAAATTACTGCAGGTGTAGCATTATATGAGGCAATGAAAAAT     7079
                                ** ******************** *************** **************** ***

NC_002728_Nipah_Reference       GCTGACAACATCAACAAACTCAAAAGCAGCATTGAATCAACTAATGAAGCTGTCGTTAAA     7133
PP554504_Bat_2023_India         GCTGACAACATCAACAAACTCAAAAGCAGCATAGAATCAACTAATGAAGCTGTTGTTAAG     7076
JN808863_Hu_2008_Bangladesh     GCTGACAACATCAACAAACTCAAAAGCAGCATAGAATCAACTAATGAAGCTGTTGTTAAG     7139
                                ******************************** ******************** *****

NC_002728_Nipah_Reference       CTTCAAGAGACTGCAGAAAAGACAGTCTATGTGCTGACTGCTCTACAGGATTACATTAAT     7193
PP554504_Bat_2023_India         CTTCAAGAGACTGCAGAAAAGACAGTCTATGTACTGACTGCTTTGCAGGATTACATTAAT     7136
JN808863_Hu_2008_Bangladesh     CTTCAAGAGACTGCAGAAAAGACAGTCTATGTACTAACTGCTTTGCAGGATTACATTAAT     7199
                                ******************************** ** ****** * ***************

NC_002728_Nipah_Reference       ACTAATTTAGTACCGACAATTGACAAGATAAGCTGCAAACAGACAGAACTCTCACTAGAT     7253
PP554504_Bat_2023_India         ACTAACTTGGTACCGACAATTGACAAGATAAGCTGCAAACAGACGGAACTCTCATTAGAT     7196
JN808863_Hu_2008_Bangladesh     ACTAACTTGGTACCGACAATTGACAAGATAAGCTGCAAACAGACGGAACTCTCATTAGAT     7259
                                ***** ** *********************************** ********* *****

NC_002728_Nipah_Reference       CTGGCATTATCAAAGTACCTCTCTGATTTGCTTTTTGTATTTGGCCCCAACCTTCAAGAC     7313
PP554504_Bat_2023_India         CTAGCACTATCAAAGTACCTCTCTGATTTGCTTTTTGTATTTGGTCCCAACCTTCAAGAC     7256
JN808863_Hu_2008_Bangladesh     CTAGCACTATCAAAGTACCTCTCTGATTTGCTTTTTGTATTTGGTCCCAACCTTCAAGAC     7319
                                ** *** ************************************* ***************

NC_002728_Nipah_Reference       CCAGTTTCTAATTCAATGACTATACAGGCTATATCTCAGGCATTCGGTGGAAATTATGAA     7373
PP554504_Bat_2023_India         CCAGTTTCTAATTCAATGACTATACAGGCTATATCTCAGGCATTCGGTGGAAATTATGAA     7316
JN808863_Hu_2008_Bangladesh     CCAGTTTCTAATTCAATGACTATACAGGCTATATCTCAGGCATTCGGTGGAAATTATGAA     7379
                                ************************************************************

NC_002728_Nipah_Reference       ACACTGCTAAGAACATTGGGTTACGCTACAGAAGACTTTGATGATCTTCTAGAAAGTGAC     7433
```

```
PP554504_Bat_2023_India         ACACTGCTAAGAACGTTGGGTTATGCTACAGAAGACTTTGATGATCTTCTAGAAAGTGAC    7376
JN808863_Hu_2008_Bangladesh     ACACTGCTAAGAACGTTGGGTTACGCTACAGAAGACTTTGATGATCTTCTAGAAAGTGAC    7439
                                ************** ******** ************************************

NC_002728_Nipah_Reference       AGCATAACAGGTCAAATCATCTATGTTGATCTAAGTAGCTACTATATAATTGTCAGGGTT    7493
PP554504_Bat_2023_India         AGCATAACGGGTCAAATTATCTACGTTGATTTAAGTGGCTACTACATAATTGTCAGGGTT    7436
JN808863_Hu_2008_Bangladesh     AGCATAACGGGTCAAATTATCTACGTTGATTTAAGTGGCTACTACATAATTGTCAGGGTT    7499
                                ******** ******** ***** ****** ***** ******* ***************

NC_002728_Nipah_Reference       TATTTTCCTATTCTGACTGAAATTCAACAGGCCTATATCCAAGAGTTGTTACCAGTGAGC    7553
PP554504_Bat_2023_India         TATTTTCCTATNNNNNNNNNNNNNNNNNNNNNNNNNATCCAAGAATTGTTGCACGTGCGC    7496
JN808863_Hu_2008_Bangladesh     TATTTTCCTATCCTGACTGAAATCCAACAGGCCTATATCCAAGAATTGTTGCCCGTGAGC    7559
                                ***********                         ******** ***** *  *** **

NC_002728_Nipah_Reference       TTCAACAATGATAATTCAGAATGGATCAGTATTGTCCCAAATTTCATATTGGTAAGGAAT    7613
PP554504_Bat_2023_India         TATAAAAATTACAATTCAGAATGGATCAGCATTGTCCCAAATTTCATATTGGTAAGGAAC    7556
JN808863_Hu_2008_Bangladesh     TTTAACAATGACAATTCAGAATGGATCAGCATTGTCCCAAATTTCATATTGGTAAGGAAC    7619
                                *  ** *** * ***************** *****************************

NC_002728_Nipah_Reference       ACATTAATATCAAATATAGAGATTGGATTTTGCCTAATTACAAAGAGGAGCGTGATCTGC    7673
PP554504_Bat_2023_India         ACATTAATATCAAATATAGAGATTGGATTTTGCCTAATTACAAAGAGGAGTGTGATCTGC    7616
JN808863_Hu_2008_Bangladesh     ACATTAATATCAAATATAGAGATTGGATTTTGCCTAATTACAAAGAGGAGTGTGATCTGC    7679
                                ************************************************** *********

NC_002728_Nipah_Reference       AACCAAGATTATGCCACACCTATGACCAACAACATGAGAGAATGTTTAACGGGATCGACT    7733
PP554504_Bat_2023_India         AACCAAGATTATGCAACACCCATGACAAACAATATGAGGGAATGTTTGACGGGGTCGACT    7676
JN808863_Hu_2008_Bangladesh     AACCAAGATTATGCAACACCCATGACAAACAATATGAGGGAATGTTTGACGGGGTCGACT    7739
                                ************** ***** ***** ***** ***** ******** ***** ******

NC_002728_Nipah_Reference       GAGAAGTGTCCTCGAGAGCTGGTTGTTTCATCACATGTTCCCAGATTTGCACTATCTAAC    7793
PP554504_Bat_2023_India         GAGAAGTGTCCTCGAGAGCTGGTTGTTTCATCACACGTTCCCAGATTTGCACTATCTAAC    7736
JN808863_Hu_2008_Bangladesh     GAGAAGTGTCCTCGAGAGCTGGTTGTTTCATCACACGTTCCCAGATTTGCACTATCTAAC    7799
                                *********************************** ************************

NC_002728_Nipah_Reference       GGGGTTCTGTTTGCCAATTGCATAAGTGTTACATGTCAGTGTCAAACAACAGGCAGGGCA    7853
PP554504_Bat_2023_India         GGGGTTTTGTTTGCTAATTGCATAAGCGTCACATGCCAGTGTCAAACAACAGGTAGGGCA    7796
JN808863_Hu_2008_Bangladesh     GGGGTTTTGTTTGCTAATTGCATAAGCGTCACATGCCAGTGTCAAACAACAGGTAGGGCA    7859
                                ****** ******* *********** ** ***** ***************** ******

NC_002728_Nipah_Reference       ATCTCACAATCAGGAGAACAAACTCTGCTGATGATTGACAACACCACCTGTCCTACAGCC    7913
PP554504_Bat_2023_India         ATCTCACAGTCAGGAGAACAAACTCTGCTGATGATTGATAACACCACCTGTCCTACAGCT    7856
JN808863_Hu_2008_Bangladesh     ATCTCACAGTCAGGAGAACAAACTCTGCTGATGATTGATAACACCACCTGTCCTACAGCT    7919
                                ******** ***************************** ********************

NC_002728_Nipah_Reference       GTACTCGGTAATGTGATTATCAGCTTAGGGAAATATCTGGGGTCAGTAAATTATAATTCT    7973
PP554504_Bat_2023_India         GTACTCGGTAATGTGATCATCAGCTTGGGAAAATATCTTGGGTCAGTAAATTATAACTCT    7916
JN808863_Hu_2008_Bangladesh     GTACTCGGTAATGTGATCATCAGCTTGGGAAAATATCTTGGGTCAGTAAATTATAACTCT    7979
                                ***************** ******** ** ******** ***************** ***

NC_002728_Nipah_Reference       GAAGGCATTGCTATCGGTCCTCCAGTCTTTACAGATAAAGTTGATATATCAAGTCAGATA    8033
PP554504_Bat_2023_India         GAAGGCATTGCTATTGGTCCTCCTGTCTTTACTGATAAAGTTGACATATCAAGTCAAATA    7976
JN808863_Hu_2008_Bangladesh     GAAGGCATTGCTATTGGTCCTCCTGTCTTTACTGATAAAGTTGACATATCAAGTCAAATA    8039
                                ************** ******** ******** *********** *********** ***

NC_002728_Nipah_Reference       TCCAGCATGAATCAGTCCTTACAACAGTCTAAGGACTATATCAAAGAGGCTCAACGACTC    8093
PP554504_Bat_2023_India         TCTAGCATGAATCAGTCCTTACAGCAATCTAAGGACTATATCAAAGAAGCTCAACGACTC    8036
JN808863_Hu_2008_Bangladesh     TCTAGCATGAATCAGTCCTTACAGCAATCTAAGGACTATATCAAAGAAGCTCAACGACTC    8099
                                ** ******************** ** ******************** ************

NC_002728_Nipah_Reference       CTTGATACTGTTAATCCATCATTAATAAGCATGTTGTCTATGATCATACTGTATGTATTA    8153
PP554504_Bat_2023_India         CTTGATACTGTTAACCCGTCATTAATAAGCATGTTATCTATGATCATACTGTATGTACTA    8096
JN808863_Hu_2008_Bangladesh     CTTGATACTGTTAACCCGTCATTAATAAGCATGTTATCTATGATCATACTGTATGTACTA    8159
                                ************** ** ***************** ********************* **

NC_002728_Nipah_Reference       TCGATCGCATCGTTGTGTATAGGGTTGATTACATTTATCAGTTTTATCATTGTTGAGAAA    8213
PP554504_Bat_2023_India         TCAATTGCATCATTGTGTATAGGGTTGATTACATTTATCAGTTTTATCATTGTTGAGAAA    8156
JN808863_Hu_2008_Bangladesh     TCAATTGCATCATTGTGTATAGGGTTGATTACATTTATCAGTTTTATCATTGTTGAGAAA    8219
                                ** ** ***** ************************************************

NC_002728_Nipah_Reference       AAGAGAAACACCTACAGCAGATTAGAGGATAGGAGAGTCAGACCTACAAGCAGTGGGGAT    8273
PP554504_Bat_2023_India         AAAAGAAATACCTATAGCAGATTAGAGGACAGGAGGGTCAGACCTACAAGCAGTGGGGAT    8216
JN808863_Hu_2008_Bangladesh     AAAAGAAATACCTATAGCAGATTAGAGGACAGGAGGGTCAGACCTACAAGCAGTGGGGAT    8279
                                ** ***** ***** ************** ***** ************************

NC_002728_Nipah_Reference       CTCTACTACATTGGGACATAGTGTATTCAGATTGATGAAATTATGTTAGAGAAATCAGAA    8333
PP554504_Bat_2023_India         CTCTATTACATTGGGACGTAGTGTATTCAGATTGATTGAATTANNNNNNNNNNNNNNNNN    8276
JN808863_Hu_2008_Bangladesh     CTCTATTACATTGGGACGTAGTGTATTCAGATTGATTGAATTACATCAAAGAGATCAAAG    8339
                                ***** *********** ******************  *****

NC_002728_Nipah_Reference       AACTTCTGACTTTCAGAAATGGATTGTATACAATTAGTTAGATCATCCTGAATAATCGAG    8393
PP554504_Bat_2023_India         NNNNNNNNNNNNNNNNNNNNNNNNNNNNNNNNNNNNNNNNNNNNNNNNNNNNNNNNNNNN    8336
JN808863_Hu_2008_Bangladesh     GACTTCTAATTTTTAGAAATGGATTGTATGTAATTGATTAGATCATCTTGGATACTTGAG    8399

NC_002728_Nipah_Reference       GTGAGAACATTGCAACTATAAAATCAGATCATGTAAATAGTTGTAAAAAATTAAAAGCTT    8453
PP554504_Bat_2023_India         NNNNNNNNNNNNNNNNNNNNNNNNNNNNNNNNNNNNNNNNNNNNNNNNNNNNNNNNNNNN    8396
JN808863_Hu_2008_Bangladesh     ATAGGAATGTTGCAACTTTAAAATTAAACCATGTAAATAATTGTGACAAATTATAAGTTT    8459
```

```
NC_002728_Nipah_Reference        CTTTTAATTCTTTTGAACAATAATTTAATTAATATATAACATATTCTCTCACACGAGCGC    8513
PP554504_Bat_2023_India          NNNNNNNNNNNNNNNNNNNNNNNNNNNNNNNNNNNNNNNNNNNNNNNNNNNNNNNNNNNN    8456
JN808863_Hu_2008_Bangladesh      CTTTCATTTTCTTAAAGTAATAATTTGATGAATATACAATATATTCTCTCGCTAGAGCTC    8519


NC_002728_Nipah_Reference        TAACCTATACACTCTCTACTAATATTTTATACTCATAATTAATGATATAATGACAAATAA    8573
PP554504_Bat_2023_India          NNNNNNNNNNNNNNNNNNNNNNNNNNNNNNNNNNNNNNNNNNNNNNNNNNNNNNNNNNNN    8516
JN808863_Hu_2008_Bangladesh      TAATCTATACACTCCCTCCTAATATTTTATACTCATAATTAATGATATGATGATAAATAA    8579


NC_002728_Nipah_Reference        GGATTCAAATTGGATTATGATATAGTTTCATACTACAATAGCATTTCGACCAAGAAAATA    8633
PP554504_Bat_2023_India          NNNNNNNNNNNNNNNNNNNNNNNNNNNNNNNNNNNNNNNNNNNNNNNNNNNNNNNNNNNN    8576
JN808863_Hu_2008_Bangladesh      AGATTCAAATTGGCTTATGATATAATCTCATACTATAACAACATCTCAGCCAAGGAAATG    8639


NC_002728_Nipah_Reference        TCCTTACAATTATACAATGTACTTAACCGTGAATATGTAATTGATAATTTCCCTTTAGAA    8693
PP554504_Bat_2023_India          NNNNNNNNNNNNNNNNNNNNNNNNNNNNNNNNNNNNNNNNNNNNNNNNNNNNNNNNNNNN    8636
JN808863_Hu_2008_Bangladesh      TACTTGCAATTATACATTGTACTCAACCATGAATGAACAGTTGATATTCTTTTTTTGAAA    8699


NC_002728_Nipah_Reference        ATTTAATAAAAAACTTAGGACCCAGGTCCATAACTCATTGGATACTTAACTGTATCTTTC    8753
PP554504_Bat_2023_India          NNNNNATAAAAAACTTAGGACCCAGGTCCATAACTCATTGGATATTAAACTGTGTCCTTT    8696
JN808863_Hu_2008_Bangladesh      AAATTATAAAAAACTTAGGACCCAGGTCCATAACTCATTGGATATTAAACTGTGTCCTTT    8759
                                      *************************************** * ****** ** **

NC_002728_Nipah_Reference        TAAGCTATCACATATCAAAGGAGAGATTGAATGCTTTTTTGGAGATCTAGATCATTACTA    8813
PP554504_Bat_2023_India          GAAGTTATCATATATCAGGGGAGAAATAAAAGGTTTTTTTNNNNNNNNNNNNNNNNNNNN    8756
JN808863_Hu_2008_Bangladesh      GAAGTTATCATATATCAGGGGAGAAATAAAAGGTTTTTTTAGGTTTTTAGATCATCATAA    8819
                                  *** ***** ******  ***** **  ** * *****

NC_002728_Nipah_Reference        TATGTGTCTCCTATAATCACATCATAGGAGTGAACCATAATACACATCTTTGGGTAGGGG    8873
PP554504_Bat_2023_India          NNNNNNNNNNNNNNNNNNNNNNNNNNNNNNNNNNNNNNNNNNNNNNNNNNNNNNNNNNNN    8816
JN808863_Hu_2008_Bangladesh      TATGTATCTTCTTTAATCACATTATAGGAGTGAGCCACAATATACATCTTTGAGTAAAGA    8879


NC_002728_Nipah_Reference        AAGGAAAGTATTGTTGACGTACTGATTGATCTGCTTGAGTCAAATAATCAGTCATAACAA    8933
PP554504_Bat_2023_India          NNNNNNNNNNNNNNNNNNNNNNNNNNNNNNCTGCCTGAATTAAATAATCACCCAAAAAAA    8876
JN808863_Hu_2008_Bangladesh      GAAGAAAGCATTGCTGGCACACAAGCTGACCTGCCTTAATTAAATAATCACCCAAAAAAA    8939
                                                               **** * * * *********  ** ** **

NC_002728_Nipah_Reference        TTCAAGAAAATGCCGGCAGAAAACAAGAAAGTTAGATTCGAAAATACTACTTCAGACAAA    8993
PP554504_Bat_2023_India          TCAAAGAGGATGCCGACAGAAAGCAAGAAAGTTAGATTCGAAAATACTGCTTCAGACAAG    8936
JN808863_Hu_2008_Bangladesh      TCAAAGAGAATGCCGACAGAAAGCAAGAAAGTTAGATTTGAAAATACTGCTTCAGACAAG    8999
                                 *  ****  ****** ****** *************** ********* **********

NC_002728_Nipah_Reference        GGGAAAATTCCTAGTAAAGTTATTAAGAGCTACTACGGAACCATGGACATTAAGAAAATA    9053
PP554504_Bat_2023_India          GGGAAAAATCCTAGTAAAGTTATTAAGAGCTACTACGGAACCATGGACATTAAGAAAATA    8996
JN808863_Hu_2008_Bangladesh      GGGAAAAATCCTAGTAAAGTTATTAAGAGCTACTACGGAACCATGGACATTAAGAAAATA    9059
                                 ******* ****************************************************

NC_002728_Nipah_Reference        AATGAAGGATTATTGGACAGCAAAATATTAAGTGCTTTCAACACAGTAATAGCATTGCTT    9113
PP554504_Bat_2023_India          AATGAGGGGTTATTGGACAGCAAGATATTAAGTGCTTTCAACACAGTGATAGCACTGCTT    9056
JN808863_Hu_2008_Bangladesh      AATGAAGGGTTATTGGACAGCAAGATATTAAGTGCTTTCAACACAGTGATAGCACTGCTT    9119
                                 ***** ** ************** *********************** ****** *****

NC_002728_Nipah_Reference        GGATCTATCGTGATCATAGTGATGAATATAATGATCATCCAAAATTACACAAGATCAACA    9173
PP554504_Bat_2023_India          GGATCCATTGTAATCATAGTGATGAATATAATGATCATCCAGAACTACACAAGATCAACA    9116
JN808863_Hu_2008_Bangladesh      GGATCCATTGTAATCATAGTGATGAATATAATGATCATCCAGAACTACACAAGATCAACA    9179
                                 ***** ** ** ***************************** ** ***************

NC_002728_Nipah_Reference        GACAATCAGGCCGTGATCAAAGATGCGTTGCAGGGTATCCAACAGCAGATCAAAGGGCTT    9233
PP554504_Bat_2023_India          GATAATCAGGCCATGATCAAAGATGCATTGCAGAGTATCCAGCAGCAGATCAAGGGGCTT    9176
JN808863_Hu_2008_Bangladesh      GATAATCAGGCCATGATCAAAGATGCATTGCAGAGTATCCAGCAGCAGATCAAGGGGCTT    9239
                                 ** ********* ************* ****** ******* *********** ******

NC_002728_Nipah_Reference        GCTGACAAAATCGGCACAGAGATAGGGCCCAAAGTATCACTGATTGACACATCCAGTACC    9293
PP554504_Bat_2023_India          GCCGACAAAATTGGCACAGAGATAGGGCCGAAAGTATCACTGATTGATACATCCAGTACT    9236
JN808863_Hu_2008_Bangladesh      GCCGACAAAATTGGCACAGAGATAGGGCCGAAAGTATCACTGATTGATACATCCAGTACT    9299
                                 ** ******** ***************** ***************** ***********

NC_002728_Nipah_Reference        ATTACTATCCCAGCTAACATTGGGCTGTTAGGTTCAAAGATCAGCCAGTCGACTGCAAGT    9353
PP554504_Bat_2023_India          ATCACTATCCCAGCTAATATTGGGCTGTTAGGTTCAAAGATCAGCCAGTCAACTGCAAGT    9296
JN808863_Hu_2008_Bangladesh      ATCACTATCCCAGCTAATATTGGGCTGTTAGGTTCAAAGATCAGCCAGTCAACTGCAAGT    9359
                                 ** ************** ******************************** *********

NC_002728_Nipah_Reference        ATAAATGAGAATGTGAATGAAAAATGCAAATTCACACTGCCTCCCTTGAAAATCCACGAA    9413
PP554504_Bat_2023_India          ATAAATGAGAATGTAAATGAAAAATGCAAATTTACACTGCCTCCCTTGAAAATCCACGAA    9356
JN808863_Hu_2008_Bangladesh      ATAAATGAGAATGTGAATGAAAAATGCAAATTTACACTGCCTCCCTTGAAAATCCACGAA    9419
                                 ************** ***************** ***************************

NC_002728_Nipah_Reference        TGTAACATTTCTTGTCCTAACCCACTCCCTTTTAGAGAGTATAGGCCACAGACAGAAGGG    9473
PP554504_Bat_2023_India          TGTAACATTTCTTGTCCTAACCCACTCCCTTTTAGAGAGTATAAGCCGCAGACAGAAGGA    9416
JN808863_Hu_2008_Bangladesh      TGTAACATTTCTTGTCCTAACCCACTCCCTTTTAGAGAGTATAAGCCGCAGACAGAAGGA    9479
                                 ******************************************* *** ***********

NC_002728_Nipah_Reference        GTGAGCAATCTAGTAGGATTACCTAATAATATTTGCCTGCAAAAGACATCTAATCAGATA    9533
PP554504_Bat_2023_India          GTGAGCAATCTGGTAGGATTACCTAATAATATCTGTCTGCAAAAGACATCTAATCAGATA    9476
```

```
JN808863_Hu_2008_Bangladesh     GTGAGCAATCTGGTAGGATTACCTAATAATATCTGTCTGCAAAAGACATCTAATCAGATA     9539
                                *********** ******************** ** ************************

NC_002728_Nipah_Reference       TTGAAGCCAAAGCTGATTTCATACACTTTACCCGTAGTCGGTCAAAGTGGTACCTGTATC     9593
PP554504_Bat_2023_India         CTGAAACCAAAGCTGATTTCATACACCTTACCCGTAGTCGGTCAAAGTGGCACCTGTATC     9536
JN808863_Hu_2008_Bangladesh     CTGAAACCAAAGCTGATTTCATACACCTTACCCGTAGTCGGTCAAAGTGGCACCTGTATC     9599
                                 **** ******************** ************************ ********

NC_002728_Nipah_Reference       ACAGACCCATTGCTGGCTATGGACGAGGGCTATTTTGCATATAGCCACCTGGAAAGAATC     9653
PP554504_Bat_2023_India         ACAGACCCACTGCTGGCTATGGATGAGGGCTACTTTGCATATAGCCACCTGGAAAAAATC     9596
JN808863_Hu_2008_Bangladesh     ACAGACCCACTGCTGGCTATGGATGAGGGCTACTTTGCATATAGCCACCTGGAAAAAATC     9659
                                ********* ************* ******** ********************** ****

NC_002728_Nipah_Reference       GGATCATGTTCAAGAGGGGTCTCCAAACAAAGAATAATAGGAGTTGGAGAGGTACTAGAC     9713
PP554504_Bat_2023_India         GGATCATGTTCAAGAGGGGTCTCCAAACAAAGAATAATAGGAGTTGGAGAGGTACTAGAC     9656
JN808863_Hu_2008_Bangladesh     GGATCATGTTCAAGAGGGGTCTCCAAACAAAGAATAATAGGAGTTGGAGAGGTACTAGAC     9719
                                ************************************************************

NC_002728_Nipah_Reference       AGAGGTGATGAAGTTCCTTCTTTATTTATGACCAATGTCTGGACCCCACCAAATCCAAAC     9773
PP554504_Bat_2023_India         AGAGGTGACGAAGTACCTTCTTTGTTTATGACTAACGTCTGGACCCCATCAAATCCAAAC     9716
JN808863_Hu_2008_Bangladesh     AGAGGTGACGAAGTACCTTCTTTGTTTATGACTAACGTCTGGACCCCATCAAATCCAAAC     9779
                                ******** ***** ******** ******** ** ************ ***********

NC_002728_Nipah_Reference       ACCGTTTACCACTGTAGTGCTGTATACAACAATGAATTCTATTATGTACTTTGTGCAGTG     9833
PP554504_Bat_2023_India         ACCGTTTACCATTGCAGTGCCGTGTACAACAATGAATTCTATTATGTGCTTTGTGCAGTG     9776
JN808863_Hu_2008_Bangladesh     ACCGTTTACCATTGCAGTGCCGTGTACAACAATGAATTCTATTATGTGCTTTGTGCAGTG     9839
                                *********** ** ***** ** *********************** ************

NC_002728_Nipah_Reference       TCAACTGTTGGAGACCCTATTCTGAATAGCACCTACTGGTCCGGATCTCTAATGATGACC     9893
PP554504_Bat_2023_India         TCAGTTGTTGGAGACCCTATTCTGAATAGCACCTACTGGTCCGGATCACTAATGATGACT     9836
JN808863_Hu_2008_Bangladesh     TCAGTTGTTGGAGACCCTATTCTGAATAGCACCTACTGGTCCGGATCACTAATGATGACT     9899
                                ***  ****************************************** ***********

NC_002728_Nipah_Reference       CGTCTAGCTGTGAAACCCAAGAGTAATGGTGGGGGTTACAATCAACATCAACTTGCCCTA     9953
PP554504_Bat_2023_India         CGTCTAGCTGTAAAACCTAAGAATAATGGTGAGAGTTACAATCAACATCAATTTGCCTTA     9896
JN808863_Hu_2008_Bangladesh     CGTCTAGCTGTAAAACCTAAGAATAATGGTGAGAGTTACAATCAACATCAATTTGCCTTA     9959
                                *********** ***** **** ******** * ***************** ***** **

NC_002728_Nipah_Reference       CGAAGTATCGAGAAAGGGAGGTATGATAAAGTTATGCCGTATGGACCTTCAGGCATCAAA     10013
PP554504_Bat_2023_India         CGGAATATTGAGAAAGGGAAGTATGATAAAGTTATGCCATATGGACCCTCAGGCATCAAA     9956
JN808863_Hu_2008_Bangladesh     CGGAATATTGAGAAAGGGAAGTATGATAAAGTTATGCCATATGGACCCTCAGGCATCAAA     10019
                                ** * *** ********** ****************** ******** ************

NC_002728_Nipah_Reference       CAGGGTGACACCCTGTATTTTCCTGCTGTAGGATTTTTGGTCAGGACAGAGTTTAAATAC     10073
PP554504_Bat_2023_India         CAAGGTGACACCCTGTACTTCCCTGCTGTAGGTTTTTTGGTCAGGACAGAGTTCAAATAC     10016
JN808863_Hu_2008_Bangladesh     CAAGGTGACACCCTGTACTTTCCTGCTGTAGGTTTTTTGGTCAGGACAGAGTTCAAATAC     10079
                                ** ************** ** *********** ******************** ******

NC_002728_Nipah_Reference       AATGATTCAAATTGTCCCATCACGAAGTGTCAATACAGTAAACCTGAAAATTGCAGGCTA     10133
PP554504_Bat_2023_India         NNNNNNNNNNNNNNNNNNNNNNNNNNGTGTCAATACAGCAAACCTGAAAACTGCAGGCTA     10076
JN808863_Hu_2008_Bangladesh     AATGATTCAAATTGTCCCATCGCAGAGTGTCAATACAGCAAACCTGAAAACTGCAGGCTA     10139
                                                          ************ *********** *********

NC_002728_Nipah_Reference       TCTATGGGGATTAGACCAAACAGCCATTATATCCTTCGATCTGGACTATTAAAATACAAT     10193
PP554504_Bat_2023_India         TCTATGGGGATCAGACCAAATAGTCATTATATCCTTCGATCTGGACTACTAAAATACAAT     10136
JN808863_Hu_2008_Bangladesh     TCTATGGGGATTAGACCAAACAGTCATTATATCCTTCGATCTGGACTACTAAAATACAAT     10199
                                *********** ******** ** ************************ ***********

NC_002728_Nipah_Reference       CTATCAGATGGGGAGAACCCCAAAGTTGTATTCATTGAAATATCTGATCAAAGATTATCT     10253
PP554504_Bat_2023_India         CTATCGGATGAGGAGAACTCTAAAATTGTATTCATTGAAATATCTGATCAAAGACTATCT     10196
JN808863_Hu_2008_Bangladesh     CTATCGGATGAGGAGAACTCTAAAATTGTATTCATTGAAATATCTGATCAAAGACTATCT     10259
                                ***** **** ******* * *** ***************************** *****

NC_002728_Nipah_Reference       ATTGGATCTCCTAGCAAAATCTATGATTCTTTGGGTCAACCTGTTTTCTACCAAGCGTCA     10313
PP554504_Bat_2023_India         ATTGGATCTCCTAGCAAAATCTATGATTCTTTGGGTCAACCTGTTTTCTACCAAGCGTCT     10256
JN808863_Hu_2008_Bangladesh     ATTGGATCTCCTAGCAAAATCTATGATTCTTTGGGTCAACCTGTTTTCTACCAAGCGTCT     10319
                                ***********************************************************

NC_002728_Nipah_Reference       TTTTCATGGGATACTATGATTAAATTTGGAGATGTTCTAACAGTCAACCCTCTGGTTGTC     10373
PP554504_Bat_2023_India         TTTTCATGGGACACTATGATTAAATTCGGAGATGTCCAAACAGTTAACCCTTTAGTTGTA     10316
JN808863_Hu_2008_Bangladesh     TTTTCATGGGACACTATGATTAAATTTGGAGATGTCCAAACAGTTAACCCTTTAGTTGTA     10379
                                *********** ************** ******** * ****** ****** * *****

NC_002728_Nipah_Reference       AATTGGCGTAATAACACGGTAATATCAAGACCCGGGCAATCACAATGCCCTAGATTCAAT     10433
PP554504_Bat_2023_India         AATTGGCGTGACAACACGGTAATCTCAAGACCTGGGCAATCACAATGCCCTAGATTCAAC     10376
JN808863_Hu_2008_Bangladesh     AATTGGCGTGACAACACGGTAATCTCAAGACCTGGACAATCACAATGCCCTAGATTCAAC     10439
                                ********* * *********** ******** ** ***********************

NC_002728_Nipah_Reference       ACATGTCCAGAGATCTGCTGGGAAGGAGTTTATAATGATGCATTCCTAATTGACAGAATC     10493
PP554504_Bat_2023_India         AAGTGCCCAGAGGTTTGCTGGGAAGGGGTTTATAATGATGCCTTCCTGATTGATAGAATC     10436
JN808863_Hu_2008_Bangladesh     AAGTGCCCAGAGGTTTGCTGGGAAGGGGTTTATAATGATGCCTTCCTGATTGATAGAATC     10499
                                *  ** ****** * *********** ************** ***** ***** ******

NC_002728_Nipah_Reference       AATTGGATAAGCGCGGGTGTATTCCTTGACAGCAATCAGACCGCAGAAAATCCTGTTTTT     10553
PP554504_Bat_2023_India         AATTGGATAAGTGCGGGTGTATTCCTTGACAGCAACCAGACCGCAGAGAATCCTGTTTTT     10496
JN808863_Hu_2008_Bangladesh     AATTGGATAAGTGCGGGTGTATTCCTTGACAGCAACCAGACCGCAGAGAATCCTGTTTTT     10559
                                *********** *********************** *********** ************
```

```
NC_002728_Nipah_Reference       ACTGTATTCAAAGATAATGAAATACTTTATAGGGCACAACTGGCTTCTGAGGACACCAAT     10613
PP554504_Bat_2023_India         ACTGTATTCAAAGATAATGAAGTACTTTACAGAGCACAACTAGCCTCCGAGGACACCAAT     10556
JN808863_Hu_2008_Bangladesh     ACTGTATTCAAAGATAATGAAGTACTTTACAGAGCACAACTAGCCTCCGAGGACACCAAT     10619
                                ********************* ******* ** ******** ** ** ************

NC_002728_Nipah_Reference       GCACAAAAAACAATAACTAATTGTTTTCTCTTGAAGAATAAGATTTGGTGCATATCATTG     10673
PP554504_Bat_2023_India         GCACAAAAAACAATAACTAATTGCTTCCTTTTGAAGAATAAGATCTGGTGTATATCACTG     10616
JN808863_Hu_2008_Bangladesh     GCACAAAAAACAATAACTAATTGCTTCCTTTTGAAGAATAAGATCTGGTGTATATCACTG     10679
                                *********************** ** ** ************** ***** ****** **

NC_002728_Nipah_Reference       GTTGAGATATATGACACAGGAGACAATGTCATAAGACCCAAACTATTCGCGGTTAAGATA     10733
PP554504_Bat_2023_India         GTTGAGATATACGACACAGGAGACAATGTTATAAGACCTAAACTATTCGCAGTTAAGATA     10676
JN808863_Hu_2008_Bangladesh     GTTGAGATATACGACACAGGAGACAATGTTATAAGACCTAAACTATTCGCAGTTAAGATA     10739
                                *********** ***************** ******** *********** *********

NC_002728_Nipah_Reference       CCAGAGCAATGTACATAAAAATCAACCTCATAATTTAATGGATTGATCTAATATAATGAT     10793
PP554504_Bat_2023_India         CCAGAGCAATGTACATAAAAGTTAATCTCATAATTTGATGGAATGATCTNNNNNNNNNNN     10736
JN808863_Hu_2008_Bangladesh     CCAGAGCAATGTACATAAAAGTTAATCTCATAATTTGATGGAATGATCTGGTGCAATAAT     10799
                                ******************** * ** ********** ***** ******

NC_002728_Nipah_Reference       AATAATCGTACAAAGACATGTGATGTAAACAAAATTGTTGTAATTAAATAAGTCCTCAGC     10853
PP554504_Bat_2023_India         NNNNNNNNNNNNNNNNNNNNNNNNNNNNNNNNNNNNNNNNNNNNNNNNNNNNNNNNNNNN     10796
JN808863_Hu_2008_Bangladesh     AGCAACTGTGCAAAGGAATGTGATGTGAATAAAATTGTCATAATTACATAAGTCATCAGC     10859

NC_002728_Nipah_Reference       TGAATACTTTTTTAAGATTAGCAATAGCATGTTTTTCCAGTTATTGGATAGTTGATAATA     10913
PP554504_Bat_2023_India         NNNNNNNNNNNNNNNNNNNNNNNNNNNNNNNNNNNNNNNNNNNNNNNNNNNNNNNNNNNN     10856
JN808863_Hu_2008_Bangladesh     TGAATATTTTTTTAGGGGTAATAATAACATGTTTCTTCAATTATAGGATTACTGATAATG     10919

NC_002728_Nipah_Reference       TAATTCTGAAACTGGGTTAATAAATAATCTTGATCGGTGATCTTTGAGAACAATGATATC     10973
PP554504_Bat_2023_India         NNNNNNNNNNNNNNNNNNNNNNNNNNNNNNNNNNNNNNNNNNNNNNNNNNNNNNNNNNNN     10916
JN808863_Hu_2008_Bangladesh     TAATTCGGAGACCGGGTCAATAAATAATCTTGATCAATGATCTTTGAAAATAATGATAAC     10979

NC_002728_Nipah_Reference       ATATAGTTCATCAAGTGATAATCAATTCTTTATATGTACACTTTAGAGTATATTTTGAGA     11033
PP554504_Bat_2023_India         NNNNNNNNNNNNNNNNNNNNNNNNNNNNNNNNNNNNNNNNNNNNNNNNNNNNNNNNNNNN     10976
JN808863_Hu_2008_Bangladesh     ATGTCGTTCATCAATTGATAACCAACTCTTTCTATGTACACCTTGGAGCAAGTCTTGGGA     11039

NC_002728_Nipah_Reference       CTTAGTATTTTCGGCCCGAATGTTAAATTTAATAGTTCATACATAACCTAAACTCAAGTT     11093
PP554504_Bat_2023_India         NNNNNNNNNNNNNNNNNNNNNNNNNNNNNNNNNNNNNNNNNNNNNNNNNNNNNNNNNNNN     11036
JN808863_Hu_2008_Bangladesh     CTTAATATATTCGATTTGGATATCAGAGGTAACAGTCTATACATAATCTTAACTTACGCT     11099

NC_002728_Nipah_Reference       CTAAGCATAATGATAACAATTAATGCGAACTTGTCTTGATGTAAGGAAGATTTGATATTA     11153
PP554504_Bat_2023_India         NNNNNNNNNNNNNNNNNNNNNNNNNNNNNNNNNNNNNNNNNNNNNNNNNNNNNNNNNNNN     11096
JN808863_Hu_2008_Bangladesh     TCAAGCATAGTGATGACAATTAATGTGAACTTGTCTCAATGTAAAGAGAATTTGATCTTG     11159

NC_002728_Nipah_Reference       ACTGAGACTCCACTTGATATAGTAGAGCTGAATCTTGTAAATAAATTATAATGAATAGTT     11213
PP554504_Bat_2023_India         NNNNNNNNNNNNNNNNATATAGTAGAGCTAAATCTTGTAAATAAATTATAATAAATGGTT     11156
JN808863_Hu_2008_Bangladesh     ACTGAAACTCCATTTGATATAGTAGAGCTAAATCTTGTAAATAAATTATAATAAATGGTT     11219
                                                ************* ********************** *** ***

NC_002728_Nipah_Reference       TATTCAAAGATTATCATTCATATTAGTGTAAATTAAGAAAAACTTAGGACCCAGGTCCTT     11273
PP554504_Bat_2023_India         TATTCAAAGATTATCGTTTATATTAATGTAAATTAAGAAAAACTTAGGACCCAGGTCCTT     11216
JN808863_Hu_2008_Bangladesh     TATTCAAAGATTATCGTTTATATTAATGTAAATTAAGAAAAACTTAGGACCCAGGTCCTT     11279
                                *************** ** ****** **********************************

NC_002728_Nipah_Reference       GATTATGCCAATTTTCTCGAGAAATCATTCAATTGACCATAGACTGAAAGCGTTGTTACC     11333
PP554504_Bat_2023_India         GATTGTGCTAATTTTCTTGAGAAATCATTTAATTNNNNNNNNNCTGAAGACGTTATTACC     11276
JN808863_Hu_2008_Bangladesh     GATTGTGCTAATTTTCTTGAGAAATCATTTAATTTCCTACAGACTGAAGACGTTATTACC     11339
                                **** *** ******** *********** ****         *****  **** *****

NC_002728_Nipah_Reference       TAGTTCTTCAGAAGAGATCTTATTAGAATTAATTTATATGATCTAATTCCCTTAAAAACT     11393
PP554504_Bat_2023_India         TATTTTTTTAGGGGAAATCTCATTAGAATTGATCTATATTATCTAATTCTCCTAAAAATT     11336
JN808863_Hu_2008_Bangladesh     TATTTTTTTAGGGGAAATCTTATTAGAATTGATCTATATTATCTAATTCTCCTAAAAATT     11399
                                ** ** ** **  ** **** ********* ** ***** ********* * ****** *

NC_002728_Nipah_Reference       GAATACCAAAAAACAAAAATGGCCGATGAATTATCAATATCCGACATCATTTACCCTGAA     11453
PP554504_Bat_2023_India         GGCTACCAAAAAACAAAGATGGCCGATGAATTATCCATATCTGACATCATCTACCCTGAA     11396
JN808863_Hu_2008_Bangladesh     GACTACCAAAAAACAAAGATGGCCGATGAATTATCCATATCTGACATCATCTACCCTGAA     11459
                                *  ************** ***************** ***** ******** *********

NC_002728_Nipah_Reference       TGTCATTTGGATAGTCCTATAGTCTCTGGTAAACTAATATCAGCTATTGAATATGCTCAA     11513
PP554504_Bat_2023_India         TGTCATTTGGATAGTCCTATAGTCTCTGGTAAACTAATATAAGCTATTGAATATGCTCAA     11456
JN808863_Hu_2008_Bangladesh     TGTCATTTGGATAGTCCTATAGTCTCTGGTAAACTAATATCAGCTATTGAATATGCTCAA     11519
                                **************************************** *******************

NC_002728_Nipah_Reference       TTGAGACACAATCAGCCCAGTGATGATAAAAGACTGTCTGAGAATATTAGGTTAAACCTT     11573
PP554504_Bat_2023_India         TTGAAACACAATCAACCTAGTGATGACAAAAGACTGTCTGAAAATATTAGGTTAAACCTT     11516
JN808863_Hu_2008_Bangladesh     TTGAAACACAATCAACCTAGTGATGACAAAAGACTGTCTGAAAATATTAGGTTAAACCTT     11579
                                **** ********* ** ******** ************** ******************

NC_002728_Nipah_Reference       CACGGGAAAAGAAAGAGTCTATACATATTAAGACAATCCAAACAGGGTGATTACATTAGA     11633
PP554504_Bat_2023_India         CACGGGAAAAGAAAGAGNNNNNNNNNNNNNNNNNNNNNNNNNNNNNNNNNNNNNNNNNNN     11576
JN808863_Hu_2008_Bangladesh     CACGGGAAAAGAAAGAGCTTATATATATTAAGACAATCCAAGCAAGGTAATTATATCAGA     11639
```

```
                                *****************

NC_002728_Nipah_Reference       AACAACATAAAAAACCTAAAGGAATTCATGCATATTGCGTACCCTGAATGCAATAACATT      11693
PP554504_Bat_2023_India         NNNNNNNNNNNNNNNNNNNNNNNNNNNNNNNNNNNNNNNNNNNNNNNNNNNNNNNACATT      11636
JN808863_Hu_2008_Bangladesh     AATAACGTTAAAAACTTAAAAGAGTTCATGCATATTGCGTACCCTGAATGTAATAACATT      11699
                                                                                       *****

NC_002728_Nipah_Reference       CTATTCTCCATCACATCCCAAGGCATGACTAGCAAACTTGATAACATCATGAAAAAGTCA      11753
PP554504_Bat_2023_India         TTATTTTCCATTACATCCCAAGGCATGACTAGCAAACTTGATAACATCATGAAGAAGTCA      11696
JN808863_Hu_2008_Bangladesh     TTATTTTCCATTACATCCCAAGGCATGACTAGCAAACTTGATAACATCATGAAGAAGTCA      11759
                                 **** ***** ***************************************** ******

NC_002728_Nipah_Reference       TTCAAAGCATACAATATCATTAGTAAGAAAGTAATTGGGATGCTGCAAAATATCACTAGA      11813
PP554504_Bat_2023_India         TTCAAAGCATACAATATCATTAGTAAGAAAGTAATCGGAATGCTACAAAACATCACTAGA      11756
JN808863_Hu_2008_Bangladesh     TTCAAAGCATACAATATCATTAGTAAGAAAGTAATCGGAATGCTACAAAACATCACTAGA      11819
                                *********************************** ** ***** ***** *********

NC_002728_Nipah_Reference       AATCTCATAACTCAAGATAGAAGAGATGAAATAATTAATATACATGAGTGTAGGCGATTA      11873
PP554504_Bat_2023_India         AATCTCATAACCCAAGACAGAAGGGATGAAATAATCAATATACATGAGTGTNNNNNNNNN      11816
JN808863_Hu_2008_Bangladesh     AATCTCATAACCCAAGATAGAAGGGATGAAATAATCAATATACATGAGTGTAGACGGTTA      11879
                                *********** ***** ***** *********** ***************

NC_002728_Nipah_Reference       GGGGATTTAGGGAAGAATATGAGTCAATCTAAATGGTATGAGTGTTTTTTGTTTTGGTTT      11933
PP554504_Bat_2023_India         NNNNNNNNNNNNNNGAATATGAGTCAATCTAAATGGTATGAATGTTTTTTGTTTTGGTTT      11876
JN808863_Hu_2008_Bangladesh     GGAGATTTAGGGAAGAATATGAGTCAATCTAAATGGTATGAATGTTTTTTGTTTTGGTTT      11939
                                              *************************** ******************

NC_002728_Nipah_Reference       ACTATCAAAACAGAGATGCGAGCAGTGATCAAGAATTCGCAAAAGCCGAAATTCCGTTCA      11993
PP554504_Bat_2023_India         ACTATCAAAACAGAGATGCGAGCAGTGATCAAGAATTCACAAAAGCCGAAATTCCGTTCA      11936
JN808863_Hu_2008_Bangladesh     ACTATCAAAACAGAGATGCGAGCAGTGATCAAGAATTCACAAAAGCCGAAATTCCGTTCA      11999
                                ************************************** *********************

NC_002728_Nipah_Reference       GATTCATGCATAATACACATGCGAGACAAAAGTACTGAAATAATCCTAAATCCGAATCTT      12053
PP554504_Bat_2023_India         GATTCATGCATAATACATATGCGAGATAAAAGTACTGAAATAATCCTGAATCCAAATCTT      11996
JN808863_Hu_2008_Bangladesh     GATTCATGCATAATACATATGCGAGATAAAAGTACTGAGATAATCCTGAATCCAAATCTT      12059
                                ***************** ******** *********** ******** ***** ******

NC_002728_Nipah_Reference       ATCTGCATTTTCAAATCAGACAAAACTGGAAAGAAGTGTTATTATCTTACACCCGAAATG      12113
PP554504_Bat_2023_India         ATCTGCATTTTCAAATCAGACAAAACTGGAAAGAAGTGTTATTATCTTACACCCGAAATG      12056
JN808863_Hu_2008_Bangladesh     ATCTGCATTTTCAAATCAGACAAAACTGGAAAGAAGTGTTATTATCTTACACCCGAAATG      12119
                                ************************************************************

NC_002728_Nipah_Reference       GTTCTAATGTATTGTGATGTCCTAGAGGGAAGGATGATGATGGAGACAACAGTCAAATCG      12173
PP554504_Bat_2023_India         GTTCTAATGTATTGTGATGTCCTAGANNNNNNNNNNNNNNNNNNNNNNNNNNNNNNNNNN      12116
JN808863_Hu_2008_Bangladesh     GTTCTAATGTATTGTGATGTCCTAGAAGGGAGGATGATGATGGAGACAACAATCAAATCA      12179
                                **************************

NC_002728_Nipah_Reference       GATATCAAGTACCAACCTCTAATCTCGAGATCCAATGCCCTCTGGGGGCTAATTGATCCC      12233
PP554504_Bat_2023_India         NNNNTCAAATACCAGCCTTTAATCTCGAGGTCCAATGCCCTCTGGGGATTAATTGATCCC      12176
JN808863_Hu_2008_Bangladesh     GATATCAAATACCAGCCTTTAATCTCGAGGTCCAATGCCCTCTGGGGATTAATTGATCCC      12239
                                    **** ***** *** ********** *****************  ***********

NC_002728_Nipah_Reference       TTGTTCCCTGTCATGGGAAACAGAATTTACAATATAGTGTCTATGATAGAGCCTTTAGTT      12293
PP554504_Bat_2023_India         TTGTTCCCTGTCATGGGAAACAGGATTTACAATATAGTGTCTATGATAGAACCCTTAGTT      12236
JN808863_Hu_2008_Bangladesh     TTGTTCCCTGTCATGGGAAACAGGATTTACAATATAGTGTCTATGATAGAACCCTTAGTT      12299
                                *********************** ************************** ** ******

NC_002728_Nipah_Reference       CTTGCACTACTCCAACTCAAGGATGAGGCTAGGATCCTGAGGGGTGCATTTCTGCATCAC      12353
PP554504_Bat_2023_India         CTTGCACTACTCCAACTCAAGGACGAAGCTAGAATCCTGAGGGGTGCATTCCTACATCAC      12296
JN808863_Hu_2008_Bangladesh     CTTGCACTACTCCAACTCAAGGACGAAGCTAGAATCCTGAGGGGTGCATTCCTACATCAC      12359
                                *********************** ** ***** ***************** ** ******

NC_002728_Nipah_Reference       TGCATAAAGGAAATGCATCAAGAATTGAGTGAGTGTGGTTTTACAGATCAGAAGATTCGG      12413
PP554504_Bat_2023_India         TGCATAAAGGAGATGCATCAGGAACTGAGTGAGTGTGGCTTTACAGATCAGAAGATTCGG      12356
JN808863_Hu_2008_Bangladesh     TGCATAAAGGAGATGCATCAGGAACTGAGTGAGTGTGGCTTTACAGATCAGAAGATTCGG      12419
                                *********** ******** *** ************* *********************

NC_002728_Nipah_Reference       TCTATGTTTATTGATGATCTTTTATCCATTCTAAATATCGATAATATACATCTGTTGGCA      12473
PP554504_Bat_2023_India         TCTATGTTTATTGATGATCTTTTATCCATCCTGAATATCGACAATATACATCTATTGGCA      12416
JN808863_Hu_2008_Bangladesh     TCTATGTTTATTGATGACCTTTTATCCATCCTGAATATCGACAATATACATCTATTGGCA      12479
                                ***************** *********** ** ******** *********** ******

NC_002728_Nipah_Reference       GAGTTCTTTTCTTTCTTTCGTACGTTTGGCCATCCTATTCTTGAGGCTAAAGTTGCTGCA      12533
PP554504_Bat_2023_India         GAGTTCTTTTCTTTCTTTCGTACGTTTGGTCATCCTATTCTTGAGGCTAAAGTTGCTGCA      12476
JN808863_Hu_2008_Bangladesh     GAGTTCTTTTCTTTCTTTCGTACGTTTGGTCATCCTATTCTTGAGGCTAAAGTTGCTGCA      12539
                                ***************************** ******************************

NC_002728_Nipah_Reference       GAAAAAGTGAGAGAACATATGTTGGCAGATAAAGTTCTTGAATATGCCCCTATAATGAAA      12593
PP554504_Bat_2023_India         GAAAAAGTACGAGAACATATGTTAGCAGATAAAGTTCTTGAATATGCCCCTATAATGAAG      12536
JN808863_Hu_2008_Bangladesh     GAAAAAGTACGAGAACATATGTTAGCAGATAAAGTTCTTGAATATGCCCCTATAATGAAG      12599
                                ********  ************* ***********************************

NC_002728_Nipah_Reference       GCACATGCTATATTCTGCGGGACTATAATAAATGGGTATAGGGATAGACACGGAGGAGCC      12653
PP554504_Bat_2023_India         GCACATGCTATATTCTGCGGGACTATAATAAACGGGTATAGGGATAGACACGGAGGGGCC      12596
JN808863_Hu_2008_Bangladesh     GCACATGCTATATTCTGCGGGACTATAATAAACGGGTATAGGGATAGACACGGAGGGGCC      12659
                                ******************************** *********************** ***

NC_002728_Nipah_Reference       TGGCCTCCTCTTTACCTCCCCGCACATGCATCTAAACATATAATCCGTTTGAAAAATTCT      12713
```

```
PP554504_Bat_2023_India           TGGCCTCCTCTTTACCTCCCCGCACATGCATCTAAGCATATAATCCGCTTGAAAAATTCT     12656
JN808863_Hu_2008_Bangladesh       TGGCCTCCTCTTTACCTCCCCGCACATGCATCTAAGCATATAATCCGCTTGAAAAATTCT     12719
                                  *********************************** *********** ************

NC_002728_Nipah_Reference         GGGGAATCTTTGACCATTGATGACTGTGTCAAGAATTGGGAATCATTCTGTGGGATTCAA     12773
PP554504_Bat_2023_India           GGGGAATCTTTAACCATTGATGATTGTGTCAAGAACTGGGAATCATTCTGTGGGATTCAA     12716
JN808863_Hu_2008_Bangladesh       GGGGAATCTTTAACCATTGATGATTGTGTCAAGAACTGGGAATCATTCTGTGGGATTCAA     12779
                                  *********** *********** *********** ************************

NC_002728_Nipah_Reference         TTTGATTGTTTCATGGAGCTGAAATTGGACAGTGATCTGAGTATGTATATGAAAGATAAA     12833
PP554504_Bat_2023_India           TTTGATTGTTTCATGGAGCTGAAATTAGACAGTGATTTGAGTATGTATATGAAAGATAAA     12776
JN808863_Hu_2008_Bangladesh       TTTGATTGTTTCATGGAGCTGAAATTAGACAGTGATTTGAGTATGTATATGAAAGATAAA     12839
                                  ************************** ********* ***********************

NC_002728_Nipah_Reference         GCTTTATCTCCAATCAAAGACGAATGGGACAGTGTATACCCACGTGAAGTGTTGAGCTAT     12893
PP554504_Bat_2023_India           GCCTTGTCTCCTATCAAAGACGAGTGGGACAGTGTGTATCCACGAGAAGTGCTGAGCTAT     12836
JN808863_Hu_2008_Bangladesh       GCCTTGTCTCCTATCAAAGACGAGTGGGACAGTGTGTATCCACGAGAAGTGCTGAGCTAT     12899
                                  ** ** ***** *********** *********** ** ***** ****** ********

NC_002728_Nipah_Reference         ACCCCACCGAAGTCAACCGAGCCAAGAAGATTGGTTGACGTTTTTGTAAATGATGAAAAC     12953
PP554504_Bat_2023_India           ACCCCACCGAAATCAACCGAGCCAAGAAGATTGGTTGACGTTTTTGTAAACGATGAAAAC     12896
JN808863_Hu_2008_Bangladesh       ACCCCACCGAAATCAACCGAGCCAAGAAGATTGGTTGACGTTTTTGTAAACGATGAAAAC     12959
                                  *********** ************************************** *********

NC_002728_Nipah_Reference         TTTGATCCATACAACATGCTGGAATATGTCTTATCCGGTGCTTATCTCGAGGATGAACAA     13013
PP554504_Bat_2023_India           TTTGATCCATACAACATGCTAGAATATGTCTTGTCCGGCGCTTATCTCGAAGATGATCAA     12956
JN808863_Hu_2008_Bangladesh       TTTGATCCATACAACATGCTAGAATATGTCTTGTCCGGCGCTTATCTCGAAGATGATCAA     13019
                                  ******************** *********** ***** *********** ***** ***

NC_002728_Nipah_Reference         TTCAATGTTTCTTATAGCTTGAAGGAGAAAGAGACGAAGCAAGCTGGACGATTGTTCGCA     13073
PP554504_Bat_2023_India           TTCAATGTTTCTTACAGTTTGAAGGAGAAAGAGACAAAGCAGGCTGGACGATTGTTTGCG     13016
JN808863_Hu_2008_Bangladesh       TTCAATGTTTCTTACAGTTTGAAGGAGAAAGAGACAAAGCAGGCTGGACGATTGTTTGCG     13079
                                  ************** ** ***************** ***** ************** **

NC_002728_Nipah_Reference         AAGATGACCTACAAAATGCGTGCATGTCAAGTCATAGCAGAGGCCCTGATAGCCTCAGGT     13133
PP554504_Bat_2023_India           AAGATGACCTACAAGATGCGTGCATGTCAAGTTATAGCAGAAGCCCTAATAGCATCAGGT     13076
JN808863_Hu_2008_Bangladesh       AAGATGACCTACAAGATGCGTGCATGTCAAGTTATAGCAGAAGCCCTAATAGCATCAGGT     13139
                                  ************** ***************** ******** ***** ***** ******

NC_002728_Nipah_Reference         GTCGGTAAATATTTTAAGGAGAACGGGATGGTTAAGGATGAGCACGAACTTTTGAAGACA     13193
PP554504_Bat_2023_India           GTAGGTAAATATTTCAAGGAGAACGGGATGGTTAAGGATGAGCACGAACTTTTGAAGACA     13136
JN808863_Hu_2008_Bangladesh       GTAGGTAAATATTTCAAGGAGAACGGGATGGTTAAGGATGAGCACGAACTTTTGAAGACA     13199
                                  ** *********** *********************************************

NC_002728_Nipah_Reference         CTCTTCCAATTGTCTATTTCCTCAGTTCCTCGAGGGAACAGTCAGGGTAATGATCCTCAA     13253
PP554504_Bat_2023_India           CTCTTCCAATTATCCATTTCCTCAGTTCCTCGTGGAAACAGTCAGGGTAATGATCCTCAA     13196
JN808863_Hu_2008_Bangladesh       CTCTTCCAATTATCCATTTCCTCAGTTCCTCGTGGAAACAGTCAGGGTAATGATCCTCAA     13259
                                  *********** ** ***************** ** ************************

NC_002728_Nipah_Reference         TCCATCAATAATATAGAAAGAGATTTCCAATACTTTAAAGGGGTCACTACCAATGTGAAA     13313
PP554504_Bat_2023_India           TCCATCAATAATATAGAGAAAGATTTCCAATGCTTTAAAGGAGTTACCACCAATGTGAAA     13256
JN808863_Hu_2008_Bangladesh       TCCATCAATAATATAGAGAAAGATTTCCAATGCTTTAAAGGAGTTACCACCAATGTGAAA     13319
                                  ***************** * *********** ********* ** ** ************

NC_002728_Nipah_Reference         GACAAAAAGAATAACTCTTTTAATAAGGTTAAATCTGCTCTCAATAATCCGTGCCAAGCT     13373
PP554504_Bat_2023_India           GACAAAAAGAATAACCCTTTTTATAAAGTTAAATCTGCTCTCAATAATCCGTGCCAAGCT     13316
JN808863_Hu_2008_Bangladesh       GACAAAAAGAATAACCCTTTTTATAAAGTTAAATCTGCTCTCAATAATCCGTGCCAAGCT     13379
                                  *************** ***** **** *********************************

NC_002728_Nipah_Reference         GACGGAGTCCATCATAACATGTCACCCAATACACGAAATCGTTATAAGTGTAGTAATACA     13433
PP554504_Bat_2023_India           GACGGGGTCTATCATAACATGTCACCCAACACACGAAATCGTTATAAGTGTAGTAATACA     13376
JN808863_Hu_2008_Bangladesh       GACGGGGTCTATCATAACATGTCACCCAACACACGAAATCGTTATAAGTGTAGTAATACA     13439
                                  ***** *** ******************* ******************************

NC_002728_Nipah_Reference         AGTAAGTCTTTTCTCGATTATCATACCGAGTTTAATCCTCACAATCACTATAAATCAGAC     13493
PP554504_Bat_2023_India           AGTAAATCTTTTCTCGATTACCACACTGAGTTTAATCCCCACAATCACTACAAATCAGAC     13436
JN808863_Hu_2008_Bangladesh       AGTAAATCTTTTCTCGATTACCACACTGAGTTTAATCCCCACAATCACTACAAATCAGAC     13499
                                  ***** ************** ** ** *********** *********** *********

NC_002728_Nipah_Reference         AATACAGAGGCGGCCGTACTGTCCAGGTATGAGGACAACACTGGGACAAAATTTGATACA     13553
PP554504_Bat_2023_India           AATACGGAGGCAGCTGTACTCTCCAAGTATGAAGATAATACCGGGACAAAATTTGATACA     13496
JN808863_Hu_2008_Bangladesh       AATACGGAGGCAGCTGTACTCTCCAAGTATGAAGATAATACCGGGACAAAATTTGATACA     13559
                                  ***** ***** ** ***** **** ****** ** ** ** ******************

NC_002728_Nipah_Reference         GTAAGTGCATTTCTTACAACTGATCTTAAGAAATTCTGTCTCAATTGGAGATACGAATCA     13613
PP554504_Bat_2023_India           GTAAGTGCATTTCTTACAACTGATCTTAAGAAATTCTGTCTCAATTGGAGATACGAGTCA     13556
JN808863_Hu_2008_Bangladesh       GTAAGTGCATTTCTTACAACTGATCTTAAGAAATTCTGTCTCAATTGGAGATACGAGTCA     13619
                                  ******************************************************** ***

NC_002728_Nipah_Reference         ATGGCTATATTTGCTGAACGTCTGGATGAGATATACGGTTTACCTGGATTTTTTAATTGG     13673
PP554504_Bat_2023_India           ATGGCCATATTTGCTGAACGTCTAGATGAGATATACGGTTTACCTGGATTCTTTAATTGG     13616
JN808863_Hu_2008_Bangladesh       ATGGCCATATTTGCTGAACGTCTAGATGAGATATACGGTTTACCTGGATTCTTTAATTGG     13679
                                  ***** ***************** ************************** *********

NC_002728_Nipah_Reference         ATGCACAAACGACTAGAAAGATCTGTTATCTATGTTGCAGACCCTAATTGCCCCCCTAAT     13733
PP554504_Bat_2023_India           ATGCATAAACGACTAGAAAGGTCAGTTATCTATGTTGCGGATCCTAATTGCCCCCCTAAT     13676
JN808863_Hu_2008_Bangladesh       ATGCATAAACGACTAGAAAGGTCAGTTATCTATGTTGCGGATCCTAATTGCCCCCCTAAT     13739
                                  ***** ************** ** ************** ** ******************
```

```
NC_002728_Nipah_Reference       ATTGACAAACATATGGAACTAGAAAAAACTCCTGAAGATGATATATTCATTCATTATCCT    13793
PP554504_Bat_2023_India         ATTGACAAACACATGGAGTTAGAAGAAACTCCTGAAGATGATATATTCATTCATTATCCT    13736
JN808863_Hu_2008_Bangladesh     ATTGACAAACACATGGAGTTAGAAGAAACTCCTGAAGATGATATATTCATTCATTATCCT    13799
                                *********** *****  ***** ***********************************

NC_002728_Nipah_Reference       AAAGGCGGTATTGAAGGATATAGCCAAAAAACATGGACTATAGCAACTATCCCCTTTTTA    13853
PP554504_Bat_2023_India         AAAGGCGGTATTGAAGGATATAGCCAAAAAACATGGACTATAGCAACCATCCCCTTCTTA    13796
JN808863_Hu_2008_Bangladesh     AAAGGCGGTATTGAAGGATATAGCCAAAAAACATGGACTATAGCAACCATCCCCTTCTTA    13859
                                *********************************************** ******** ***

NC_002728_Nipah_Reference       TTCTTGAGTGCCTATGAGACAAACACGAGGATTGCTGCAATTGTCCAAGGAGACAATGAA    13913
PP554504_Bat_2023_India         TTCTTGAGTGCCTATGAGACAAACACGAGGATTGCTGCAATTGTCCAAGGAGACAACGAA    13856
JN808863_Hu_2008_Bangladesh     TTCTTGAGTGCCTATGAGACAAACACGAGGATTGCTGCAATTGTCCAAGGAGACAACGAA    13919
                                ******************************************************** ***

NC_002728_Nipah_Reference       TCAATTGCTATCACTCAAAAAGTTCATCCTAATCTTCCCTACAAGGTAAAGAAAGAGATC    13973
PP554504_Bat_2023_India         TCAATTGCTATCACTCAAAAAGTTCACCCCAATCTTCCCTACAAGGTTAAGAAAGAGATC    13916
JN808863_Hu_2008_Bangladesh     TCAATTGCTATCACTCAAAAAGTTCACCCCAATCTTCCCTACAAGGTTAAGAAAGAGATC    13979
                                ************************** ** ***************** ************

NC_002728_Nipah_Reference       TGTGCAAAGCAAGCTCAGCTTTATTTTGAAAGGTTAAGGATGAACTTAAGAGCCCTCGGC    14033
PP554504_Bat_2023_India         TGTGCAAAACAAGCTCAGCTTTACTTTGAAAGGTTAAGGATGAACTTAAGAGCTCTCGGC    13976
JN808863_Hu_2008_Bangladesh     TGTGCAAAACAAGCTCAGCTTTACTTTGAAAGGTTAAGGATGAACTTAAGAGCTCTCGGC    14039
                                ******** ************** ***************************** ******

NC_002728_Nipah_Reference       CACAATCTTAAAGCTACAGAAACTATCATCAGTACACATCTTTTTATTTATTCGAAGAAA    14093
PP554504_Bat_2023_India         CACAACCTTAAAGCTACCGAAACCATCATCAGTACACATCTTTTTGTTTATTCGAAGAAA    14036
JN808863_Hu_2008_Bangladesh     CACAACCTTAAAGCTACCGAAACCATCATCAGTACACATCTTTTTGTTTATTCGAAGAAA    14099
                                ***** *********** ***** ********************* **************

NC_002728_Nipah_Reference       ATTCATTATGATGGTGCTGTGCTGTCTCAGGCACTCAAATCAATGTCAAGATGTTGCTTT    14153
PP554504_Bat_2023_India         ATTCATTATGATGGTGCTGTGTTGTCCCAGGCACTCAAGTCAATGTCAAGATGCTGCTTT    14096
JN808863_Hu_2008_Bangladesh     ATTCATTATGATGGTGCTGTGTTGTCCCAGGCACTCAAGTCAATGTCAAGATGCTGCTTT    14159
                                ********************* **** *********** ************** ******

NC_002728_Nipah_Reference       TGGTCAGAGACTCTGGTGGATGAAACTAGATCAGCTTGTAGTAACATCAGCACTACAATA    14213
PP554504_Bat_2023_India         TGGTCAGAGACCCTGGTGGATGAAACTAGATCGGCTTGTAGTAACATCAGCACTACAATA    14156
JN808863_Hu_2008_Bangladesh     TGGTCAGAGACCCTGGTGGATGAAACTAGATCGGCTTGTAGTAACATCAGCACTACAATA    14219
                                *********** ******************** ***************************

NC_002728_Nipah_Reference       GCTAAAGCTATAGAAAATGGGTTGTCAAGAAATGTCGGCTATTGCATCAATATTTTGAAA    14273
PP554504_Bat_2023_India         GCTAAAGCTATAGAAAATGGGTTATCAAGAAATGTCGGCTATTGTATCAATATTCTGAAA    14216
JN808863_Hu_2008_Bangladesh     GCTAAAGCTATAGAAAATGGGTTATCAAGAAATGTCGGCTATTGTATCAATATTCTGAAA    14279
                                *********************** ******************** ********* *****

NC_002728_Nipah_Reference       GTAATTCAGCAGCTTCTCATATCAACTGAGTTTAGTATTAACGAGACATTGACACTGGAT    14333
PP554504_Bat_2023_India         GTAATTCAGCAACTCCTTATATCAACTGAGTTTAGTATTAATGAGACATTGACACTGGAT    14276
JN808863_Hu_2008_Bangladesh     GTAATTCAACAACTCCTTATATCAACTGAGTTTAGTATTAATGAGACATTGACACTGGAT    14339
                                ******** ** ** ** *********************** ******************

NC_002728_Nipah_Reference       GTGACATCTCCCATTTCAAATAATTTAGATTGGCTTATAACAGCTGCATTAATCCCGGCA    14393
PP554504_Bat_2023_India         GTGACATCTCCCATTTCAAATAATTTAGATTGGCTCATAACAGCAGCATTAATCCCAGCA    14336
JN808863_Hu_2008_Bangladesh     GTGACATCTCCCATTTCAAATAATTTAGATTGGCTCATAACAGCAGCATTAATCCCAGCA    14399
                                *********************************** ******** *********** ***

NC_002728_Nipah_Reference       CCTATTGGAGGATTCAATTACCTTAATTTGTCTAGAATTTTTGTTAGAAATATAGGTGAT    14453
PP554504_Bat_2023_India         CCTATCGGGGGATTTAATTACCTTAATTTGTCTAGAATTTTTGTTAGGAATATAGGTGAT    14396
JN808863_Hu_2008_Bangladesh     CCTATCGGGGGATTTAATTACCTTAATTTGTCTAGAATTTTTGTTAGGAATATAGGTGAT    14459
                                ***** ** ***** ******************************** ************

NC_002728_Nipah_Reference       CCGGTTACAGCATCTTTGGCTGATCTTAAGAGAATGATTGATCACAGTATTATGACTGAA    14513
PP554504_Bat_2023_India         CCGGTCACAGCATCTTTGGCTGATCTCAAAAGAATGATTGACCACAGCATTATGACTGAA    14456
JN808863_Hu_2008_Bangladesh     CCGGTCACAGCATCTTTGGCTGATCTCAAAAGAATGATTGACCACAGCATTATGACTGAA    14519
                                ***** ******************** ** *********** ***** ************

NC_002728_Nipah_Reference       AGCGTATTACAAAAAGTTATGAATCAAGAACCTGGTGATGCGAGTTTCTTGGACTGGGCC    14573
PP554504_Bat_2023_India         AGCGTATTACAAAAAGTTATGAATCAAGAACCTGGTGATGCAAGTTTCTTGGACTGGGCC    14516
JN808863_Hu_2008_Bangladesh     AGCGTATTACAAAAAGTTATGAATCAAGAACCTGGTGATGCAAGTTTCTTGGACTGGGCC    14579
                                ***************************************** ******************

NC_002728_Nipah_Reference       AGTGATCCATACTCGGGCAACTTGCCTGACTCACAAAGCATCACTAAAACAATTAAAAAT    14633
PP554504_Bat_2023_India         AGTGATCCATACTCAGGTAACTTGCCTGACTCGCAAAGTATTACTAAAACCATTAAAAAT    14576
JN808863_Hu_2008_Bangladesh     AGTGATCCATACTCAGGTAACTTGCCTGACTCGCAAAGTATTACTAAAACCATTAAAAAT    14639
                                ************** ** ************** ***** ** ******** *********

NC_002728_Nipah_Reference       ATCACAGCAAGGACTATACTGAGGAACTCACCGAACCCAATGCTAAAAGGTTTATTTCAT    14693
PP554504_Bat_2023_India         ATCACAGCAAGGACTATACTGAGGAACTCACCGAATCCAATGCTTAAAGGTCTATTTCAT    14636
JN808863_Hu_2008_Bangladesh     ATCACAGCAAGGACTATACTGAGGAACTCACCGAATCCAATGCTTAAAGGTCTATTTCAT    14699
                                *********************************** ******** ****** ********

NC_002728_Nipah_Reference       GACAAATCTTTTGATGAAGATCTTGAACTAGCTAGCTTCTTAATGGACAGGAGGGTTATA    14753
PP554504_Bat_2023_India         GACAAATCTTTTGACGAGGACCTTGAACTAGCTAGCTTCCTGATGGACAGGAGGGTGATA    14696
JN808863_Hu_2008_Bangladesh     GACAAATCTTTTGATGAGGACCTTGAACTAGCTAGCTTCCTGATGGACAGGAGGGTGATA    14759
                                ************** ** ** ****************** * ************** ***

NC_002728_Nipah_Reference       TTACCTAGAGCCGCTCATGAGATACTGGATAATTCATTGACAGGTGCCAGAGAGGAAATT    14813
PP554504_Bat_2023_India         TTACCTAGGGCCGCTCATGAGATACTGGACAATTCATTGACAGGTGCCAGGGAGGAAATT    14756
```

```
JN808863_Hu_2008_Bangladesh      TTACCTAGAGCCGCTCATGAGATACTGGACAATTCATTGACAGGTGCCAGAGAGGAAATT     14819
                                 ******** ******************** ******************** *********

NC_002728_Nipah_Reference        GCTGGTTTATTAGATACAACTAAAGGCTTGATCAGATCAGGGCTAAGAAAGAGTGGACTT     14873
PP554504_Bat_2023_India          GCTGGTCTATTAGATACAACTAAAGGCTTGATAAGATCAGGGTTGAGAAAGAGTGGAATT     14816
JN808863_Hu_2008_Bangladesh      GCTGGTCTATTAGATACAACTAAAGGCTTGATAAGATCAGGGTTGAGAAAGAGTGGAATT     14879
                                 ****** ************************* ********* * ************ **

NC_002728_Nipah_Reference        CAGCCAAAGTTAGTTTCTAGATTATCTCATCATGATTATAATCAATTTTTAATACTGAAC     14933
PP554504_Bat_2023_India          CAGCCAAGGTTAGTTTCCAGATTGTCTCATCATGATTACAATCAATTTTTAATACTAAAT     14876
JN808863_Hu_2008_Bangladesh      CAGCCAAGGTTAGTTTCCAGATTGTCTCATCATGATTACAATCAATTTTTAATACTAAAT     14939
                                 ******* ********* ***** ************** ***************** ** 

NC_002728_Nipah_Reference        AAACTTCTATCAAACAGAAGACAAAATGACTTGATATCATCAAATACTTGCTCAGTTGAC     14993
PP554504_Bat_2023_India          AAACTTCTATCAAACAGAAAACAGAATGATTTGATATCATCAAATACTTGCTCAGTTGAC     14936
JN808863_Hu_2008_Bangladesh      AAACTTCTATCAAACAGAAAACAGAATGATTTGATATCATCAAATACTTGCTCAGTTGAC     14999
                                 ******************* *** ***** ******************************

NC_002728_Nipah_Reference        TTGGCACGAGCATTGAGATCTCACATGTGGAGGGAATTAGCGTTAGGTAGAGTAATATAC     15053
PP554504_Bat_2023_India          TTAGCACGAGCATTGAGATCCCACATGTGGAGGGAATTAGCTTTAGGAAGAGTAATATAC     14996
JN808863_Hu_2008_Bangladesh      TTAGCACGAGCATTGAGATCCCACATGTGGAGGGAATTAGCTTTAGGAAGAGTAATATAC     15059
                                 ** ***************** ******************** ***** ************

NC_002728_Nipah_Reference        GGTCTTGAGGTACCAGATGCACTTGAGGCTATGGTGGGAAGGTATATAACAGGGAGCTTA     15113
PP554504_Bat_2023_India          GGTCTTGAGGTACCAGATGCCCTTGAAGCTATGGTGGGGAGGTATATAACAGGAAGCTTA     15056
JN808863_Hu_2008_Bangladesh      GGTCTTGAGGTACCAGATGCCCTTGAAGCTATGGTGGGGAGGTATATAACAGGAAGCTTA     15119
                                 ******************** ***** *********** ************** ******

NC_002728_Nipah_Reference        GAGTGCCAAATTTGTGAGCAGGGAAACACGATGTATGGGTGGTTCTTTGTACCTAGGGAT     15173
PP554504_Bat_2023_India          GAGTGCCAAATCTGTGAGCAGGGAAACACGATGTATGGGTGGTTTTTTGTACCTAGGGAT     15116
JN808863_Hu_2008_Bangladesh      GAGTGCCAAATCTGTGAGCAGGGAAACACGATGTATGGGTGGTTTTTTGTACCTAGGGAT     15179
                                 *********** ******************************** ***************

NC_002728_Nipah_Reference        TCCCAATTGGATCAGGTAGATAGAGAGCACTCATCAATAAGAGTACCTTATGTAGGATCA     15233
PP554504_Bat_2023_India          TCCCAATTAGATCAGGTAGATAAAGAGCACTCATCAATAAGAGTACCTTATGTAGGATCA     15176
JN808863_Hu_2008_Bangladesh      TCCCAATTAGATCAGGTAGATAAAGAGCACTCATCAATAAGAGTACCTTATGTAGGATCA     15239
                                 ******** ************* *************************************

NC_002728_Nipah_Reference        AGTACGGATGAAAGATCGGATATCAAACTAGGGAATGTCAAAAGACCAACTAAGGCCTTG     15293
PP554504_Bat_2023_India          AGTACAGATGAGAGATCAGATATCAAGCTGGGGAATGTCAAGAGACCGACTAAAGCCTTA     15236
JN808863_Hu_2008_Bangladesh      AGTACAGATGAGAGATCAGATATCAAGCTGGGGAATGTCAAGAGACCGACTAAAGCCTTA     15299
                                 ***** ***** ***** ******** ** *********** ***** ***** ***** 

NC_002728_Nipah_Reference        CGTTCTGCTATCAGAATTGCGACAGTATATACTTGGGCCTATGGGGACAATGAAGAGTGT     15353
PP554504_Bat_2023_India          CGTTCTGCTATCAGGATTGCGACAGTATATACTTGGGCCTATGGAGATAATGAAGAGTGT     15296
JN808863_Hu_2008_Bangladesh      CGTTCTGCTATCAGGATTGCGACAGTATATACTTGGGCCTATGGAGATAATGAAGAGTGT     15359
                                 ************** ***************************** ** ************

NC_002728_Nipah_Reference        TGGTATGAAGCTTGGTACCTAGCGTCTCAGAGGGTAAACATAGACTTAGATGTATTGAAA     15413
PP554504_Bat_2023_India          TGGTATGAAGCTTGGTACCTAGCGTCTCAGAGGGTAAACATAGATTTAGATGTATTGAAG     15356
JN808863_Hu_2008_Bangladesh      TGGTATGAAGCTTGGTACCTAGCGTCTCAGAGGGTAAACATAGATTTAGATGTATTGAAG     15419
                                 ******************************************** ************** 

NC_002728_Nipah_Reference        GCTATAACCCCAGTTTCCACTTCAAACAATTTATCCCATAGATTGAGAGATAAATCCACA     15473
PP554504_Bat_2023_India          GCTATAACCCCAGTCTCTACTTCAAACAATTTATCCCATAGATTGAGAGACAAATCCACA     15416
JN808863_Hu_2008_Bangladesh      GCTATAACCCCAGTCTCTACTTCAAACAATTTATCCCATAGATTGAGAGACAAATCCACA     15479
                                 ************** ** ******************************** *********

NC_002728_Nipah_Reference        CAATTTAAGTTTGCAGGGAGTGTACTCAACAGAGTTTCTAGATATGTTAACATAAGCAAT     15533
PP554504_Bat_2023_India          CAATTCAAGTTCGCAGGGAGTGTACTCAACAGGGTTTCTAGATATGTCAACATAAGCAAT     15476
JN808863_Hu_2008_Bangladesh      CAATTCAAGTTCGCAGGGAGTGTACTCAACAGGGTTTCTAGATATGTCAACATAAGCAAT     15539
                                 ***** ***** ********************* ************* ************

NC_002728_Nipah_Reference        GACAATCTAGATTTCAGAATTGAGGGAGAAAAGGTAGATACGAATCTTATTTATCAACAA     15593
PP554504_Bat_2023_India          GATAATCTGGATTTCAGAATTGAGGGGGAGAAGGTAGATACGAATCTNNNNNNNNNNNNN     15536
JN808863_Hu_2008_Bangladesh      GATAATCTGGATTTCAGAATTGAGGGGGAGAAGGTAGATACGAATCTTATTTATCAACAG     15599
                                 ** ***** ***************** ** *****************

NC_002728_Nipah_Reference        GCAATGCTATTAGGGTTATCGGTATTGGAAGGTAAATTCAGATTGAGATTAGAAACTGAT     15653
PP554504_Bat_2023_India          NNNNNNNNNNNNNNNNNNNNNGTATTGGAAGGTAAATTCAGATTGAGATTGGAGACAGAT     15596
JN808863_Hu_2008_Bangladesh      GCAATGCTGTTAGGATTATCAGTATTGGAAGGTAAATTCAGATTGAGATTGGAGACAGAT     15659
                                                      ***************************** ** ** ***

NC_002728_Nipah_Reference        GATTACAACGGGATATATCACTTACACGTAAAGGATAATTGTTGTGTCAAAGAAGTGGCT     15713
PP554504_Bat_2023_India          GATTACAATGGGATATATCACTTACATGTTAAGGATAATTGTTGTGTCAAAGAAGTGGCT     15656
JN808863_Hu_2008_Bangladesh      GATTACAATGGAATATATCACTTACATGTTAAGGATAATTGTTGTGTCAAAGAAGTGGCT     15719
                                 ******** ** ************** ** ******************************

NC_002728_Nipah_Reference        GATGTAGGCCAAGTAGACGCTGAGTTGCCTATCCCAGAATATACTGAAGTGGATAACAAT     15773
PP554504_Bat_2023_India          GATGTAGGCCAAGTGGACGCTGAATTGCCTATCCCAGAGTATACTGAGGTTGATAACAAT     15716
JN808863_Hu_2008_Bangladesh      GATGTAGGCCAAGTGGACGCTGAATTGCCTATCCCAGAGTATACTGAGGTTGATAACAAT     15779
                                 ************** ******** ************** ******** ** *********

NC_002728_Nipah_Reference        CATCTTATATATGATCCAGACCCCGTTTCAGAAATAGATTGCAGCCGTCTTTCTAATCAG     15833
PP554504_Bat_2023_India          CATCTTATATATGATCCAGATCCTGTTTCAGAAATAGATTGCAGCCGTCTTTCTAATCAG     15776
JN808863_Hu_2008_Bangladesh      CATCTTATATATGATCCAGATCCTGTTTCAGAAATAGATTGCAGCCGTCTTTCTAATCAG     15839
                                 ******************** ** ************************************
```

```
NC_002728_Nipah_Reference       GAGTCCAAATCAAGAGAATTAGACTTTCCTTTATGGTCAACTGAGGAACTTCATGATGTC     15893
PP554504_Bat_2023_India         GAGTCAAAATCAAGAGAATTAGACTTCCCTTTATGGTCAACTGAGGAGCTTCATGATGCC     15836
JN808863_Hu_2008_Bangladesh     GAGTCAAAATCAAGAGAATTAGACTTCCCTTTATGGTCAACTGAGGAGCTTCATGATGCC     15899
                                ***** ******************** ******************** ********** *

NC_002728_Nipah_Reference       CTAGCTAAGACTGTTGCTCAGACCGTTCTTGAGATTATAACAAAGGCTGACAAGGATGTT     15953
PP554504_Bat_2023_India         CTTGCTAAGACCGTTGCTCAGACCGTTCTTGAGATTATAACAAAGGCTGACAAGGATGTT     15896
JN808863_Hu_2008_Bangladesh     CTTGCTAAGACCGTTGCTCAGACCGTTCTTGAGATTATAACAAAGGCTGACAAGGATGTT     15959
                                ** ******** ************************************************

NC_002728_Nipah_Reference       TTAAAGCAACACCTTGCAATAGACTCTGACGATAACATCAACAGCTTAATCACAGAATTT     16013
PP554504_Bat_2023_India         TTAAAGCAACACCTTGCAATAGACTCTGACGATAACATCAACAGTTTAATCACAGAATTT     15956
JN808863_Hu_2008_Bangladesh     TTAAAGCAACACCTTGCAATAGACTCTGACGATAACATCAACAGTTTAATCACAGAATTT     16019
                                ******************************************** ***************

NC_002728_Nipah_Reference       CTAATAGTTGATCCTGAACTGTTTGCACTTTATCTAGGACAATCTATATCAATAAAATGG     16073
PP554504_Bat_2023_India         CTAATAGTTGATCCTGAACTGTTTGCACTTTATCTTGGACAATCTATAGCAATAAAATGG     16016
JN808863_Hu_2008_Bangladesh     CTAATAGTTGATCCTGAACTGTTTGCACTTTATCTTGGACAATCTATAGCAATAAAATGG     16079
                                *********************************** ************* **********

NC_002728_Nipah_Reference       GCCTTTGAAATTCATCATAGGCGTCCTAGAGGAAGACATACTATGGTCGACCTATTGTCA     16133
PP554504_Bat_2023_India         GCTTTCGAAATTCATCATAGGCGTCCTAGAGGAAGACATACTATGGTTGATCTGTTGTCA     16076
JN808863_Hu_2008_Bangladesh     GCTTTCGAAATTCATCATAGGCGTCCTAGAGGAAGACATACTATGGTTGATCTGTTGTCA     16139
                                ** ** ***************************************** ** ** ******

NC_002728_Nipah_Reference       GATCTTGTATCAAATACATCAAAGCACACTTACAAAGTGTTGTCAAATGCCTTGTCACAT     16193
PP554504_Bat_2023_India         GATCTTATATCAAACACATCAAAGCACACTTACAAAGTGCTGTCAAACGCCTTGTCACAT     16136
JN808863_Hu_2008_Bangladesh     GATCTTATATCAAACACATCAAAGCACACTTACAAAGTGCTGTCAAACGCCTTGTCACAT     16199
                                ****** ******* ************************ ******* ************

NC_002728_Nipah_Reference       CCTAGAGTATTCAAGAGATTTGTAAACTGTGGCTTGCTATTGCCTACACAGGGTCCTTAC     16253
PP554504_Bat_2023_India         CCTAGAGTATTCAAGAGATTTGTAAACTGTGGCTTACTGTTGCCTACACAGGGTCCTTAC     16196
JN808863_Hu_2008_Bangladesh     CCTAGAGTATTCAAGAGATTTGTAAACTGTGGCTTACTGTTGCCTACACAGGGTCCTTAC     16259
                                *********************************** ** *********************

NC_002728_Nipah_Reference       CTTCATCAACAAGATTTTGAAAAGTTGTCTCAAAACCTTCTTGTAACATCTTATATGATT     16313
PP554504_Bat_2023_India         CTTCATCAACAAGATTTTGAAAAGCTGTCTCAAAATCTCCTTGTAACATCTTACATGATT     16256
JN808863_Hu_2008_Bangladesh     CTTCATCAACAAGATTTTGAAAAGCTGTCTCAAAATCTCCTTGTAACATCTTACATGATT     16319
                                ************************ ********** ** ************** ******

NC_002728_Nipah_Reference       TATCTAATGAACTGGTGTGACTTCAAGAAATCCCCCTTTTTAATCGCCGAACAGGATGAA     16373
PP554504_Bat_2023_India         TATCTAATGAACTGGTGTGACTTCAAGAAATACCCCTTTTTAATCGCCGAACAGGATGAA     16316
JN808863_Hu_2008_Bangladesh     TATCTAATGAACTGGTGTGACTTCAAGAAATACCCCTTTTTAATCGCCGAACAGGATGAA     16379
                                ******************************* ****************************

NC_002728_Nipah_Reference       ACTGTGATAAGTCTACGAGAGGATATAATAACATCCAAACATCTCTGTGTTATAATTGAC     16433
PP554504_Bat_2023_India         ACTGTGATTAACCTACGAGAGGATATAATAACATCCAAACATCTCTGCGTTATAATTGAC     16376
JN808863_Hu_2008_Bangladesh     ACTGTGATTAACCTACGAGAGGATATAATAACATCCAAACATCTCTGCGTTATAATTGAT     16439
                                ******** *  *********************************** ***********

NC_002728_Nipah_Reference       TTATATGCAAATCACCATAAACCTCCTTGGATAATAGATCTAAACCCACAAGAAAAAATA     16493
PP554504_Bat_2023_India         TTATACGCAAATCACCATAAACCTCCTTGGATAATAGATCTGAACCCACAAGAAAAAATA     16436
JN808863_Hu_2008_Bangladesh     TTATACGCAAATCACCATAAACCTCCTTGGATAATAGATCTGAACCCACAAGAAAAAATA     16499
                                ***** *********************************** ******************

NC_002728_Nipah_Reference       TGTGTACTGCGTGACTTTATTTCTAAATCTAGGCATGTGGACACGTCCTCCAGATCATGG     16553
PP554504_Bat_2023_India         TGTGTACTTCGTGATTTTATTTCCAAATCTAGGCATATGGATACGTCCTCTCGATCATGG     16496
JN808863_Hu_2008_Bangladesh     TGTGTACTTCGTGATTTTATTTCCAAATCTAGGCATATGGATACGTCCTCTAGATCATGG     16559
                                ******** ***** ******** ************ **** ********  ********

NC_002728_Nipah_Reference       AATACTTCTGACCTGGATTTTGTAATATTCTATGCATCTTTGACTTATTTGAGAAGAGGT     16613
PP554504_Bat_2023_India         AATACTTCTGACCTGGATTTTGTGATATTCTATGCATCTTTAACTTATTTGAGAAGAGGC     16556
JN808863_Hu_2008_Bangladesh     AATACTTCTGACCTGGATTTTGTGATATTCTATGCATCTTTAACTTATTTGAGAAGAGGC     16619
                                *********************** ***************** *****************

NC_002728_Nipah_Reference       ATAATAAAACAATTAAGGATAAGACAAGTTACTGAGGTTATAGATACCACAACAATGTTA     16673
PP554504_Bat_2023_India         ATAATAAAGCAATTAAGGATAAGACAAGTTACCGAAGTTATAGATACCACAACAATGTTA     16616
JN808863_Hu_2008_Bangladesh     ATAATAAAGCAATTAAGGATAAGACAAGTTACCGAAGTTATAGATACCACAACAATGTTA     16679
                                ******** *********************** ** ************************

NC_002728_Nipah_Reference       AGGGACAATATAATTGTAGAGAATCCTCCTATTAAAACAGGAGTGTTAGACATCAGAGGT     16733
PP554504_Bat_2023_India         AGGGACAATATAATTGTGGAGAATCCTCCTATCAAAACAGGGGTGTTAGATATCAGAGGT     16676
JN808863_Hu_2008_Bangladesh     AGGGACAATATAATTGTGGAGAATCCTCCTATCAAAACAGGGGTGTTAGATATCAGAGGT     16739
                                ***************** ************** ******** ******** *********

NC_002728_Nipah_Reference       TGTATAATATACAATTTAGAGGAAATCCTGTCTATGAACACAAAATCAGCATCAAAAAAG     16793
PP554504_Bat_2023_India         TGTATAATATACAATTTAGAGGAAATCCTGTCTATGAACACGAAATCATCGTCCAAAAAG     16736
JN808863_Hu_2008_Bangladesh     TGTATAATATACAATTTAGAGGAAATCCTGTCTATGAATACGAAATCATCGTCCAAAAAG     16799
                                ************************************** ** ****** * ** ******

NC_002728_Nipah_Reference       ATCTTTAATCTTAATAGTAGGCCGTCAGTGGAGAATCATAAATATAGAAGGATAGGTCTC     16853
PP554504_Bat_2023_India         ATCTTTAACCTTAATAGNNNNNNNNNNGTGGAGAATCACAAATATAGAAGGATAGGCCTC     16796
JN808863_Hu_2008_Bangladesh     ATCTTTAACCTTAATAGTAAGCCGTCGGTGGAGAATCACAAATATAGAAGGATAGGCCTC     16859
                                ******** ********          ************ **************** ***

NC_002728_Nipah_Reference       AACTCATCATCTTGTTACAAGGCATTAAATCTATCACCTCTGATTCAAAGGTATTTGCCG     16913
PP554504_Bat_2023_India         AACTCATCATCATGTTACAAGGCATTGAATTTATCACCTCTAATTCAGAGGTATTTGCCG     16856
JN808863_Hu_2008_Bangladesh     AACTCATCATCATGTTACAAGGCATTGAATTTATCACCTCTAATTCAGAGGTATTTGCCG     16919
```

```
                                  *********** ************** *** ********** ***** ************

NC_002728_Nipah_Reference         TCGGGAGCTCAAAGGTTGTTTATAGGAGAAGGTTCTGGGAGCATGATGTTATTATATCAG      16973
PP554504_Bat_2023_India           TCAGGAGCTCAAAGATTGTTTATAGGAGAAGGTTCTGGAAGCATGATGTTGTTATATCAG      16916
JN808863_Hu_2008_Bangladesh       TCAGGAGCTCAAAGATTGTTTATAGGAGAAGGTTCTGGAAGCATGATGTTGTTATATCAG      16979
                                  ** *********** *********************** *********** *********

NC_002728_Nipah_Reference         TCTACATTGGGGCAATCAATTTCTTTTTACAATTCAGGTATAGATGGAGATTATATACCA      17033
PP554504_Bat_2023_India           TCTACATTGGGGCAATCAATTTCTTTTTACAATTCAGGCATAGACGGAGATTATATACCA      16976
JN808863_Hu_2008_Bangladesh       TCTACATTGGGGCAATCAATTTCTTTTTACAATTCAGGCATAGACGGAGATTATATACCA      17039
                                  ************************************** ***** ***************

NC_002728_Nipah_Reference         GGTCAAAGAGAACTGAAACTATTTCCCTCTGAATACTCAATTGCTGAGGAAGACCCATCT      17093
PP554504_Bat_2023_India           GGTCAGAGAGAACTGAAGCTATTTCCATCCGAATACTCGATTGCTGAGGAAGACCCATCT      17036
JN808863_Hu_2008_Bangladesh       GGTCAGAGAGAACTGAAGCTATTTCCATCCGAATACTCGATTGCTGAGGAAGACCCATCT      17099
                                  ***** *********** ******** ** ******** *********************

NC_002728_Nipah_Reference         CTGACGGGGAAATTGAAAGGACTAGTGGTGCCCCTATTCAATGGAAGACCAGAAACAACA      17153
PP554504_Bat_2023_India           TTGGCGGGAAAGTTGAAAGGACTGGTGGTGCCTTTATTTAATGGGAGACCAGAAACAACA      17096
JN808863_Hu_2008_Bangladesh       TTGGCGGGAAAGTTGAAGGGACTGGTGGTGCCTTTATTTAATGGGAGACCAGAAACAACA      17159
                                   ** **** ** ***** ***** ********  **** ***** ***************

NC_002728_Nipah_Reference         TGGATCGGGAATTTAGACTCCTACGAGTATATCATAAATAGGACAGCGGGGCGAAGTATA      17213
PP554504_Bat_2023_India           TGGATTGGGAATTTAGACTCTTACGAGTATATCATAAATAGGACAGCGGGGCGAAGTATA      17156
JN808863_Hu_2008_Bangladesh       TGGATTGGGAATTTAGACTCTTACGAGTATATCATAAATAGGACAGCGGGGCGAAGTATA      17219
                                  ***** ************** ***************************************

NC_002728_Nipah_Reference         GGTCTTGTCCATTCTGACATGGAGTCTGGGATTGACAAAAATGTAGAGGAGATACTAGTA      17273
PP554504_Bat_2023_India           GGTCTTGTCCATTCTGACATGGAGTCTGGGATTGACAAAAATGTAGAAGAGATACTAGTA      17216
JN808863_Hu_2008_Bangladesh       GGTCTTGTCCATTCTGACATGGAGTCTGGGATTGACAAAAATGTAGAAGAGATACTAGTA      17279
                                  *********************************************** ************

NC_002728_Nipah_Reference         GAACATTCCCATCTAATATCTATCGCGATAAATGTTATGATGGAGGACGGACTATTAGTA      17333
PP554504_Bat_2023_India           GAACATTCTCATCTAATATCTATCGCGATAAATGTTATGATGGAGGATGGATTATTAGTA      17276
JN808863_Hu_2008_Bangladesh       GAACATTCTCATCTAATATCTATCGCGATAAATGTTATGATGGAGGATGGATTATTAGTA      17339
                                  ******** ************************************** *** ********

NC_002728_Nipah_Reference         TCCAAGATAGCATACACCCCTGGATTCCCAATCTCAAGATTATTTAACATGTACAGATCA      17393
PP554504_Bat_2023_India           TCTAAGATAGCATACACCCCCGGATTCCCAATCTCAAGATTATTTAACATGTACAGGTCA      17336
JN808863_Hu_2008_Bangladesh       TCTAAGATAGCATACACCCCCGGATTCCCAATCTCAAGATTATTTAACATGTACAGGTCA      17399
                                  ** ***************** *********************************** ***

NC_002728_Nipah_Reference         TATTTCGGACTAGTACTGGTGTGTTTCCCAGTATATAGTAATCCAGATTCTACTGAAGTA      17453
PP554504_Bat_2023_India           TATTTTGGACTAGTACTGGTGTGTTTCCCAGTGTATAGCAACCCAGATTCTACTGAGGTA      17396
JN808863_Hu_2008_Bangladesh       TATTTTGGACTAGTACTGATGTGTTTCCCAGTGTATAGCAACCCAGATTCTACTGAGGTA      17459
                                  ***** ************ ************* ***** ** ************** ***

NC_002728_Nipah_Reference         TATCTTCTTTGCTTACAGAAGACGGTCAAGACTATTGTTCCCCCGCAAAAAGTCCTTGAG      17513
PP554504_Bat_2023_India           TATCTTCTTTGCTTACAAAAGACAGTCAAGACTATTATCCCTCCACAAAGAGTCCTTGAG      17456
JN808863_Hu_2008_Bangladesh       TATCTTCTTTGCTTACAAAAGACAGTCAAGACTATTATCCCTCCACAAAGAGTCCTTGAG      17519
                                  ***************** ***** ************ * ** ** **** **********

NC_002728_Nipah_Reference         CACTCTAATTTGCACGATGAAGTCAATGACCAGGGAATAACATCAGTGATTTTTAAAATC      17573
PP554504_Bat_2023_India           CATTCTGATTTGCACGATGAGGTCAATGATCAGGGGATAACATCAGTAATTTTTAAGATC      17516
JN808863_Hu_2008_Bangladesh       CACTCTGATTTGCACGATGAGGTCAATGATCAGGGGATAACATCAGTAATTTTTAAGATC      17579
                                  ** *** ************* ******** ***** *********** ******** ***

NC_002728_Nipah_Reference         AAGAATTCACAGTCTAAGCAGTTCCACGATGATCTAAAGAAGTACTATCAGATTGACCAA      17633
PP554504_Bat_2023_India           AAGAATTCTCAATCTAAACAGTTCCATGAAGATCTAAAGAAATACTATCATATTGACCAA      17576
JN808863_Hu_2008_Bangladesh       AAGAATTCTCAATCTAAACAGTTCCATGAAGATCTAAAGAAATACTATCATATTGACCAA      17639
                                  ******** ** ***** ******** ** *********** ******** *********

NC_002728_Nipah_Reference         CCTTTTTTTGTACCAACTAAAATCACTAGTGATGAACAAGTACTTCTCCAAGCAGGGCTG      17693
PP554504_Bat_2023_India           CCTTTCTTTGTACCAACTAAAATCACTAGTGATGAACGAGTACTTCTCCAGGCAGGGCTA      17636
JN808863_Hu_2008_Bangladesh       CCTTTCTTTGTACCAACTAAAATCACTAGTGATGAACAAGTACTTCTCCAAGCAGGGCTA      17699
                                  ***** ******************************* ************ ********

NC_002728_Nipah_Reference         AAACTCAATGGGCCAGAAATTCTTAAGAGTGAAATCAGTTATGATATCGGTTCAGATATC      17753
PP554504_Bat_2023_India           AAACTCAATGGGCCAGAAATCCTTAAGAGTGAAATCAGTTATGATATCGGTTCAGATATC      17696
JN808863_Hu_2008_Bangladesh       AAACTCAATGGGCCAGAAATCCTTAAGAGTGAAATCAGTTATGATATCGGTTCAGATATC      17759
                                  ******************** ***************************************

NC_002728_Nipah_Reference         AATACATTAAGAGACACCATCATAATTATGTTAAATGAGGCTATGAATTATTTTGATGAC      17813
PP554504_Bat_2023_India           AACACATTAAGAGACACCATCATAATTATGTTAAATGAGGCCATGAATTATTTTGATGAT      17756
JN808863_Hu_2008_Bangladesh       AACACATTAAGAGACACCATCATAATTATGTTAAATGAGGCCATGAATTATTTTGATGAT      17819
                                  ** ************************************** *****************

NC_002728_Nipah_Reference         AACAGATCACCTTCACACCACCTAGAACCCTATCCAGTTTTGGAGAGAACTAGAATTAAA      17873
PP554504_Bat_2023_India           AACAGGTCGCCTTCACATCATTTGGAACCTTATCCAGTTTTGGAGAGAACTAGAATTAAA      17816
JN808863_Hu_2008_Bangladesh       AATAGGTCGCCTTCACATCATTTGGAACCTTATCCAGTTTTGGAGAGAACTAGAATTAAA      17879
                                  ** ** ** ******** **  * ***** ******************************

NC_002728_Nipah_Reference         ACAATAATGAATTGTGTGACTAAAAAAGTGATTGTCTACTCACTTATCAAGTTCAAGGAC      17933
PP554504_Bat_2023_India           ACAATAATGAATCGTGTAACTAAGAAAGTGATTGTCTACTCACTCATCAAGTTCAAAGAT      17876
JN808863_Hu_2008_Bangladesh       ACAATAATGAATCGTGTAACTAAGAAAGTGATTGTCTACTCACTCATCAAGTTCAAAGAT      17939
                                  ************ **** ***** ******************** *********** **

NC_002728_Nipah_Reference         ACCAAAAGCTCAGAACTTTATCACATCAAAAATAACATCAGAAGAAAGTTCTAATCTTA      17993
```

```
PP554504_Bat_2023_India         ACCAAAAGTTCAGAGCTCTACCACATTAAAAATAATATCAGAAGGAAAGTTCTAATCTTA     17936
JN808863_Hu_2008_Bangladesh     ACCAAAAGTTCAGAGCTCTACCACATTAAAAATAATATCAGAAGGAAAGTTCTAATCTTA     17999
                                ******** ***** ** ** ***** ******** ******** ***************

NC_002728_Nipah_Reference       GATTTCAGATCGAAGCTCATGACAAAGACTCTACCTAAAGGGATGCAAGAGAGAAGAGAA     18053
PP554504_Bat_2023_India         GATTTCAGATCAAAGCTCATGACAAAGACTCTACCTAAAGGGATGCAAGAGAGAAGAGAA     17996
JN808863_Hu_2008_Bangladesh     GATTTCAGATCAAAGCTCATGACAAAGACTCTACCTAAAGGGATGCAAGAGAGAAGAGAA     18059
                                *********** ************************************************

NC_002728_Nipah_Reference       AAAAACGGTTTCAAAGAAGTTTGGATAGTAGATTTATCGAATCGAGAAGTTAAAATCTGG     18113
PP554504_Bat_2023_India         AAAAGCGGTTTCAAGGAGGTTTGGATAGTAGACTTATCGAACCGAGAAGTCAAAATTTGG     18056
JN808863_Hu_2008_Bangladesh     AAAAGCGGTTTCAAGGAGGTTTGGATAGTAGACTTATCGAACCGAGAAGTCAAAATCTGG     18119
                                **** ********* ** ************** ******** ******** ***** ***

NC_002728_Nipah_Reference       TGGAAGATAATCGGATACATATCTATTATCTGATTTAACCTTCCAAATCCAAGACCAACT     18173
PP554504_Bat_2023_India         TGGAAGATAATCGGATACATATCCATTATCTGATTTGATCTTCCAAGTCCAGAACCGACC     18116
JN808863_Hu_2008_Bangladesh     TGGAAGATAATCGGATACATATCCATTATCTGATTTGATCTTCCAAGTCCAGAACCGACC     18179
                                *********************** ************ * ******* ****  *** **

NC_002728_Nipah_Reference       GATAACTTATGTTGATCTAAGGTTCAGTTATTAAGAAAAACTTAATAACGATTCTTCTTT     18233
PP554504_Bat_2023_India         GACAATTTGTGCTGGTCCAAAGTTCAGTTATTAAGAAAAACTTAATAAC-----------     18165
JN808863_Hu_2008_Bangladesh     GACAATTTGTGCTGGTCCAAAATTCAGTTATTAAGAAAAACTTAATAACGATTCTTCTTT     18239
                                ** ** ** ** ** ** **  ***************************

NC_002728_Nipah_Reference       ACCCTTGTTCGGT        18246
PP554504_Bat_2023_India         -------------        18165
JN808863_Hu_2008_Bangladesh     ACCCTTGTTCGGT        18252
```

Supplementary figure 1: Multiple sequence alignment of the Nipah virus reference sequence (NC_002728) with the sequence obtained in the present study from bats (PP554504), and a sequence from Bangladesh (JN808863), done using Clustal Omega. The nucleotide changes in the sequences are highlighted in green and the * symbol denotes conserved region.

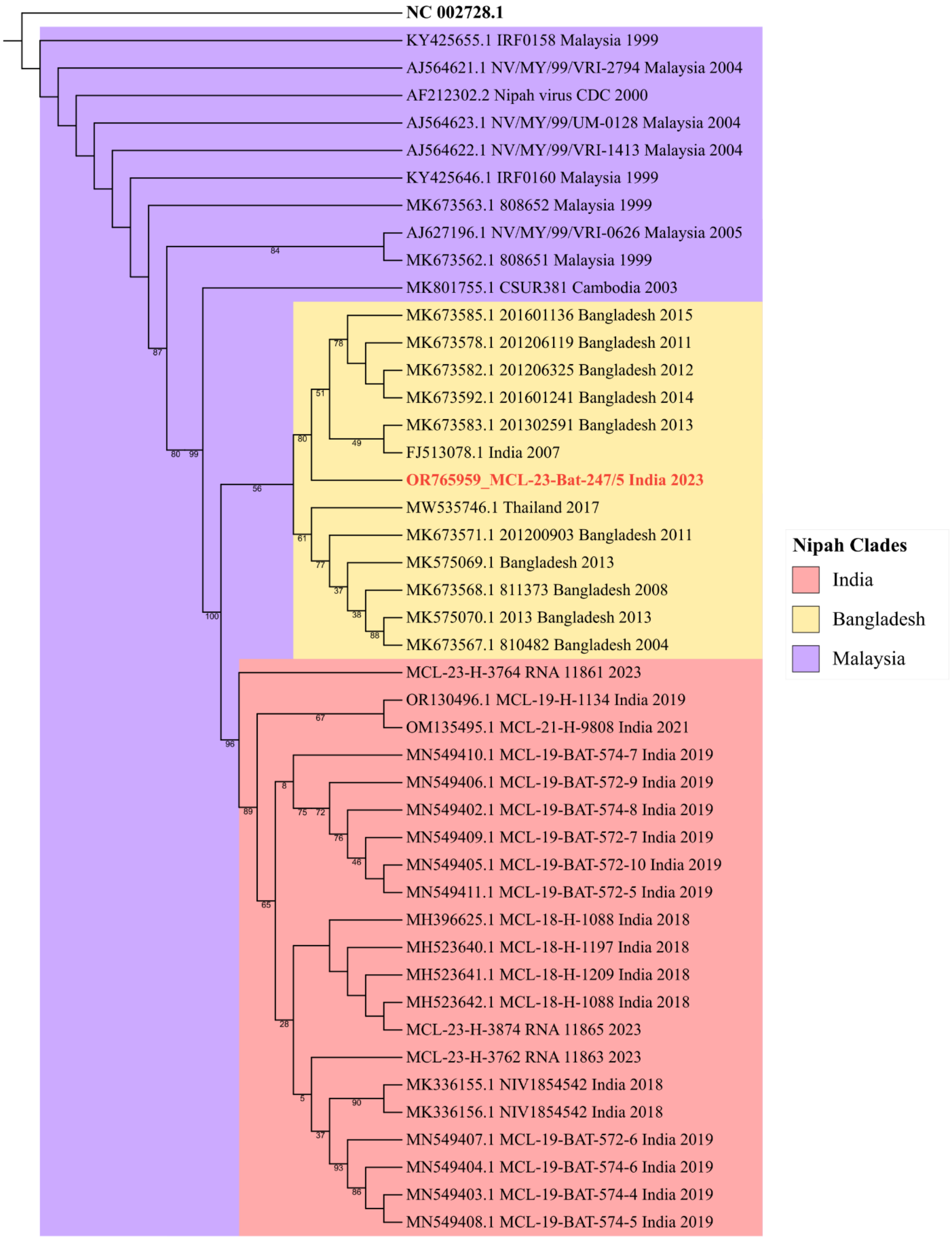

Supplementary Figure 2: The maximum likelihood phylogenetic tree of the partial N gene sequence (342 bp) of Nipah virus retrieved from the *P. medius* along with the NiV reference sequence as well as sequences from the NiV-Bangladesh (background coloured in light yellow), India (background coloured in light red) and Malaysia (background coloured in lavender) clades. The sequence (highlighted in red) retrieved in the present study clustered with the NiV-Bangladesh genotype sequences.

Supplementary table 1: Details of missing nucleotide positions in Nipah virus genome retrieved in the study

Gene	Nucleotide position	Gaps (no. of nucleotides)
N	93-101	9
P	3171-3223	53
P	4237-4253	17
M	6088-6163	76
F	6391-6490	99
F	7448-7472	25
F	8260-8641	382
G	8737-8846	110
G	10017-10042	26
G	10726-11112	387
L	11534-11631	98
L	11808-11830	23
L	12083-12120	38
L	15524-15557	34
L	16754-16763	10

Supplementary table 2: Amino acid substitutions in the Nipah virus genome retrieved in comparison with other NiV sequences

Accession ID	Host	Country	C											N												
			25	26	33	34	37	39	40	62	67	83	98	137	139	152	188	211	345	387	429	432	457	464	495	502
NC002728			**V**	**F**	**I**	**K**	**P**	**K**	**I**	**M**	**T**	**R**	**Y**	**D**	**S**	**G**	**E**	**Q**	**M**	**D**	**I**	**G**	**N**	**V**	**N**	**I**
MK673558	Swine	Malaysian	.	.	.	.	.	.	.	.	.	.	.	.	.	.	.	.	.	.	.	.	.	.	.	.
AF212302	Human	Malaysian	.	.	.	.	.	.	.	.	.	.	.	.	.	.	.	.	.	.	.	.	.	.	.	.
MK673562	Human	Malaysian	.	.	.	.	.	.	.	.	.	.	.	.	.	.	.	.	.	.	.	.	.	.	S	.
AY029767	Human	Malaysian	.	.	.	.	.	.	.	.	.	.	.	.	.	.	.	.	.	.	.	.	.	.	.	.
MK673561	Swine	Malaysian	.	.	.	.	.	.	.	.	.	.	.	.	.	.	.	.	.	.	.	.	.	.	.	.
MK673560	Swine	Malaysian	.	.	.	.	.	.	.	.	.	.	.	.	.	.	.	.	.	.	.	.	.	.	.	.
AJ627196	Swine	Malaysian	.	.	.	.	.	.	.	.	.	.	.	.	R	.	.	.	I	.	.	.	.	.	.	.
MK801755	Bat	Cambodia	A	.	T	E	.	.	.	.	.	.	.	.	.	.	.	.	.	.	V	E	D	.	.	T
AY988601	Human	Bangladesh	A	.	T	E	H	R	.	V	A	K	.	.	.	.	D	.	.	N	V	E	D	.	.	.
MK673564	Human	Bangladesh	A	.	T	E	H	R	.	V	A	K	.	.	.	.	D	.	.	N	V	E	D	.	.	.
MK673574	Human	Bangladesh	A	.	T	E	H	R	.	V	A	K	.	.	.	.	.	.	.	N	V	E	D	.	.	.
MK673565	Human	Bangladesh	A	.	T	E	H	R	.	V	A	K	.	.	.	.	.	.	.	N	V	E	D	.	.	.
MK673572	Human	Bangladesh	A	.	T	E	H	R	.	V	A	K	.	.	.	.	.	.	.	N	V	E	D	.	.	.
MK673586	Human	Bangladesh	A	.	T	E	H	R	.	V	A	K	.	.	.	.	.	.	.	N	V	E	D	.	.	.
MK673579	Human	Bangladesh	A	.	T	E	H	R	.	V	A	K	.	.	.	.	.	.	.	N	V	E	D	.	.	.
MK673580	Human	Bangladesh	A	.	T	E	H	R	.	V	A	K	.	.	.	.	.	.	.	N	V	E	D	.	.	.
MK673581	Human	Bangladesh	A	.	T	E	H	R	.	V	A	K	.	.	.	.	.	.	.	N	V	E	D	M	.	.
MK673590	Human	Bangladesh	A	.	T	E	H	R	.	V	A	K	.	.	.	.	.	.	.	N	V	E	D	.	.	.
FJ513078	Human	India	A	.	T	E	H	R	.	V	A	K	.	.	.	.	.	R	.	N	V	E	D	.	.	.
JN808863	Human	Bangladesh	A	.	T	E	H	R	.	V	A	K	.	.	.	.	.	.	.	N	V	E	D	.	.	.
MK673592	Human	Bangladesh	A	.	T	E	H	R	.	V	A	K	.	.	.	.	.	.	.	N	V	E	D	.	.	.
MK673585	Human	Bangladesh	A	.	T	E	H	R	.	V	A	K	.	.	.	.	.	.	.	N	V	E	D	.	.	.
MK673588	Human	Bangladesh	A	S	T	E	H	R	.	V	A	K	.	.	.	.	.	.	.	N	V	E	D	.	.	.
MK673575	Human	Bangladesh	A	S	T	E	H	R	.	V	A	K	.	.	.	.	.	.	.	N	V	E	D	.	.	.
MK575060	Bat	Bangladesh	A	.	T	E	H	R	.	V	A	K	.	.	.	.	.	.	.	N	V	E	D	.	.	.
MK673587	Human	Bangladesh	A	.	T	E	H	R	.	V	A	K	.	.	.	.	.	.	.	N	V	E	D	.	.	.
**PP554504**	**Bat**	**India**	**A**	**.**	**T**	**E**	**H**	**R**	**.**	**V**	**A**	**K**	**.**	.	.	.	.	.	.	N	V	E	D	.	.	.
MH523640	Human	India	A	.	T	E	H	R	T	V	A	K	.	.	.	.	.	.	.	N	V	E	D	.	.	.
MH523642	Human	India	A	.	T	E	H	R	T	V	A	K	H	.	.	.	.	.	.	N	V	E	D	.	.	.
MN549409	Human	India	A	.	T	E	H	R	T	V	A	K	.	.	.	S	.	.	.	N	V	E	D	.	.	.
MH523641	Human	India	A	.	T	E	H	R	T	V	A	K	.	.	.	.	.	.	.	N	V	E	D	.	.	.
MCL-23-438	Bat	India	A	.	T	E	H	R	T	V	A	K	.	**E**	.	.	.	.	.	N	V	E	D	.	.	.
MH396625	Human	India	A	.	T	E	H	R	T	V	A	K	.	.	.	.	.	.	.	N	V	E	D	.	.	.

Accession ID	Host	Country	N								P													
			503	505	506	508	511	518	520	521	41	52	61	64	69	74	79	81	105	138	139	140	147	156
NC002728			S	R	T	G	E	L	P	A	Q	T	G	S	D	N	S	T	I	D	Y	T	N	M
MK673558	Swine	Malaysia	.	.	.	.	.	.	.	.	.	.	.	.	.	.	.	.	.	.	.	.	.	.
AF212302	Human	Malaysia	.	.	.	.	.	.	.	.	.	.	.	.	.	.	.	.	.	.	.	.	.	.
MK673562	Human	Malaysia	.	.	.	.	.	.	.	.	.	.	.	.	.	.	.	.	.	.	.	.	.	.
AY029767	Human	Malaysia	.	.	.	.	.	.	.	.	.	.	.	.	.	.	.	.	.	.	.	.	.	.
MK673561	Swine	Malaysia	.	.	.	.	.	.	.	.	.	.	.	.	.	.	.	.	.	.	.	.	.	.
MK673560	Swine	Malaysia	.	.	.	.	.	.	.	.	.	.	.	.	.	.	.	.	.	.	.	.	.	.
AJ627196	Swine	Malaysia	.	.	.	.	.	.	.	.	.	.	.	.	.	.	.	.	.	.	.	.	.	.
MK801755	Bat	Cambodia	.	.	.	.	G	P	.	T	R	A	.	.	.	.	P	.	.	.	.	A	.	.
AY988601	Human	Bangladesh	.	K	D	R	.	.	.	T	R	.	.	.	G	S	.	.	.	.	H	S	D	V
MK673564	Human	Bangladesh	.	K	D	R	.	.	.	T	R	.	.	.	G	S	.	.	.	.	H	S	D	V
MK673574	Human	Bangladesh	.	K	D	R	.	.	.	T	R	.	.	.	G	S	.	.	.	.	H	S	D	V
MK673565	Human	Bangladesh	.	K	D	R	.	.	.	T	R	.	.	.	G	S	.	.	.	.	N	S	D	V
MK673572	Human	Bangladesh	.	K	D	R	.	.	.	T	R	.	.	.	G	S	.	.	.	.	H	S	D	V
MK673586	Human	Bangladesh	.	K	D	R	.	.	.	T	R	.	.	P	G	S	.	.	.	.	H	S	D	V
MK673579	Human	Bangladesh	.	K	D	R	.	.	.	T	R	.	.	P	G	S	.	.	.	.	H	S	D	V
MK673580	Human	Bangladesh	.	K	D	R	.	.	.	T	R	.	.	P	G	S	.	.	.	.	H	S	D	V
MK673581	Human	Bangladesh	.	K	D	R	.	.	.	T	R	.	.	P	G	S	.	.	.	.	H	S	D	V
MK673590	Human	Bangladesh	.	K	D	R	.	.	.	T	R	.	R	.	G	S	.	.	.	.	H	S	D	V
FJ513078	Human	India	.	K	D	R	.	.	.	T	R	.	.	.	G	S	.	.	.	.	H	S	D	V
JN808863	Human	Bangladesh	.	K	D	R	.	.	.	T	R	.	.	P	G	S	.	.	.	.	H	S	D	V
MK673592	Human	Bangladesh	.	K	D	R	.	.	.	T	R	.	.	P	G	S	.	.	.	.	H	S	D	V
MK673585	Human	Bangladesh	.	K	D	R	.	.	.	T	R	.	.	P	G	S	.	.	.	.	H	S	D	V
MK673588	Human	Bangladesh	.	K	D	R	.	.	.	T	R	.	.	P	G	S	.	.	.	.	H	S	D	V
MK673575	Human	Bangladesh	.	K	D	R	.	.	.	T	R	.	.	P	G	S	.	.	.	.	H	S	D	V
MK575060	Bat	Bangladesh	.	K	D	R	.	.	.	T	R	.	R	.	G	S	.	A	.	.	H	S	D	V
MK673587	Human	Bangladesh	.	K	D	R	.	.	.	T	R	.	R	.	G	S	.	.	.	.	H	S	D	V
**PP554504**	**Bat**	**India**	**.**	**K**	**D**	**R**	**.**	**.**	**.**	**T**	**R**	**.**	**.**	**P**	**G**	**S**	**.**	**.**	**.**	**.**	**H**	**S**	**D**	**V**
MH523640	Human	India	N	.	D	R	.	.	S	T	R	.	.	.	G	S	.	.	.	.	H	S	D	V
MH523642	Human	India	N	.	D	R	.	.	S	T	R	.	.	.	G	S	.	.	T	.	H	S	D	V
MN549409	Human	India	N	.	D	R	.	.	S	T	R	.	.	.	G	S	.	.	.	.	H	S	D	V
MH523641	Human	India	N	.	D	R	.	.	S	T	R	.	.	.	G	S	.	.	.	.	H	S	D	V
MCL-23- 438	Bat	India	N	.	D	R	.	.	S	T	R	.	.	.	G	S	.	.	.	N	H	S	D	V
MH396625	Human	India	N	.	D	R	.	.	S	T	R	.	.	.	G	S	.	.	.	.	H	S	D	V

Accession ID	Host	Country	P																					
			158	159	179	183	191	195	196	200	203	218	219	223	225	227	228	247	269	274	275	276	277	280
NC002728			**S**	**Y**	**N**	**T**	**V**	**L**	**R**	**D**	**K**	**K**	**E**	**G**	**Q**	**S**	**R**	**Q**	**D**	**S**	**V**	**G**	**G**	**N**
MK673558	Swine	Malaysia	.	.	.	.	.	.	.	.	.	.	.	.	.	.	.	.	.	.	.	.	.	.
AF212302	Human	Malaysia	.	.	.	.	.	.	.	.	.	.	.	.	.	.	.	.	.	.	.	.	.	.
MK673562	Human	Malaysia	.	.	.	.	.	.	.	.	.	.	.	.	.	.	.	.	.	.	.	.	.	.
AY029767	Human	Malaysia	.	.	.	.	.	.	.	.	.	.	.	.	.	.	.	.	.	.	.	.	.	.
MK673561	Swine	Malaysia	.	.	.	.	.	.	.	.	.	.	.	.	.	.	.	.	.	.	.	.	.	.
MK673560	Swine	Malaysia	.	.	.	.	.	.	.	.	.	.	.	.	.	.	.	.	.	.	.	.	.	.
AJ627196	Swine	Malaysia	.	.	.	.	.	.	.	.	.	.	.	.	.	.	.	.	.	R	.	.	.	.
MK801755	Bat	Cambodia	.	H	.	.	.	P	.	.	R	.	.	.	.	.	.	.	.	.	.	.	.	.
AY988601	Human	Bangladesh	.	.	D	A	I	P	K	V	.	R	.	D	.	N	.	.	E	.	A	.	R	I
MK673564	Human	Bangladesh	.	.	D	A	I	P	K	V	.	R	.	D	.	N	.	.	E	.	A	.	R	I
MK673574	Human	Bangladesh	.	.	D	A	I	P	K	V	.	R	.	D	.	N	.	.	E	.	A	.	R	I
MK673565	Human	Bangladesh	.	.	D	A	I	P	K	V	.	R	.	D	.	N	.	.	E	.	A	.	R	I
MK673572	Human	Bangladesh	.	.	D	A	I	P	K	V	.	R	.	D	.	N	.	.	E	.	A	.	R	I
MK673586	Human	Bangladesh	.	.	D	A	I	P	K	V	.	R	.	D	.	N	K	.	E	.	A	.	R	I
MK673579	Human	Bangladesh	.	.	D	A	I	P	K	V	.	R	.	D	.	N	.	.	E	.	A	.	R	I
MK673580	Human	Bangladesh	.	.	D	A	I	P	K	V	.	R	.	D	.	N	.	.	E	.	A	.	R	I
MK673581	Human	Bangladesh	.	.	D	A	I	P	K	V	.	R	.	D	.	N	.	.	E	.	A	.	R	I
MK673590	Human	Bangladesh	.	.	D	A	I	P	K	V	.	R	.	D	.	N	.	.	E	.	A	.	R	I
FJ513078	Human	India	.	.	D	A	I	P	K	V	.	R	.	D	.	N	K	.	E	.	A	S	R	I
JN808863	Human	Bangladesh	.	.	D	A	I	P	K	V	.	R	.	D	.	N	K	.	E	.	A	.	R	I
MK673592	Human	Bangladesh	.	.	D	A	I	P	K	V	.	R	.	D	.	N	K	.	E	.	A	.	R	I
MK673585	Human	Bangladesh	.	.	D	A	I	P	K	V	.	R	.	D	.	N	K	.	E	.	A	.	R	I
MK673588	Human	Bangladesh	.	.	D	A	I	P	K	V	.	R	.	D	.	N	K	.	E	.	A	.	R	I
MK673575	Human	Bangladesh	.	.	D	A	I	P	K	V	.	R	.	D	.	N	K	.	E	.	A	.	R	I
MK575060	Bat	Bangladesh	.	.	D	A	I	P	K	V	.	R	.	D	.	N	.	.	E	.	A	.	R	I
MK673587	Human	Bangladesh	P	.	D	A	I	P	K	V	.	R	.	D	.	N	.	.	E	.	A	.	R	I
**PP554504**	**Bat**	**India**	**.**	**.**	**D**	**A**	**I**	**P**	**K**	**V**	**.**	**R**	**.**	**D**	**.**	**N**	**K**	**H**	**E**	**.**	**?**	**?**	**?**	**?**
MH523640	Human	India	.	.	D	A	I	P	K	V	.	R	G	D	E	N	.	.	E	.	A	.	R	I
MH523642	Human	India	.	.	D	A	I	P	K	V	.	R	G	D	E	N	.	.	E	.	A	.	R	I
MN549409	Human	India	.	.	D	A	I	P	K	V	.	R	G	D	E	N	.	.	E	.	A	.	R	I
MH523641	Human	India	.	.	D	A	I	P	K	V	.	R	G	D	E	N	.	.	E	.	A	.	R	I
MCL-23- 438	Bat	India	.	.	D	A	I	P	K	V	.	R	G	D	E	N	.	.	E	.	A	.	R	I
MH396625	Human	India	.	.	D	A	I	P	K	V	.	R	G	D	E	N	.	.	E	.	A	.	R	I

Accession ID	Host	Country	P																					
			283	285	286	287	292	294	295	297	298	300	303	304	306	310	311	312	314	316	319	320	327	335
NC002728			**I**	**R**	**T**	**I**	**I**	**D**	**N**	**Q**	**A**	**D**	**S**	**T**	**V**	**G**	**P**	**K**	**S**	**V**	**E**	**P**	**M**	**S**
MK673558	Swine	Malaysia	.	.	.	.	.	.	.	.	.	.	.	.	.	.	.	.	.	.	.	.	.	.
AF212302	Human	Malaysia	.	.	.	.	.	.	.	.	.	.	.	.	.	.	.	.	.	.	.	.	.	.
MK673562	Human	Malaysia	.	.	.	.	.	.	.	.	.	.	.	.	.	.	.	.	.	.	.	.	.	.
AY029767	Human	Malaysia	.	.	.	.	.	.	.	.	.	.	.	.	.	.	.	.	.	.	.	.	.	.
MK673561	Swine	Malaysia	.	.	.	.	.	.	.	.	.	.	.	.	.	.	.	.	.	.	.	.	.	.
MK673560	Swine	Malaysia	.	.	.	.	.	.	.	.	.	.	.	.	.	.	.	.	.	.	.	.	.	.
AJ627196	Swine	Malaysia	.	.	.	.	.	.	.	.	.	.	.	A	.	.	.	.	.	.	.	.	.	.
MK801755	Bat	Cambodia	.	H	.	.	T	E	S	.	.	G	L	A	.	R	.	.	.	.	.	.	.	.
AY988601	Human	Bangladesh	V	H	I	.	T	.	S	.	I	G	P	A	A	.	.	.	.	.	K	S	.	.
MK673564	Human	Bangladesh	V	H	I	.	T	.	S	.	I	G	P	A	A	.	.	.	.	.	K	S	.	.
MK673574	Human	Bangladesh	V	H	I	.	T	.	S	.	I	G	P	A	A	.	.	.	.	.	K	S	.	.
MK673565	Human	Bangladesh	V	H	V	.	T	.	S	.	T	G	P	A	A	.	.	.	P	.	K	S	.	.
MK673572	Human	Bangladesh	V	H	I	.	T	.	S	.	I	G	P	A	A	.	.	.	.	.	K	S	.	.
MK673586	Human	Bangladesh	V	.	I	.	T	.	S	.	I	G	P	A	A	.	.	.	.	.	K	S	.	.
MK673579	Human	Bangladesh	V	.	I	.	T	.	S	.	I	G	P	A	A	.	.	.	.	.	K	S	.	.
MK673580	Human	Bangladesh	V	.	I	.	T	.	S	.	I	G	P	A	A	.	.	.	.	.	K	S	.	.
MK673581	Human	Bangladesh	V	.	I	.	T	.	S	.	I	G	P	A	A	.	.	.	.	.	K	S	.	.
MK673590	Human	Bangladesh	V	H	I	.	T	.	S	.	I	G	P	A	A	.	.	.	.	.	K	S	T	.
FJ513078	Human	India	V	.	I	.	T	.	S	.	I	G	P	A	A	R	.	.	.	.	K	S	.	.
JN808863	Human	Bangladesh	A	.	I	.	T	.	S	.	I	G	P	A	A	.	.	.	.	.	K	S	.	.
MK673592	Human	Bangladesh	V	.	I	.	T	.	S	.	I	G	P	A	A	.	.	.	.	.	K	S	.	.
MK673585	Human	Bangladesh	V	.	I	.	T	.	S	.	I	G	P	A	A	.	.	.	.	.	K	S	.	.
MK673588	Human	Bangladesh	V	.	I	.	T	.	S	.	I	G	P	A	A	.	.	.	.	.	K	S	.	.
MK673575	Human	Bangladesh	V	.	I	.	T	.	S	.	I	G	P	A	A	.	.	.	.	.	K	S	.	.
MK575060	Bat	Bangladesh	V	H	I	.	T	.	S	.	I	.	P	A	A	.	.	R	.	.	K	S	.	.
MK673587	Human	Bangladesh	V	H	I	.	T	.	S	.	I	G	P	A	A	.	.	.	.	.	K	S	.	.
**PP554504**	**Bat**	**India**	**?**	**?**	**?**	**?**	**?**	**.**	**S**	**.**	**I**	**G**	**P**	**A**	**A**	**.**	**.**	**.**	**.**	**.**	**K**	**S**	**.**	**F**
MH523640	Human	India	V	H	I	L	T	.	S	K	I	G	P	A	A	.	L	.	.	.	K	S	.	.
MH523642	Human	India	V	H	I	L	T	.	S	K	I	G	P	A	A	.	L	.	.	.	K	S	.	.
MN549409	Human	India	V	H	I	L	T	.	S	.	I	G	P	A	A	.	L	.	.	.	K	S	.	.
MH523641	Human	India	V	H	I	L	T	.	S	K	I	G	P	A	A	.	L	.	.	.	K	S	.	.
MCL-23- 438	Bat	India	V	H	I	L	T	.	S	.	I	G	P	A	A	.	L	.	.	M	K	S	.	.
MH396625	Human	India	V	H	I	L	T	.	S	K	I	G	P	A	A	.	L	.	.	.	K	S	.	.

Accession ID	Host	Country	P/W/V																				P	
			343	351	354	357	363	365	366	367	370	372	377	378	380	381	382	383	384	386	388	389	410	420
NC002728			R	L	C	G	P	H	W	S	R	I	T	E	V	N	G	A	V	T	D	R	A	V
MK673558	Swine	Malaysia	.	.	.	.	.	.	.	.	.	.	.	.	.	.	.	.	.	.	.	.	.	.
AF212302	Human	Malaysia	.	.	.	.	.	.	.	.	.	.	.	.	.	.	.	.	.	.	.	.	.	.
MK673562	Human	Malaysia	.	.	.	.	.	.	.	.	.	.	.	.	.	.	.	.	.	.	.	.	.	.
AY029767	Human	Malaysia	.	.	.	.	.	.	.	.	.	.	.	.	.	.	.	.	.	.	.	.	.	.
MK673561	Swine	Malaysia	.	.	.	.	.	.	.	.	.	.	.	.	.	.	.	.	.	.	.	.	.	.
MK673560	Swine	Malaysia	.	.	.	.	.	.	.	.	.	.	.	.	.	.	.	.	.	.	.	.	.	.
AJ627196	Swine	Malaysia	.	.	.	.	.	.	.	.	.	.	.	K	.	.	.	.	.	.	.	.	.	.
MK801755	Bat	Cambodia	.	.	.	E	.	.	.	.	K	.	.	.	A	.	S	.	.	.	.	.	.	.
AY988601	Human	Bangladesh	Q	F	S	.	L	Y	R	G	G	R	.	.	T	S	D	.	.	.	N	K	E	.
MK673564	Human	Bangladesh	Q	F	S	.	L	Y	R	G	G	R	.	.	T	S	D	.	.	.	N	K	E	.
MK673574	Human	Bangladesh	Q	F	S	.	L	Y	R	G	G	R	.	.	T	S	D	.	.	.	N	K	E	.
MK673565	Human	Bangladesh	Q	F	S	.	L	Y	R	.	G	R	.	.	T	S	D	.	.	.	N	K	E	.
MK673572	Human	Bangladesh	Q	F	S	.	L	Y	R	G	G	R	.	.	T	S	D	.	.	.	N	K	E	.
MK673586	Human	Bangladesh	Q	F	S	.	L	Y	R	G	G	R	.	.	T	S	D	.	.	.	N	K	E	.
MK673579	Human	Bangladesh	Q	F	S	.	L	Y	R	G	G	R	.	.	T	S	D	.	.	.	N	K	E	.
MK673580	Human	Bangladesh	Q	F	S	.	L	Y	R	G	G	R	.	.	T	S	D	.	.	.	N	K	E	.
MK673581	Human	Bangladesh	Q	F	S	.	L	Y	R	G	G	R	.	.	T	S	D	.	.	.	N	K	E	.
MK673590	Human	Bangladesh	Q	F	S	.	L	Y	R	G	G	R	.	.	T	S	D	.	.	.	N	K	E	.
FJ513078	Human	India	Q	F	S	.	L	Y	R	G	G	R	.	.	T	S	D	.	.	.	N	K	E	.
JN808863	Human	Bangladesh	Q	F	S	.	L	Y	R	G	G	R	A	.	T	S	D	.	.	.	N	K	E	.
MK673592	Human	Bangladesh	Q	F	S	.	L	Y	R	G	G	R	.	.	T	S	D	.	A	.	N	K	E	.
MK673585	Human	Bangladesh	Q	F	S	.	L	Y	R	G	G	R	.	.	T	S	D	.	.	.	N	K	E	.
MK673588	Human	Bangladesh	Q	F	S	.	L	Y	R	G	G	R	.	.	T	S	D	.	.	.	N	K	E	.
MK673575	Human	Bangladesh	Q	F	S	.	L	Y	R	G	G	R	.	.	T	S	D	.	.	.	N	K	E	A
MK575060	Bat	Bangladesh	Q	F	S	.	L	Y	R	G	G	R	.	.	T	S	D	.	.	.	N	K	E	.
MK673587	Human	Bangladesh	Q	F	S	.	L	Y	R	G	G	R	.	.	T	S	D	D	.	.	N	K	E	.
**PP554504**	**Bat**	**India**	**.**	**F**	**S**	**.**	**L**	**Y**	**R**	**G**	**G**	**R**	**.**	**.**	**T**	**S**	**D**	**V**	**.**	**.**	**N**	**K**	**E**	**.**
MH523640	Human	India	Q	F	S	.	L	Y	R	.	G	R	.	.	T	S	D	.	.	N	.	K	E	.
MH523642	Human	India	Q	F	S	.	L	Y	R	.	G	R	.	.	T	S	D	.	.	N	.	K	E	.
MN549409	Human	India	Q	F	S	.	L	Y	R	.	G	R	.	.	T	S	D	.	.	N	.	K	E	.
MH523641	Human	India	Q	F	S	.	L	Y	R	.	G	R	.	.	T	S	D	.	.	N	.	K	E	.
MCL-23- 438	Bat	India	Q	F	S	.	L	Y	R	.	G	R	.	.	T	S	D	.	.	N	.	K	E	.
MH396625	Human	India	Q	F	S	.	L	Y	R	.	G	R	.	.	T	S	D	.	.	N	.	K	E	.

Accession ID	Host	Country	P																		
			421	425	449	452	453	455	458	464	467	482	590	602	629	635	653	655	664	683	687
NC002728			**P**	**S**	**Q**	**A**	**S**	**A**	**E**	**V**	**V**	**D**	**N**	**I**	**A**	**E**	**V**	**M**	**I**	**G**	**K**
MK673558	Swine	Malaysia	.	.	.	.	.	.	.	.	.	.	.	.	.	.	.	.	.	.	.
AF212302	Human	Malaysia	.	.	.	.	.	.	.	.	.	.	.	.	.	.	.	.	.	.	.
MK673562	Human	Malaysia	.	.	.	.	.	.	.	.	.	.	.	.	.	.	.	.	.	.	.
AY029767	Human	Malaysia	.	.	.	.	.	.	.	.	.	.	.	.	.	.	.	.	.	.	.
MK673561	Swine	Malaysia	.	.	.	.	.	.	.	.	.	.	.	.	.	.	.	.	.	.	.
MK673560	Swine	Malaysia	.	.	.	.	.	.	.	.	.	.	.	.	.	.	.	.	.	.	.
AJ627196	Swine	Malaysia	.	.	.	.	.	.	.	.	.	.	.	.	.	.	.	.	.	.	.
MK801755	Bat	Cambodia	.	.	.	.	.	.	.	.	A	N	S	.	.	.	.	.	V	.	.
AY988601	Human	Bangladesh	.	.	.	V	P	V	.	A	A	.	S	V	.	G	.	.	V	.	R
MK673564	Human	Bangladesh	.	.	.	V	P	V	.	A	A	.	S	V	.	G	.	.	V	.	R
MK673574	Human	Bangladesh	.	.	.	V	P	V	.	A	A	.	S	V	.	G	.	.	V	.	R
MK673565	Human	Bangladesh	.	.	.	V	P	V	.	A	A	.	S	V	.	G	.	.	V	.	R
MK673572	Human	Bangladesh	.	.	.	V	P	V	.	A	A	.	S	V	.	G	.	.	V	.	R
MK673586	Human	Bangladesh	.	.	.	V	P	V	.	A	A	.	S	?	.	G	.	.	V	.	R
MK673579	Human	Bangladesh	.	.	.	V	P	V	.	A	A	.	S	V	.	G	.	.	V	.	R
MK673580	Human	Bangladesh	.	.	.	V	P	V	.	A	A	.	S	V	.	G	.	.	V	.	R
MK673581	Human	Bangladesh	.	.	.	V	P	V	.	A	A	.	S	V	.	G	.	.	V	.	R
MK673590	Human	Bangladesh	.	.	.	V	P	V	.	A	A	.	S	V	.	G	.	V	V	.	R
FJ513078	Human	India	.	.	.	V	P	V	.	A	A	.	S	V	.	G	.	.	V	.	R
JN808863	Human	Bangladesh	.	.	.	V	P	V	.	A	A	.	S	V	.	G	.	.	V	.	R
MK673592	Human	Bangladesh	.	.	.	V	P	V	.	A	A	.	S	V	.	G	.	.	V	.	R
MK673585	Human	Bangladesh	.	.	.	V	P	V	.	A	A	.	S	V	.	G	.	.	V	.	R
MK673588	Human	Bangladesh	.	.	.	V	P	V	.	A	A	.	S	V	.	G	.	.	V	.	R
MK673575	Human	Bangladesh	.	.	.	V	P	V	.	A	A	.	S	V	.	G	I	.	V	.	R
MK575060	Bat	Bangladesh	.	.	.	V	P	V	.	A	A	.	S	V	.	G	.	V	V	.	R
MK673587	Human	Bangladesh	.	.	.	V	P	V	.	A	A	.	S	V	.	G	.	V	V	.	R
**PP554504**	**Bat**	**India**	**.**	**.**	**.**	**V**	**P**	**V**	**.**	**A**	**A**	**.**	**S**	**V**	**.**	**?**	**.**	**.**	**V**	**.**	**R**
MH523640	Human	India	L	N	R	V	P	V	.	A	A	.	S	V	T	G	.	.	V	D	R
MH523642	Human	India	L	N	R	V	P	V	.	A	A	.	S	V	T	G	.	.	V	D	R
MN549409	Human	India	.	.	R	V	P	V	G	A	A	.	S	V	T	G	.	.	V	D	R
MH523641	Human	India	L	N	R	V	P	V	.	A	A	.	S	V	T	G	.	.	V	D	R
MCL-23- 438	Bat	India	L	N	R	V	P	V	.	A	A	.	S	V	T	G	.	.	V	D	R
MH396625	Human	India	L	N	R	V	P	V	.	A	A	.	S	V	T	G	.	.	V	D	R

Accession ID	Host	Country	W				V				M							F							
			**416**	**420**	**425**	**449**	**417**	**450**	**453**	**456**	**8**	**13**	**26**	**106**	**127**	**147**	331	2	4	6	9	11	15	19	25
NC002728			G	C	V	N	D	T	F	G	I	M	H	A	I	S	I	V	I	D	C	C	I	M	V
MK673558	Swine	Malaysia	.	.	.	.	.	.	.	.	.	.	.	.	.	.	.	.	.	.	.	.	.	.	.
AF212302	Human	Malaysia	.	.	.	.	.	.	.	.	.	.	.	.	.	.	.	.	.	.	.	.	.	.	.
MK673562	Human	Malaysia	.	.	.	.	.	.	.	.	.	.	.	.	.	.	.	.	.	.	.	.	.	.	.
AY029767	Human	Malaysia	.	.	.	.	.	.	.	.	.	.	.	.	.	.	.	.	.	.	.	.	.	.	.
MK673561	Swine	Malaysia	.	.	.	.	.	.	.	.	.	.	.	.	.	.	.	.	.	.	.	.	.	.	.
MK673560	Swine	Malaysia	.	.	.	.	.	.	.	.	.	.	.	.	.	.	.	.	.	.	.	.	.	.	.
AJ627196	Swine	Malaysia	.	.	.	.	.	.	.	.	.	.	.	.	.	G	.	.	.	.	.	.	.	.	.
MK801755	Bat	Cambodia	.	.	.	.	.	.	.	.	.	.	.	T	.	G	V	.	V	.	.	S	M	.	.
AY988601	Human	Bangladesh	.	.	.	.	.	I	S	C	.	.	N	.	V	G	V	A	.	N	Y	S	.	.	.
MK673564	Human	Bangladesh	.	.	.	.	.	I	S	C	.	.	N	.	V	G	V	A	.	N	Y	S	.	.	.
MK673574	Human	Bangladesh	.	.	.	.	.	I	S	C	.	.	N	.	V	G	V	A	.	N	Y	S	.	.	.
MK673565	Human	Bangladesh	.	.	.	.	.	I	S	C	.	.	N	.	V	G	V	A	.	N	Y	S	.	.	.
MK673572	Human	Bangladesh	.	.	.	.	.	I	S	C	.	.	N	.	V	G	V	A	.	N	Y	S	.	.	.
MK673586	Human	Bangladesh	.	.	.	.	.	I	S	C	.	.	N	.	V	G	V	A	.	N	Y	S	.	I	.
MK673579	Human	Bangladesh	.	.	.	.	.	I	S	C	.	.	N	.	V	G	V	A	.	N	Y	S	.	.	.
MK673580	Human	Bangladesh	.	.	.	.	.	I	S	C	.	.	N	.	V	G	V	A	.	N	Y	S	.	.	.
MK673581	Human	Bangladesh	.	.	.	.	.	I	S	C	.	.	N	.	V	G	V	A	.	N	Y	S	.	.	.
MK673590	Human	Bangladesh	.	.	.	.	.	I	S	C	.	.	N	.	V	G	V	A	.	N	Y	S	.	.	.
FJ513078	Human	India	.	.	.	.	.	I	S	C	V	I	N	.	V	G	V	A	.	N	Y	S	.	I	.
JN808863	Human	Bangladesh	.	.	.	.	.	I	S	C	.	.	N	.	V	G	V	A	.	N	Y	S	.	I	.
MK673592	Human	Bangladesh	E	.	.	.	N	I	S	C	.	.	N	.	V	G	V	A	.	N	Y	S	.	I	.
MK673585	Human	Bangladesh	.	.	.	.	.	I	S	C	.	.	N	.	V	G	V	A	.	N	Y	S	.	I	.
MK673588	Human	Bangladesh	.	.	.	.	.	I	S	C	.	.	N	.	V	G	V	A	.	N	Y	S	.	I	I
MK673575	Human	Bangladesh	.	R	.	.	.	I	S	C	.	.	N	.	V	G	V	A	.	N	Y	S	.	I	.
MK575060	Bat	Bangladesh	.	.	.	.	.	I	S	C	.	.	N	.	V	G	V	A	.	N	Y	S	.	.	.
MK673587	Human	Bangladesh	.	.	.	.	.	I	S	C		.	N	.	V	G	V	A	.	N	Y	S	.	.	.
PP554504	Bat	**India**	.	.	.	.	.	I	S	C		.	N	.	V	G	V	A	.	N	Y	S	.	I	.
MH523640	Human	India	.	.	M	D	.	I	S	C		.	N	.	V	G	V	A	.	N	Y	S	L	.	.
MH523642	Human	India	.	.	M	D	.	I	S	C		.	N	.	V	G	V	A	.	N	Y	S	L	.	.
MN549409	Human	India	.	.	.	D	.	I	S	C		.	N	.	V	G	V	A	.	N	Y	S	L	.	.
MH523641	Human	India	.	.	M	D	.	I	S	C		.	N	.	V	G	V	A	.	N	Y	S	L	.	.
MCL-23- 438	Bat	India	.	.	M	D	.	I	S	C		.	N	.	V	G	V	A	.	N	Y	S	L	.	.
MH396625	Human	India	.	.	M	D	.	I	S	C		.	N	.	V	G	V	A	.	N	Y	S	L	.	.

Accession ID	Host	Country	F												G									
			**36**	**42**	**159**	**207**	**249**	**250**	**252**	**273**	**298**	**300**	**301**	**302**	**3**	**5**	**14**	**20**	**24**	**82**	**89**	**135**	**172**	**228**
NC002728			**I**	**V**	**V**	**L**	**A**	**T**	**D**	**S**	**P**	**S**	**F**	**N**	**A**	**N**	**T**	**I**	**V**	**V**	**G**	**T**	**R**	**Y**
MK673558	Swine	Malaysia	.	.	.	.	.	.	.	.	.	.	.	.	.	.	.	.	.	.	.	.	.	.
AF212302	Human	Malaysia	.	.	.	.	.	.	.	.	.	.	.	.	.	.	.	.	.	.	.	.	.	.
MK673562	Human	Malaysia	.	.	.	.	.	.	.	.	.	.	.	.	.	.	.	.	.	.	.	.	.	.
AY029767	Human	Malaysia	.	.	.	.	.	.	.	.	.	.	.	.	.	.	.	.	.	.	.	.	.	.
MK673561	Swine	Malaysia	.	.	.	.	.	.	.	.	.	.	.	.	.	.	.	.	.	.	.	.	.	.
MK673560	Swine	Malaysia	.	.	.	.	.	.	.	.	.	.	.	.	.	.	.	.	.	.	.	.	.	.
AJ627196	Swine	Malaysia	.	.	.	.	.	I	.	.	.	.	.	.	.	.	.	N	.	.	.	.	.	.
MK801755	Bat	Cambodia	.	I	I	.	.	.	.	.	.	.	.	.	.	S	.	.	I	.	.	.	.	.
AY988601	Human	Bangladesh	.	I	.	S	.	.	G	G	.	.	.	.	T	S	A	N	.	M	S	.	K	.
MK673564	Human	Bangladesh	.	I	.	.	.	.	.	G	.	.	.	.	T	S	A	N	.	M	S	.	K	.
MK673574	Human	Bangladesh	.	I	.	.	.	.	.	G	.	.	.	.	T	S	A	N	.	M	S	.	K	H
MK673565	Human	Bangladesh	.	I	.	.	.	.	.	G	.	.	.	.	T	S	A	N	.	M	S	.	K	.
MK673572	Human	Bangladesh	.	I	.	.	.	.	.	G	.	.	.	.	T	S	A	N	.	M	S	.	K	H
MK673586	Human	Bangladesh	.	I	.	.	.	.	.	G	.	.	.	.	T	S	A	N	.	M	S	.	K	.
MK673579	Human	Bangladesh	V	I	.	.	T	.	.	G	.	.	.	.	T	S	A	N	.	I	S	.	K	.
MK673580	Human	Bangladesh	V	I	.	.	T	.	.	G	.	.	.	.	T	S	A	N	.	I	S	.	K	?
MK673581	Human	Bangladesh	.	I	.	.	.	.	.	G	.	.	.	.	T	S	A	N	.	I	S	.	K	.
MK673590	Human	Bangladesh	.	I	.	.	.	.	.	G	.	.	.	.	T	S	A	N	.	M	S	.	K	.
FJ513078	Human	India	.	I	.	.	.	.	.	G	.	.	.	.	T	S	A	N	.	M	S	.	K	.
JN808863	Human	Bangladesh	.	I	.	.	.	.	.	G	.	.	.	.	T	S	A	N	.	M	S	.	K	.
MK673592	Human	Bangladesh	.	I	.	.	.	.	.	G	.	.	.	.	T	S	A	N	.	M	S	.	K	.
MK673585	Human	Bangladesh	.	I	.	.	.	.	.	G	.	.	.	.	T	S	A	N	.	M	S	.	K	.
MK673588	Human	Bangladesh	.	I	.	.	.	.	.	G	.	.	.	.	T	S	A	N	.	M	S	.	K	.
MK673575	Human	Bangladesh	.	I	.	.	.	.	.	G	.	.	.	.	T	S	A	N	.	M	S	.	K	.
MK575060	Bat	Bangladesh	.	I	.	.	.	.	.	G	.	.	.	.	T	S	A	N	.	M	S	.	K	.
MK673587	Human	Bangladesh	.	I	.	.	.	.	.	G	.	.	.	.	T	S	A	N	.	M	S	.	K	.
**PP554504**	**Bat**	**India**	**.**	**I**	**.**	**.**	**.**	**.**	**.**	**G**	**H**	**R**	**Y**	**K**	**T**	**S**	**A**	**N**	**.**	**M**	**S**	**.**	**K**	**.**
MH523640	Human	India	.	I	.	.	.	.	.	G	.	.	.	.	T	S	A	N	.	M	S	.	K	.
MH523642	Human	India	.	I	.	.	.	.	.	G	.	.	.	.	T	S	A	N	.	M	S	.	K	.
MN549409	Human	India	.	I	.	.	.	.	.	G	.	.	.	.	T	S	A	N	.	M	S	A	K	.
MH523641	Human	India	.	I	.	.	.	.	.	G	.	.	.	.	T	S	A	N	.	M	S	.	K	.
MCL-23- 438	Bat	India	.	I	.	.	.	.	.	G	.	.	.	.	T	S	A	N	.	M	S	.	K	.
MH396625	Human	India	.	I	.	.	.	.	.	G	.	.	.	.	T	S	A	N	.	M	S	.	K	.

Accession ID	Host	Country	G																					
			**236**	**248**	**272**	**274**	**288**	**299**	**304**	**325**	**327**	**328**	**329**	**335**	**339**	**344**	**376**	**381**	**384**	**385**	**386**	**397**	**404**	**408**
NC002728			**R**	**R**	**T**	**P**	**N**	**T**	**I**	**S**	**G**	**G**	**G**	**L**	**S**	**R**	**K**	**N**	**I**	**T**	**K**	**L**	**N**	**I**
MK673558	Swine	Malaysia	.	.	.	.	.	.	.	.	.	.	.	.	.	.	.	.	.	.	.	.	.	.
AF212302	Human	Malaysia	.	.	.	.	.	.	.	.	.	.	.	.	.	.	.	.	.	.	.	.	.	.
MK673562	Human	Malaysia	.	.	.	.	.	.	.	.	.	.	.	.	.	.	.	.	.	.	.	.	.	.
AY029767	Human	Malaysia	.	.	.	.	.	.	.	.	.	.	.	.	.	.	.	.	.	.	.	.	.	.
MK673561	Swine	Malaysia	.	.	.	.	.	.	.	.	.	.	.	.	.	.	.	.	.	.	.	.	.	.
MK673560	Swine	Malaysia	.	.	.	.	.	.	.	.	.	.	.	.	.	.	.	.	.	.	.	.	.	.
AJ627196	Swine	Malaysia	.	.	A	.	.	.	.	.	.	.	.	.	.	.	.	.	.	.	.	.	.	.
MK801755	Bat	Cambodia	.	K	.	.	.	.	.	.	D	.	.	.	.	.	.	.	.	.	.	.	.	V
AY988601	Human	Bangladesh	K	.	.	S	.	V	.	N	.	E	S	F	N	K	.	.	.	A	E	.	.	.
MK673564	Human	Bangladesh	K	.	.	S	.	V	.	N	.	E	S	F	N	K	.	.	.	A	E	.	.	.
MK673574	Human	Bangladesh	K	.	.	S	.	V	.	N	.	E	S	F	N	K	.	.	.	A	E	.	.	.
MK673565	Human	Bangladesh	K	.	.	S	.	V	.	N	.	E	S	F	N	K	.	.	.	A	E	.	.	.
MK673572	Human	Bangladesh	K	.	.	S	.	V	.	N	.	E	S	F	N	K	.	.	.	A	E	.	.	.
MK673586	Human	Bangladesh	K	.	.	S	.	V	.	N	.	E	S	F	N	K	.	S	.	A	E	.	.	.
MK673579	Human	Bangladesh	K	.	.	S	.	V	.	N	.	E	S	F	N	K	.	.	.	A	E	.	.	.
MK673580	Human	Bangladesh	K	.	.	S	.	V	.	N	.	E	S	F	N	K	.	.	.	A	E	.	.	.
MK673581	Human	Bangladesh	K	.	.	S	.	V	.	N	.	E	S	F	N	K	.	.	.	A	E	.	.	.
MK673590	Human	Bangladesh	K	.	.	S	.	V	.	N	.	E	S	F	N	K	T	.	.	A	E	.	S	.
FJ513078	Human	India	K	.	.	S	.	V	V	N	.	E	S	F	N	K	.	.	.	A	E	.	.	.
JN808863	Human	Bangladesh	K	.	.	S	.	V	.	N	.	E	S	F	N	K	.	.	.	A	E	.	.	.
MK673592	Human	Bangladesh	K	.	.	S	.	V	.	N	.	E	S	F	N	K	.	.	.	A	E	.	.	.
MK673585	Human	Bangladesh	K	.	.	S	.	V	.	N	.	E	S	F	N	K	.	S	.	A	E	.	.	.
MK673588	Human	Bangladesh	K	.	.	S	.	V	.	.	.	E	S	F	N	K	.	.	.	A	E	.	.	.
MK673575	Human	Bangladesh	K	.	.	S	.	V	.	.	.	E	S	F	N	K	.	.	.	A	E	.	.	.
MK575060	Bat	Bangladesh	K	.	.	S	.	V	.	N	.	E	S	F	N	K	T	.	.	A	E	.	.	.
MK673587	Human	Bangladesh	K	.	.	S	.	V	.	N	.	E	S	F	N	K	T	.	.	A	E	.	.	.
**PP554504**	**Bat**	**India**	**K**	**.**	**.**	**S**	**.**	**V**	**.**	**N**	**.**	**E**	**S**	**F**	**N**	**K**	**.**	**?**	**?**	**?**	**?**	**.**	**.**	**.**
MH523640	Human	India	K	.	.	S	S	V	.	N	.	E	S	F	N	M	.	.	V	A	.	.	.	.
MH523642	Human	India	K	.	.	S	S	V	.	N	.	E	S	F	N	M	.	.	V	A	.	.	.	.
MN549409	Human	India	K	.	.	S	S	V	.	N	.	E	S	F	N	M	.	.	V	A	.	.	.	.
MH523641	Human	India	K	.	.	S	S	V	.	N	.	E	S	F	N	M	.	.	V	A	.	.	.	.
MCL-23- 438	Bat	India	K	.	.	S	S	V	.	N	.	E	S	F	N	M	.	.	V	A	.	R	.	.
MH396625	Human	India	K	.	.	S	S	V	.	N	.	E	S	F	N	M	.	.	V	A	.	.	.	.

Accession ID	Host	Country	G													L								
			**421**	**423**	**424**	**426**	**427**	**440**	**470**	**478**	**481**	**498**	**502**	**545**	**569**	**36**	**71**	**77**	**83**	**94**	**112**	**140**	**190**	**223**
NC002728			**G**	**N**	**P**	**V**	**V**	**S**	**L**	**N**	**N**	**T**	**I**	**I**	**K**	**R**	**D**	**I**	**F**	**I**	**K**	**D**	**P**	**T**
MK673558	Swine	Malaysia	.	.	.	.	.	.	.	.	.	.	.	.	.	.	.	.	.	.	.	.	.	.
AF212302	Human	Malaysia	.	.	.	.	.	.	.	.	.	.	.	.	.	.	.	.	.	.	.	.	.	.
MK673562	Human	Malaysia	.	.	.	.	.	.	.	.	.	.	.	.	.	.	.	.	.	.	.	.	.	.
AY029767	Human	Malaysia	.	.	.	.	.	.	.	.	.	.	.	.	.	.	.	.	.	.	.	.	.	.
MK673561	Swine	Malaysia	.	.	.	.	.	.	.	.	.	.	.	.	.	.	.	.	.	.	.	.	.	.
MK673560	Swine	Malaysia	.	.	.	.	.	.	.	.	.	.	.	.	.	.	.	.	.	.	.	.	.	.
AJ627196	Swine	Malaysia	.	.	.	.	.	.	.	.	.	.	.	.	.	.	.	.	.	.	.	.	.	N
MK801755	Bat	Cambodia	.	.	.	I	.	.	Q	S	D	.	.	.	.	.	.	.	.	T	.	.	.	.
AY988601	Human	Bangladesh	E	.	S	I	.	.	Q	.	D	K	V	V	.	K	N	V	.	T	R	.	.	.
MK673564	Human	Bangladesh	E	.	S	I	.	.	Q	.	D	K	V	V	.	K	N	V	.	T	R	.	.	.
MK673574	Human	Bangladesh	E	.	S	I	.	.	Q	.	D	K	V	V	.	K	N	V	.	T	R	.	.	.
MK673565	Human	Bangladesh	E	.	S	I	.	.	Q	.	D	K	.	V	R	K	N	V	.	T	R	.	.	.
MK673572	Human	Bangladesh	E	.	S	I	.	.	Q	.	D	K	V	V	.	K	N	V	.	T	R	.	.	.
MK673586	Human	Bangladesh	E	.	S	I	.	.	Q	.	D	K	V	V	.	K	N	V	.	.	.	.	L	.
MK673579	Human	Bangladesh	E	.	S	I	.	.	Q	D	D	K	V	V	.	K	N	V	.	.	.	.	.	.
MK673580	Human	Bangladesh	E	.	S	I	.	P	Q	D	D	K	V	V	.	K	N	V	.	.	.	G	.	.
MK673581	Human	Bangladesh	E	.	S	I	.	.	Q	D	D	K	V	V	.	K	N	V	Y	.	.	.	.	.
MK673590	Human	Bangladesh	E	.	S	I	.	.	Q	.	D	K	V	V	.	K	N	V	.	T	R	.	.	.
FJ513078	Human	India	E	.	S	I	.	.	Q	.	D	K	V	V	.	K	N	V	.	.	.	.	.	.
JN808863	Human	Bangladesh	E	.	S	I	.	.	Q	.	D	K	V	V	.	K	N	V	.	.	.	.	.	.
MK673592	Human	Bangladesh	E	.	S	I	.	.	Q	.	D	K	V	V	.	K	N	V	.	.	.	.	.	.
MK673585	Human	Bangladesh	E	.	S	I	.	.	Q	.	D	K	V	V	.	K	N	V	.	.	.	.	.	.
MK673588	Human	Bangladesh	E	.	S	I	.	.	Q	.	D	K	V	V	.	K	N	V	.	.	.	.	.	.
MK673575	Human	Bangladesh	E	.	S	I	.	.	Q	.	D	K	V	V	.	K	N	V	.	.	.	.	.	.
MK575060	Bat	Bangladesh	E	.	S	I	.	.	Q	.	D	K	V	V	.	K	N	V	.	T	R	.	.	.
MK673587	Human	Bangladesh	E	.	S	I	.	.	Q	.	D	K	V	V	.	K	N	V	.	T	R	.	.	.
**PP554504**	**Bat**	**India**	**E**	**.**	**S**	**I**	**.**	**.**	**Q**	**.**	**D**	**K**	**V**	**V**	**.**	**K**	**?**	**?**	**?**	**.**	**.**	**.**	**.**	**.**
MH523640	Human	India	E	.	S	I	I	.	Q	.	D	.	V	V	.	K	N	V	.	T	.	.	.	.
MH523642	Human	India	E	.	S	I	I	.	Q	.	D	.	V	V	.	K	N	V	.	T	.	.	.	.
MN549409	Human	India	E	D	S	I	I	.	Q	.	D	.	V	V	.	K	N	V	.	T	.	.	.	.
MH523641	Human	India	E	.	S	I	I	.	Q	.	D	.	V	V	.	K	N	V	.	T	.	.	.	.
MCL-23- 438	Bat	India	E	.	S	I	I	.	Q	.	D	.	V	V	.	K	N	V	.	T	.	.	.	.
MH396625	Human	India	E	.	S	I	I	.	Q	.	D	.	V	V	.	K	N	V	.	T	.	.	.	.

Accession ID	Host	Country	L																					
			**252**	**325**	**422**	**514**	**533**	**621**	**623**	**625**	**632**	**633**	**634**	**636**	**639**	**640**	**642**	**652**	**658**	**661**	**665**	**703**	**760**	**763**
NC002728			**V**	**E**	**A**	**N**	**E**	**R**	**F**	**Y**	**N**	**V**	**K**	**K**	**N**	**S**	**N**	**C**	**H**	**M**	**T**	**R**	**E**	**V**
MK673558	Swine	Malaysia															.	.	.	.	.	.	.	.
AF212302	Human	Malaysia															.	.	.	.	.	.	.	.
MK673562	Human	Malaysia															.	.	.	.	.	.	.	.
AY029767	Human	Malaysia															.	.	.	.	.	.	.	.
MK673561	Swine	Malaysia															.	.	.	.	.	.	.	.
MK673560	Swine	Malaysia															.	.	.	.	.	.	.	.
AJ627196	Swine	Malaysia															.	.	.	.	.	.	.	.
MK801755	Bat	Cambodia															.	.	.	.	.	.	.	.
AY988601	Human	Bangladesh	I	.	.	.	D	K	.	C	S	.	.	.	D	P	Y	.	Y	.	I	K	.	.
MK673564	Human	Bangladesh	I	.	.	.	D	K	.	C	S	.	.	.	D	P	Y	.	Y	.	I	K	.	.
MK673574	Human	Bangladesh	I	.	.	.	D	K	.	C	S	.	.	.	D	P	.	.	Y	.	I	K	.	.
MK673565	Human	Bangladesh	I	D	.	.	D	K	.	C	.	.	.	.	D	P	Y	.	Y	.	I	K	.	.
MK673572	Human	Bangladesh	I	.	.	.	D	K	.	C	S	.	.	.	D	P	.	.	Y	.	I	K	.	.
MK673586	Human	Bangladesh	I	.	.	.	D	K	.	C	.	.	.	.	.	P	Y	.	Y	.	.	K	.	.
MK673579	Human	Bangladesh	I	.	.	.	D	K	.	C	.	.	.	.	.	P	Y	.	Y	.	.	K	.	.
MK673580	Human	Bangladesh	I	.	.	.	D	K	.	C	.	.	.	.	.	P	Y	.	Y	.	.	K	.	.
MK673581	Human	Bangladesh	I	.	.	.	D	K	.	C	.	.	.	.	.	P	Y	.	Y	.	.	K	.	.
MK673590	Human	Bangladesh	I	.	.	.	D	K	.	C	S	M	.	.	D	P	.	F	Y	.	I	K	.	.
FJ513078	Human	India	I	.	.	.	D	K	.	C	.	.	.	.	.	P	Y	.	Y	.	.	K	.	.
JN808863	Human	Bangladesh	I	.	.	.	D	K	.	C	.	.	.	.	.	P	Y	.	Y	.	.	K	.	.
MK673592	Human	Bangladesh	I	.	.	.	D	K	.	C	.	.	.	.	.	P	Y	.	Y	.	.	K	.	.
MK673585	Human	Bangladesh	I	.	.	.	D	K	.	C	.	.	.	.	.	P	Y	.	Y	.	.	K	.	.
MK673588	Human	Bangladesh	I	.	.	.	D	K	.	C	.	.	.	.	.	P	Y	.	Y	.	.	K	.	.
MK673575	Human	Bangladesh	I	.	.	.	D	K	.	C	.	.	.	.	.	P	Y	.	Y	.	.	K	.	.
MK575060	Bat	Bangladesh	I	.	.	.	D	K	.	C	S	M	.	.	D	P	.	.	Y	.	I	K	D	.
MK673587	Human	Bangladesh	I	.	.	.	D	K	.	C	S	.	.	.	D	P	.	.	Y	.	I	K	.	.
**PP554504**	**Bat**	**India**	?	.	.	.	D	K	.	C	.	.	.	.	.	P	Y	.	Y	.	.	K	.	.
MH523640	Human	India	I	.	.	.	D	K	.	C	.	.	.	.	.	P	Y	.	Y	.	.	K	.	.
MH523642	Human	India	I	.	.	.	D	K	.	C	.	.	.	.	.	P	Y	.	Y	.	.	K	.	.
MN549409	Human	India	I	.	.	.	D	K	.	C	.	.	.	.	.	P	Y	.	.	.	.	K	.	.
MH523641	Human	India	I	.	.	.	D	K	.	C	.	.	.	.	.	P	Y	.	Y	.	.	K	.	.
MCL-23- 438	Bat	India	I	.	.	S	D	K	.	C	.	.	.	.	.	P	Y	.	Y	**T**	.	K	.	**I**
MH396625	Human	India	I	.	.	.	D	K	.	C	.	.	.	.	.	P	Y	.	Y	.	.	K	.	.

Accession ID	Host	Country	L																					
			783	890	909	1154	1157	1181	1262	1440	1452	1494	1551	1558	1577	1645	1658	1707	1722	1748	1753	1791	1793	1801
NC002728			**K**	**I**	**M**	**L**	**K**	**R**	**R**	**D**	**D**	**V**	**S**	**I**	**V**	**S**	**S**	**V**	**V**	**I**	**M**	**A**	**K**	**R**
MK673558	Swine	Malaysia	.	.	.	.	.	.	.	.	.	.	.	.	.	.	.	.	.	.	.	.	.	.
AF212302	Human	Malaysia	.	.	.	.	.	.	.	.	.	.	.	.	.	.	.	.	.	.	.	.	.	.
MK673562	Human	Malaysia	.	.	.	.	.	.	.	.	.	.	.	.	.	.	.	.	.	.	.	.	.	.
AY029767	Human	Malaysia	.	.	.	.	.	.	.	.	.	.	.	.	.	F	.	.	.	.	.	.	.	.
MK673561	Swine	Malaysia	.	.	.	.	.	.	.	.	.	.	.	.	.	.	.	.	.	.	.	.	E	.
MK673560	Swine	Malaysia	.	.	.	.	.	.	.	.	.	.	.	.	.	.	.	.	.	.	.	.	E	.
AJ627196	Swine	Malaysia	.	.	.	.	.	.	.	.	.	.	.	.	.	F	.	.	.	.	V	.	.	.
MK801755	Bat	Cambodia	E	V	V	I	.	.	.	.	.	.	.	.	.	F	.	.	.	.	.	.	.	.
AY988601	Human	Bangladesh	E	V	.	I	R	K	K	.	.	A	A	.	I	Y	N	M	.	V	.	S	.	K
MK673564	Human	Bangladesh	E	V	.	I	R	K	K	.	.	A	A	.	I	Y	N	M	.	V	.	S	.	K
MK673574	Human	Bangladesh	E	V	.	I	R	K	K	.	.	A	A	.	I	Y	N	M	.	V	.	S	.	K
MK673565	Human	Bangladesh	E	V	.	I	R	K	K	.	.	A	A	.	I	Y	N	M	.	.	.	S	.	K
MK673572	Human	Bangladesh	E	V	.	I	R	K	K	.	.	A	A	.	I	Y	N	M	.	V	.	S	.	K
MK673586	Human	Bangladesh	E	V	.	I	R	K	K	.	.	A	A	.	I	Y	N	M	.	.	.	S	.	K
MK673579	Human	Bangladesh	E	V	.	I	R	K	K	.	.	A	A	.	I	Y	N	M	.	.	.	S	.	K
MK673580	Human	Bangladesh	E	V	?	I	R	K	K	.	.	A	A	.	I	Y	N	M	.	.	.	S	.	K
MK673581	Human	Bangladesh	E	V	.	I	R	K	K	.	.	A	A	.	I	Y	N	M	.	.	.	S	.	K
MK673590	Human	Bangladesh	E	V	.	I	R	K	K	.	.	A	A	.	I	Y	N	M	A	.	.	S	.	K
FJ513078	Human	India	E	V	.	I	R	K	K	.	.	A	A	.	I	Y	N	M	.	.	.	S	.	K
JN808863	Human	Bangladesh	E	V	.	I	R	K	K	.	.	A	A	.	I	Y	N	M	.	.	.	S	.	K
MK673592	Human	Bangladesh	E	V	.	I	R	K	K	.	.	A	A	.	I	Y	N	M	.	.	.	S	.	K
MK673585	Human	Bangladesh	E	V	.	I	R	K	K	.	.	A	A	.	I	Y	N	M	.	.	.	S	.	K
MK673588	Human	Bangladesh	E	V	.	I	R	K	K	N	.	A	A	.	I	Y	N	M	.	.	.	S	.	K
MK673575	Human	Bangladesh	E	V	.	I	R	K	K	.	.	A	A	.	I	Y	N	M	.	.	.	S	.	K
MK575060	Bat	Bangladesh	E	V	.	I	R	K	K	.	.	A	A	.	I	Y	N	M	.	.	.	S	.	K
MK673587	Human	Bangladesh	E	V	.	I	R	K	K	.	.	A	A	V	I	Y	N	M	.	V	.	S	.	K
**PP554504**	**Bat**	**India**	E	V	.	I	R	K	K	.	.	A	A	.	I	Y	N	M	.	.	.	S	.	?
MH523640	Human	India	E	V	.	I	R	K	.	.	.	A	A	.	I	Y	N	M	.	.	.	S	.	K
MH523642	Human	India	E	V	.	I	R	K	.	.	.	A	A	.	I	Y	N	M	.	.	.	S	.	K
MN549409	Human	India	E	V	.	I	R	K	.	.	.	A	A	.	I	Y	N	M	.	.	.	S	.	K
MH523641	Human	India	E	V	.	I	R	K	.	.	.	A	A	.	I	Y	N	M	.	.	.	S	.	K
MCL23438	Bat	India	E	V	.	I	R	K	.	.	N	A	A	.	I	Y	N	M	.	.	.	S	.	K
MH396625	Human	India	E	V	.	I	R	K	.	.	.	A	A	.	I	Y	N	M	.	.	.	S	.	K

Accession ID	Host	Country	L															
			**1894**	**1896**	**1950**	**1954**	**2001**	**2027**	**2031**	**2037**	**2039**	**2064**	**2071**	**2087**	**2091**	**2153**	**2159**	**2216**
NC002728			**S**	**T**	**E**	**V**	**V**	**V**	**K**	**N**	**H**	**D**	**Q**	**Q**	**Q**	**I**	**C**	**N**
MK673558	Swine	Malaysia	.	.	.	.	.	.	.	.	.	.	.	.	.	V	.	.
AF212302	Human	Malaysia	.	.	.	.	.	.	.	.	.	.	.	.	.	.	.	.
MK673562	Human	Malaysia	.	.	.	.	.	.	.	.	.	.	.	.	.	.	.	.
AY029767	Human	Malaysia	.	.	.	.	.	.	.	.	.	.	.	.	.	.	.	.
MK673561	Swine	Malaysia	.	.	.	.	.	.	.	.	.	.	.	.	.	.	.	.
MK673560	Swine	Malaysia	.	.	.	.	.	.	.	.	.	.	.	.	.	.	.	.
AJ627196	Swine	Malaysia	.	.	.	.	.	.	.	.	N	.	.	.	.	.	.	.
MK801755	Bat	Cambodia	P	.	.	.	.	.	.	.	.	.	.	.	H	.	R	S
AY988601	Human	Bangladesh	.	A	.	.	.	I	R	D	.	E	H	.	.	.	R	S
MK673564	Human	Bangladesh	.	A	.	.	.	I	R	D	.	E	H	.	.	.	R	S
MK673574	Human	Bangladesh	.	A	.	.	.	I	R	D	.	E	H	.	.	.	R	S
MK673565	Human	Bangladesh	.	A	.	.	.	I	R	D	.	E	H	.	.	.	R	S
MK673572	Human	Bangladesh	.	A	.	.	.	I	R	D	.	E	H	.	.	.	R	S
MK673586	Human	Bangladesh	.	A	.	.	.	I	R	D	.	E	H	.	.	.	R	S
MK673579	Human	Bangladesh	.	A	.	.	.	I	R	D	.	E	H	.	.	.	R	S
MK673580	Human	Bangladesh	.	A	.	.	.	I	R	D	.	E	H	.	.	.	R	S
MK673581	Human	Bangladesh	.	A	.	.	.	I	R	D	.	E	H	.	.	.	R	S
MK673590	Human	Bangladesh	.	A	.	.	.	I	R	D	.	E	H	.	.	.	R	S
FJ513078	Human	India	.	A	.	.	.	I	R	D	.	E	H	.	.	.	R	S
JN808863	Human	Bangladesh	.	A	.	.	M	I	R	D	.	E	H	.	.	.	R	S
MK673592	Human	Bangladesh	.	A	.	.	.	I	R	D	.	E	H	.	.	.	R	S
MK673585	Human	Bangladesh	.	A	.	.	.	I	R	D	.	E	H	.	.	.	R	S
MK673588	Human	Bangladesh	.	A	.	.	.	I	R	D	.	E	H	.	.	.	R	S
MK673575	Human	Bangladesh	.	A	.	.	.	I	R	D	.	E	H	.	.	.	R	S
MK575060	Bat	Bangladesh	.	A	.	.	.	I	R	D	.	E	H	.	.	.	R	S
MK673587	Human	Bangladesh	.	A	.	I	.	I	R	D	.	E	H	.	.	.	R	S
**PP554504**	**Bat**	**India**	.	A	.	.	.	I	R	D	.	E	H	**R**	.	.	R	S
MH523640	Human	India	.	A	.	.	.	I	R	.	.	E	H	.	.	.	R	S
MH523642	Human	India	.	A	.	.	.	I	R	.	.	E	H	.	.	.	R	S
MN549409	Human	India	.	A	G	.	.	I	R	.	.	E	H	.	.	.	R	S
MH523641	Human	India	.	A	.	.	.	I	R	.	.	E	H	.	.	.	R	S
MCL23438	Bat	India	.	A	.	.	.	I	R	.	.	E	H	.	.	.	R	S
MH396625	Human	India	.	A	.	.	.	I	R	.	.	E	H	.	.	.	R	S

Supplementary table 3: The neutralization titre and optical density values in ELISA of serum samples

Sr no	Location of sample collection	Sample ID	Neutralization titre	Optical Density (ELISA)
1	Meghalaya	MCL-22-B-148/3	40	0.595
2	Meghalaya	MCL-22-B-156/3	640	1.64
3	Meghalaya	MCL-22-B-176/3	0	0.146
4	Meghalaya	MCL-22-B-186/3	0	0.256
5	Assam	MCL-22-B-194/3	0	0.222
6	Assam	MCL-22-B-193/3	0	0.255
7	Assam	MCL-22-B-196/3	40	0.454
8	Assam	MCL-22-B-203/3	80	1.02
9	West Bengal	MCL-23-B-184/3	80	0.702
10	West Bengal	MCL-23-B-191/3	0	0.281
11	West Bengal	MCL-23-B-190/3	40	0.456
12	West Bengal	MCL-23-B-193/3	40	0.949
13	West Bengal	MCL-23-B-194/3	320	1.221
14	West Bengal	MCL-23-B-195/3	320	1.157
15	West Bengal	MCL-23-B-198/3	160	0.668
16	West Bengal	MCL-23-B-199/3	320	1.056
17	West Bengal	MCL-23-B-200/3	80	0.759
18	Bihar	MCL-23-B-247/3	160	0.859
19	Bihar	MCL-23-B-254/3	0	0.202
20	Bihar	MCL-23-B-260/3	320	1.215
21	Bihar	MCL-23-B-272/3	160	0.964
22	Bihar	MCL-23-B-277/3	80	0.926
23	Bihar	MCL-23-B-278/3	640	1.492
24	Bihar	MCL-23-B-284/3	0	0.181
25	Bihar	MCL-23-B-287/3	320	1.376
26	Bihar	MCL-23-B-288/3	320	1.286
